# The tertiary structure of the human Xkr8–Basigin complex that scrambles phospholipids at plasma membranes

**DOI:** 10.1038/s41594-021-00665-8

**Published:** 2021-10-08

**Authors:** Takaharu Sakuragi, Ryuta Kanai, Akihisa Tsutsumi, Hirotaka Narita, Eriko Onishi, Kohei Nishino, Takuya Miyazaki, Takeshi Baba, Hidetaka Kosako, Atsushi Nakagawa, Masahide Kikkawa, Chikashi Toyoshima, Shigekazu Nagata

**Affiliations:** 1 Laboratory of Biochemistry and Immunology, World Premier International Immunology Frontier Research Center, Osaka University, Suita, Japan; 2 Institute for Quantitative Biosciences, The University of Tokyo, Tokyo, Japan; 3 Department of Cell Biology and Anatomy, Graduate School of Medicine, The University of Tokyo, Tokyo, Japan; 4 Institute for Protein Research, Osaka University, Suita, Japan; 5 Division of Cell Signaling, Fujii Memorial Institute of Medical Sciences, Tokushima University, Tokushima, Japan; 6 Research Division, Chugai Pharmaceutical Co., Ltd., Kamakura, Japan; 7 Present Address: Japan Aerospace Exploration Agency (JAXA), Tsukuba, Japan

**Keywords:** Cryoelectron microscopy, Apoptosis

## Abstract

Xkr8–Basigin is a plasma membrane phospholipid scramblase activated by kinases or caspases. We combined cryo-EM and X-ray crystallography to investigate its structure at an overall resolution of 3.8 Å. Its membrane-spanning region carrying 22 charged amino acids adopts a cuboid-like structure stabilized by salt bridges between hydrophilic residues in transmembrane helices. Phosphatidylcholine binding was observed in a hydrophobic cleft on the surface exposed to the outer leaflet of the plasma membrane. Six charged residues placed from top to bottom inside the molecule were essential for scrambling phospholipids in inward and outward directions, apparently providing a pathway for their translocation. A tryptophan residue was present between the head group of phosphatidylcholine and the extracellular end of the path. Its mutation to alanine made the Xkr8–Basigin complex constitutively active, indicating that it plays a vital role in regulating its scramblase activity. The structure of Xkr8–Basigin provides insights into the molecular mechanisms underlying phospholipid scrambling.

## Abstract (web summary)

Cryo-EM and X-ray crystal structures reveal the architecture of the human Xkr8–Basigin complex, providing insights into the molecular mechanism of phospholipid scrambling.

## Main

The lipid bilayer in eukaryote plasma membranes comprises asymmetrically distributed phospholipids^1^. Phosphatidylserine (PtdSer) and phosphatidylethanolamine (PtdEtn) localize to the inner leaflet of the plasma membrane, and phosphatidylcholine (PtdCho) to the outer leaflet. This asymmetrical distribution is disrupted in various biological processes, and PtdSer exposed to the cell surface activates enzymes or functions as a signal^2–4^. Activated platelets expose PtdSer as a scaffold for clotting enzymes, while PtdSer exposed on apoptotic cells functions as an ‘eat me’ signal for macrophages.

ATP-dependent flippases (P4-ATPases) translocate PtdSer and PtdEtn from the outer to inner leaflet^5^ and maintain their asymmetrical distribution. A high concentration of Ca^2+^ in activated platelets or caspase in apoptotic cells inactivates flippases^5,6^; however, this inactivation alone is insufficient for the swift exposure of PtdSer because it takes days for phospholipids carrying a hydrophilic head group to travel the hydrophobic lipid bilayer^7^. To expedite the exposure of PtdSer, cells carry scramblases that translocate phospholipids bidirectionally in an energy-independent manner^2,5^. We previously identified two membrane proteins (TMEM16F and Xkr8) as scramblases^8,9^ that are activated by distinct mechanisms. TMEM16F and several other TMEM16 family members, including their fugal homologs, function as Ca^2+^-dependent scramblases^8,10–14^. Xkr8 is a member of the XK family^9^ and forms a heterodimer with Basigin (BSG) or Neuroplastin (NPTN)^15^. It is activated by kinase or caspase to scramble phospholipids^9,16^. Xkr8 is responsible for exposing PtdSer in apoptotic cells, and its deficiency causes systemic lupus erythematosus–type autoimmune disease and male infertility^17,18^.

The tertiary structures of P4-ATPases and TMEM16F family members were previously elucidated^11,12,19–22^, and various models, such as the ‘stepping stone model’, ‘credit card model’, ‘out-of-the-groove model’ and ‘membrane distortion model’, have been proposed^23–27^. We herein report the structure of the human Xkr8–BSG heterodimer with a novel fold. It carries 22 hydrophilic amino acids in the transmembrane region of the molecule. A set of hydrophilic residues was necessary for the stability of the complex, while the other six charged residues were essential for its scrambling activity. The molecule had a hydrophobic cleft occupied by PtdCho on an upper surface. The head group of PtdCho coordinated with a tryptophan residue at the extracellular end of a transmembrane helix. Reductions in the hydrophobicity of the cleft made the molecule inactive, while the replacement of tryptophan by alanine made the molecule constitutively active. These results provide insights into the phospholipid scrambling mechanism.

## Results

### Purification of the hXkr8–hBSGΔ–Fab complex

**Extended Data Fig. 1:**
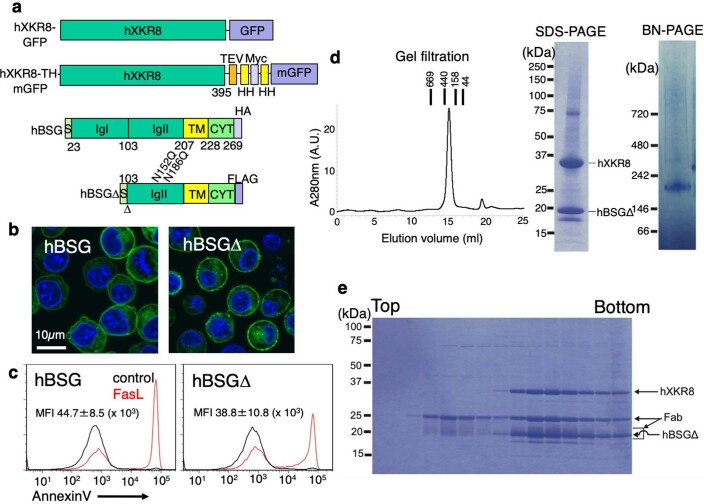
Purification of the hXkr8-hBSGΔ-Fab complex. **a**, Structures of hXkr8-GFP, hXkr8-TH-mGFP, hBSG, and hBSGΔ. mGFP, monomeric EGFP; TEV, TEV cleavage site; HH, Histidine tag (8 His); Myc, Myc-tag; S, Signal sequence; Ig, Immunoglobulin (Ig) domain; TM transmembrane region; CYT, cytoplasmic region. In hBSGΔ, the first Ig domain (IgI) was deleted, and two N-glycosylation sites (Asn) were mutated to glutamine. **b**,**c**, *BSG*^*-/-*^ *NPTN*^*-/-*^ W3 cells were transformed by hXkr8-GFP with hBSG or hBSGΔ and observed by confocal microscopy (**b**). The transformants were treated with FasL, and the Annexin V-staining profile in the PI-negative population was analyzed by flow cytometry (**c**). **d**, The purified hXkr8-hBSG complex was analyzed by gel filtration (7.5 μg protein), SDS-PAGE (6 μg), or BN-PAGE (3 μg). The molecular weight of standard proteins is shown in kDa. **e**, Purified hXkr8-hBSGΔ was incubated with Fab18 and subjected to GraDeR^29^. An aliquot of each fraction was analyzed by SDS-PAGE. hXkr8, hBSGΔ, and Fab (heavy and light chains) are indicated by arrows. Source data for **b, d**, and **e** are available online. Source data

The amino acid sequence of Xkr8 is well conserved in vertebrates (Supplementary Fig. 1). Xkr8 requires BSG or NPTN to localize to the plasma membrane^15^. We initially screened GFP-tagged Xkr8 orthologues for their expression and stability using fluorescence-detection size-exclusion chromatography and found that human Xkr8 (hXkr8) and hBSG were relatively stable and abundantly expressed. BSG carries two immunoglobulin domains, one of which is dispensable for its chaperone-like activity^15^. We removed the first immunoglobulin and modified *N*-glycosylation sites (hBSGΔ) (Extended Data Fig. 1a). hBSGΔ supported the translocation of hXkr8 to the plasma membrane in *NPTN*^*−/−*^*BSG*^*−/−*^W3 (DKO)^15^ cells (Extended Data Fig. 1b), and transformed cells retained the ability to scramble phospholipids during apoptosis (Extended Data Fig. 1c).

**Fig. 1:**
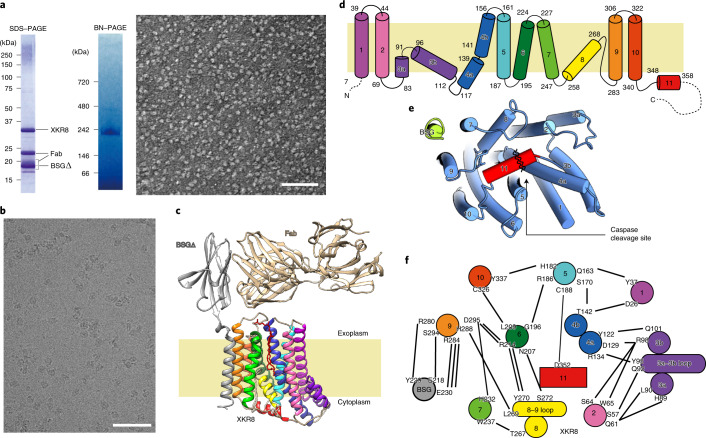
Structure of the hXkr8–hBSGΔ complex. **a**, Purification of the hXkr8–hBSGΔ–Fab18 complex. The purified hXkr8–hBSGΔ–Fab18 complex was analyzed by SDS–PAGE (3 μg) or blue native (BN)–PAGE (5 μg), or observed under an electron microscope (HITACHI H7650) after negative staining. Bar, 100 nm. **b**, A representative cryo-EM image. Bar, 50 nm. **c**, The structure of the hXkr8–hBSGΔ–Fab18 complex. The location of the membrane is estimated from the position of tryptophan (Extended Data Fig. 6). **d**, α-Helices of hXkr8 are numbered and schematically shown. **e**, A view of the hXkr8–hBSGΔ complex from its cytoplasmic side. **f**, Amino acids connected via hydrogen bonds or salt bridges in the hXkr8–hBSGΔ complex are shown. Source data for **a** are available online. Source data

**Extended Data Fig. 2:**
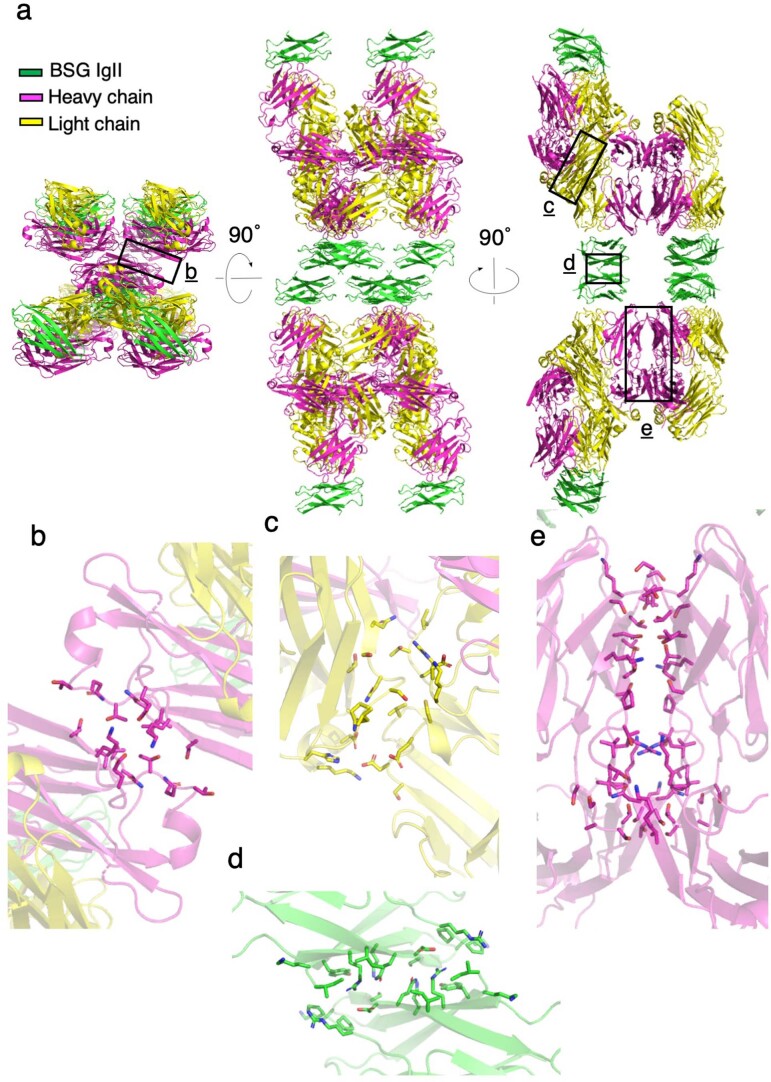
Crystal lattice contact of the Fab14-BSG IgII complex. **a**, Three views of Fab14-BSG IgII crystal packing. BSG IgII, the heavy chain, and light chain are shown in green, magenta, and yellow, respectively. **b-e**, Close-up views of the area boxed in **a**. Residues at the interface of the domains are represented by sticks.

hXkr8 was then fused to a histidine tag and EGFP and co-expressed with hBSGΔ in Sf9 cells using a baculovirus system. The hXkr8–hBSGΔ complex purified from the lauryl-maltose neopentyl glycol (LMNG)-solubilized membrane fraction was nearly homogeneous (Extended Data Fig. 1d). Since the Fab of a monoclonal antibody often enhanced the crystal formation of membrane proteins^28^, we prepared a Fab that preferentially recognized native over denatured hBSGΔ. We initially attempted to crystallize the hXkr8–hBSGΔ–Fab14 complex; however, it was not possible to visualize the electron density of the transmembrane regions in the crystals because these regions appeared to have been lost during crystallization. Nevertheless, X-ray crystallography of Fab14 and hBSGΔ–Fab14 revealed the structure of the extracellular region of the hBSGΔ–Fab14 complex at a high resolution of 2.51 Å (Table 1, Extended Data Fig. 2 and Supplementary Fig. 2). To elucidate the structures of the transmembrane regions of hXkr8 and hBSG, free LMNG was removed by GraDeR^29^ (Extended Data Fig. 1e), and nearly homogeneous monodispersed samples (Fig. 1a) were subjected to a cryo-EM single-particle analysis.

**Table 1:** Data collection and refinement statistics

	Fab14 (PDB 7D9Z)	hBSGext/Fab14 (PDB 7DAA)
Data collection		
Space group	*C*2	*C*222_1_
Cell dimensions		
*a*, *b*, *c* (Å)	163.45, 53.05, 54.31	117.35, 247.35, 522.25
*α*, *β*, *γ* (°)	90, 105.05, 90	90, 90, 90
Resolution (Å)	78.924–1.123 (1.194–1.123)^a^	123.676–2.509 (2.706–2.509)
*R*_merge_ (all I^+^ and I^−^)	0.048 (0.653)	0.187 (1.569)
*R*_merge_ (within I^+^/I^−^)	0.045 (0.659)	0.178 (1.421)
*I* / σ*I*	13.5 (1.4)	7.3 (1.3)
Completeness (spherical) (%)	81.6 (24.3)	68.4 (10.8)
Completeness (ellipsoidal) (%)	87.0 (33.7)	86.3 (45.7)
Redundancy	4.8 (3.1)	5.1 (7.2)
Refinement		
Resolution (Å)	36.40–1.12 (1.13–1.12)	123.68–2.51 (2.64–2.51)
No. reflections	139,266 (526)	18,231 (204)
*R*_work_ / *R*_free_	0.121 (0.256)/0.150 (0.333)	0.228 (0.344)/0.283 (0.551)
No. atoms		
Protein	3,418	3,847
Ligand/ion	37/0	0/11
Water	695	0
*B* factors		
Protein	19.78	38.92
Ligand/ion	21.50/−	–/118.45
Water	33.76	–
R.m.s. deviations		
Bond lengths (Å)	0.010	0.003
Bond angles (°)	1.178	0.565

The number of crystals for each structure is one. R.m.s., root mean square. ^a^Values in parentheses are for the highest-resolution shell.

### Structure of the hXkr8–hBSG complex

**Extended Data Fig. 3:**
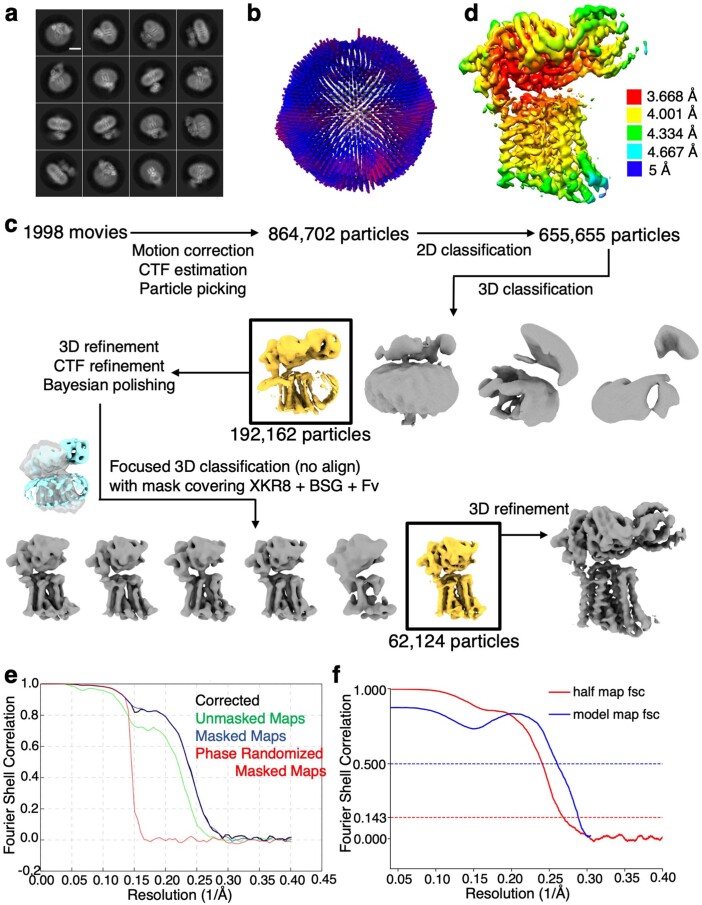
Cryo-EM characterization of the hXkr8-hBSGΔ-Fab complex. **a**, Representative 2D-class averages, bar, 5 nm. **b**, Angular distribution plot of particles included in the final 3D reconstitution. The length and color of cylinders indicate the number of views at each angular orientation. Red indicates more views. **c**, Data processing of the hXkr8-hBSGΔ-Fab complex. See Methods for the detailed procedure. **d**, Final reconstruction map colored by local resolution as calculated by RELION3.1. **e**, The Fourier shell correlation (FSC) plot between corrected (black), unmasked (green), masked (blue), and phase randomized (red) half-maps are shown. **f**, FSC plot for resolution estimations and model validation. The FSC curve between the EM half maps (red) and the FSC curve between the EM full map and atomic model (blue) are shown.

**Extended Data Fig. 4:**
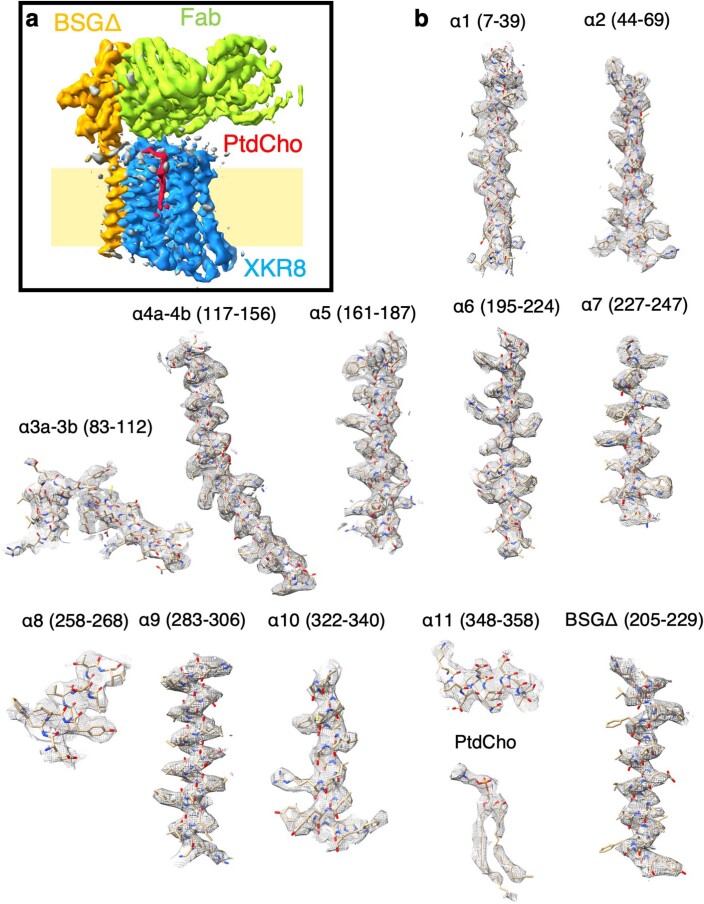
Cryo-EM density of the hXkr8-hBSGΔ-Fab complex. A cryo-EM map of the hXkr8-hBSGΔ-Fab complex. The location of the membrane is estimated from the surface hydrophobicity and position of tryptophan residues (Extended Data Fig. 6). **b**, Representative cryo-EM densities of 11 helices (α1-α11) of hXkr8, the transmembrane helix of hBSGΔ, and PtdCho are superimposed on the corresponding atomic model. Electron microscopy densities are shown in grey meshes, while the model is shown as sticks colored according to the atom type: C, tan; N, blue; O, red; and S, yellow.

**Extended Data Fig. 5:**
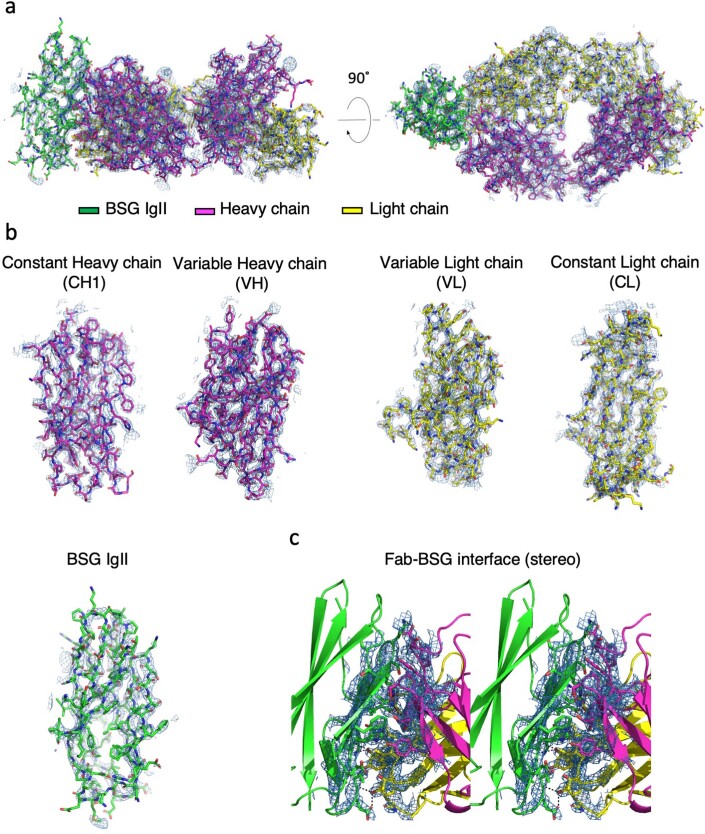
Electron density maps for the Fab14-BSG IgII complex. **a**, 2Fo-Fc electron density maps of Fab14 and BSG IgII with a 1.5σ contour level. BSG IgII, the heavy chain of Fab14, and the light chain of Fab14 are shown in green, magenta, and yellow, respectively. **b**, 2Fo-Fc electron density maps of the indicated domains with a 1.5σ contour level. Color code as above. **c**, A stereo view of the interface between Fab14 and BSG IgII. The blue mesh illustrates 2Fo-Fc electron density maps contoured at 1.5σ. Hydrogen bonds are shown as black dotted lines. Color code as above.

**Extended Data Fig. 6:**
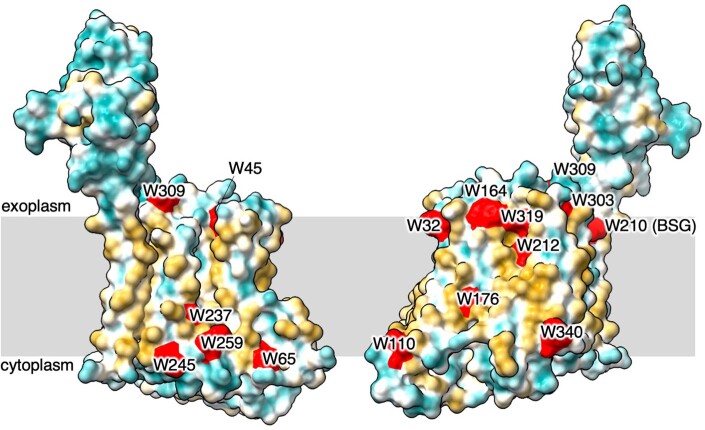
Surface hydrophobicity and tryptophan residues in the hXkr8-basigin complex. Features of the surface hydrophobicity of the hXkr8-basigin complex represented using the UCSF Chimera X program are shown in two views. Hydrophobic residues are in brown, polar residues in blue and neutral residues in white. Tryptophan residues in hXkr8 and the transmembrane region of basigin are shown in red.

Despite the relatively small size of the hXkr8–hBSGΔ–Fab18 complex (110 kDa), electron micrographs revealed the structure of the hXkr8–hBSGΔ–Fab18 complex at a resolution of 3.8 Å, measured using a Fourier shell correlation (FSC) cut-off of 0.143 (Fig. 1b, Table 2 and Extended Data Fig. 3). Based on the density map obtained by cryo-EM (Extended Data Fig. 4) and the X-ray structure of hBSGΔ–Fab (Extended Data Fig. 5), we elucidated the structure of the hXkr8–hBSGΔ–Fab complex. It consisted of hXkr8, hBSGΔ and Fab at a 1:1:1 ratio (Fig. 1c). Based on the position of tryptophan residues near the end of the helices^30,31^ and surface hydrophobicity (Extended Data Fig. 6), we determined the location of the membrane. hXkr8 comprised eight transmembrane helices (α1, α2, α4–7, α9 and α10), two helices traveling halfway to the membrane (α3 and α8) and one cytoplasmic helix (α11) (Fig. 1d). α11 contained a caspase 3-recognition sequence; interacted with α2, α4, α5 and α6 (Fig. 1e); and appeared to stabilize the disposition of these helices at the cytoplasmic side. The transmembrane region of hXkr8–hBSGΔ had a rectangular cuboid-like structure with a slight spread to the cytoplasmic face (Fig. 1c). Proteins with similar structures were not detected in a three-dimensional (3D) homology search (PDBeFold)^32^.

**Table 2:** Cryo-EM data collection, refinement and validation statistics

	hXkr8–hBSGΔ–Fab18 complex (EMD-30636) (PDB 7DCE)
**Data collection and processing**	
Magnification	105,000
Voltage (kV)	300
Electron exposure (e^−^/Å^2^)	48
Defocus range (μm)	0.8~1.8
Pixel size (Å)	0.83
Symmetry imposed	*C*1
Initial particle images (no.)	864,702
Final particle images (no.)	62,124
Map resolution (Å)	3.8
FSC threshold	0.143
Map resolution range (Å)	3.67–6.51
**Refinement**	
Initial model used	De novo structure
Map sharpening *B* factor (Å^2^)	−120
Model composition	
Nonhydrogen atoms	7,102
Protein residues	912
Ligands	1 (DLP)
*B* factors (Å^2^)	
Protein	84.16
Ligand	66.09
R.m.s. deviations	
Bond lengths (Å)	0.009
Bond angles (°)	1.203
Validation	
MolProbity score	2.45
Clashscore	11.63
Poor rotamers (%)	3.27
Ramachandran plot	
Favored (%)	92.34
Allowed (%)	7.44
Disallowed (%)	0.22

### Interaction between hXkr8 and BSG

**Fig. 2:**
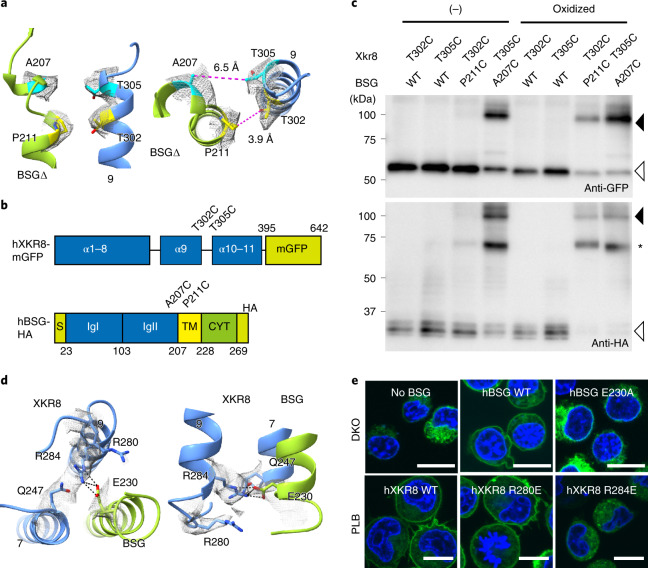
Interaction between hXkr8 and hBSGΔ. **a**, Side (left) and top (right) views of hXkr8–hBSGΔ near the extracellular surface with local density (gray mesh) from the cryo-EM map. **b**, Crosslinking of cysteine-substituted hXkr8 and hBSG. The residues mutated to cysteine (T302 and T305 in hXkr8, and A207 and P211 in hBSG) are indicated in their schematic structure. **c**, Wild-type (WT) and indicated mutant hXkr8-mGFP and hBSG-HA were expressed in HEK293T cells. Crude membrane fractions were treated or not with copper phenanthroline for oxidization, and subjected to western blotting for GFP (upper panel) or HA (lower panel). Black arrowheads, crosslinked protein; white arrowheads, monomer; *, BSG dimer^47^. **d**, The cytoplasmic (left) and side (right) views of the interaction between hXkr8 and hBSGΔ near the cytoplasm, with local density from the cryo-EM map shown as gray mesh. R284 of Xkr8 is connected with E230 of hBSG via hydrogen bonds. **e**, DKO cells expressing hXkr8-GFP with the WT or E230A hBSG, or PLB cells expressing EGFP-tagged WT, R280E or R284E hXkr8, were observed for EGFP and Hoechst 33342. Scale bar, 10 μm. Source data for **c** and **e** are available online. Source data

The hXkr8–hBSG structure and an analysis of hydrogen bonds and salt bridges indicated that α9 of hXkr8 was arranged near the transmembrane region of hBSGΔ (Fig. 1e,f). A207 and P211 of hBSGΔ were arranged toward T305 and T302 of hXkr8 at a distance of approximately 0.7 and 0.4 nm, respectively (Fig. 2a). These residues were individually mutated to cysteine, tagged with GFP (for hXkr8) or hemagglutinin (HA) (for BSG) (Fig. 2b) and expressed in HEK293T cells. Western blotting under nonreducing conditions showed a 100-kDa band with anti-GFP and anti-HA when hXkr8-T305C was co-expressed with hBSG-A207C (Fig. 2c). When the membrane fraction was treated with the oxidant before SDS–polyacrylamide gel electrophoresis (SDS–PAGE), P211C of hBSGΔ and T302C of hXkr8 were also crosslinked (Fig. 2c), confirming the proximity of these residues. At the cytoplasmic side of the hXkr8–hBSGΔ complex, E230 of hBSGΔ was close to Q247, R280 and R284 of hXkr8 (Fig. 2d). The E230A mutant of hBSG did not support the localization of hXkr8-GFP to the plasma membrane (Fig. 2e). The R284E mutant of hXkr8 did not localize to the plasma membrane in PLB cells expressing intact hBSG (Fig. 2e), indicating that the interaction between E230 of BSG and R284 of hXkr8 was indispensable for the chaperone activity of BSG.

### A hydrophilic pathway inside the molecule essential for scrambling phospholipids

**Fig. 3:**
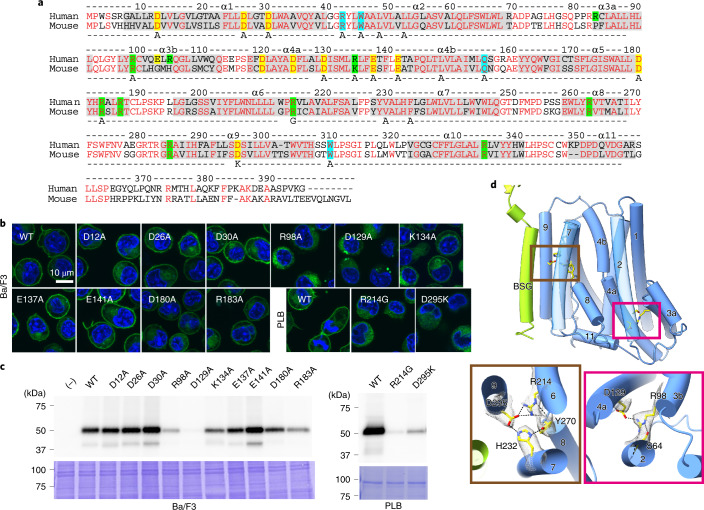
Charged residues in transmembrane helices of hXkr8. **a**, The sequences of hXkr8 (UniProt: Q9H6D3) and mXkr8 (UniProt: Q8C0T0) were analyzed using the MUSCLE Program (EMBL-EBI). Helices are shadowed and numbered. Conserved residues are in red. Negatively (Glu and Asp) and positively charged residues (Lys and Arg) in the lipid layer are highlighted in yellow and green, respectively. Residues (R42, Q155 and W309) that coordinate with the head group of PtdCho are highlighted in light blue. The amino acid residues mutated in Figs. 3–6 are indicated in the bottom line. **b**,**c**, GFP-tagged WT and indicated mutant mXkr8 (D12A, D26A, D30A, R98A, D129A, K134A, E137A, E141A, D180A and R183A) or hXkr8 (R214G and D295K) were stably expressed in mouse Ba/F3 or human PLB985, respectively, and observed under a fluorescent microscope (**b**). In **c**, whole-cell lysates (Ba/F3) or the light membrane fraction (PLB) were analyzed by western blotting with anti-GFP or stained by CBB. **d**, Stabilization of transmembrane α-helices by salt bridges. The regions at which helices α6–α9 and helices α4a and α3b interact with each other are expanded with local density from the cryo-EM map (gray mesh). Source data for **b** and **c** are available online. CBB, Coomassie brilliant blue. Source data

**Extended Data Fig. 7:**
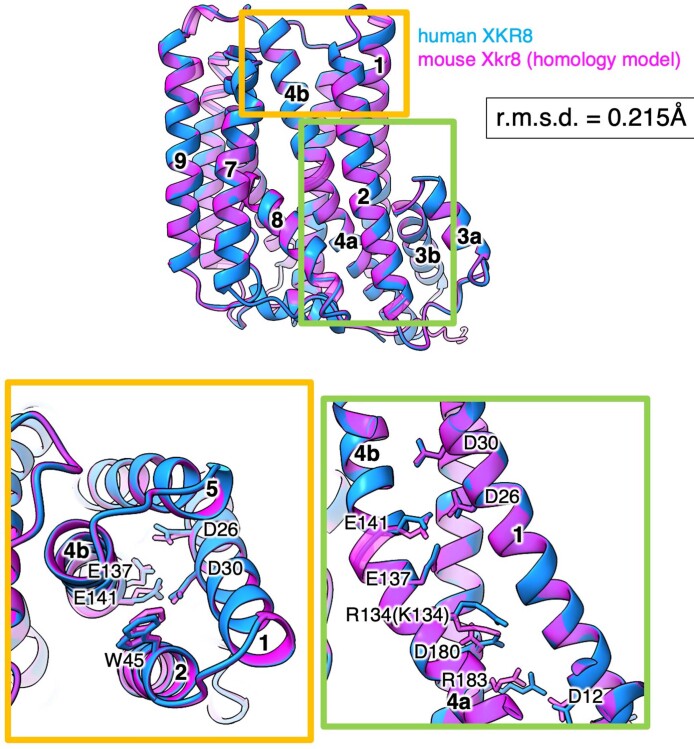
Homology model of the mXkr8 structure. The mXkr8 homology model was predicted with MODELLER^33^ using the hXkr8 structure (PDB:7DCE) as a template. It was superimposed on the hXkr8 structure with a root mean square deviation (r.m.s.d.) of 0.215 Å. The area containing the residues involved in scrambling phospholipids at the extracellular side (orange box) and those in the middle of the membrane (green box) are enlarged in the lower panels.

The amino acid sequence of mouse (m)Xkr8 has 68.9% identity with hXkr8 (272 of 395 amino acids) (Fig. 3a). Using the *Modeller* program^33^, the homology model of the mXkr8 structure was predicted, and the two structures were superimposed with a root mean square deviation of 0.215 Å, suggesting that the structure of mXkr8 was essentially identical to hXkr8 (Extended Data Fig. 7). An interesting feature of hXkr8 was the presence of 22 charged residues (Asp, Glu, Lys and Arg) in the α-helices at the lipid layer (Fig. 3a). Sixteen of these residues were well conserved among vertebrates (Supplementary Fig. 1). To examine the role of these charged residues, we selected 12 residues of mXkr8 or hXkr8 (D12, D26, D30, R98, D129, K134, E137, E141, D180, D295, R183 and R214) that were internally oriented (Protein Data Bank (PDB) 7DCE). R214 and D295 were mutated to glycine or lysine, respectively, similar to XK in human patients with McLeod Syndrome^34,35^. The other ten residues were mutated to Ala. The mutants were tagged by GFP at the C terminus and stably expressed in PLB985 or Ba/F3 cells. As shown in Fig. 3b, four mutants (R98A, D129A, R214G and D295K) were not localized at the plasma membrane. A western blot analysis showed that the expression of these mutant proteins was markedly reduced compared with the wild-type Xkr8 (Fig. 3c). The structure of hXkr8 indicated two clusters of hydrophilic amino acids (one group with R214, H232, Y270 and D295; and another with S64, R98 and D129) in the transmembrane region of the complex (Fig. 3d). R214 on α6 and D295 on α9 formed a salt bridge, while D129 in α4a formed a salt bridge with R98 on α3b. The reduced expression of the mutant proteins (R98A, D129A, D295K and R214G) indicated that the interaction between the hydrophilic residues in the transmembrane region plays an essential role in stabilizing the Xkr8–BSG complex.

**Fig. 4:**
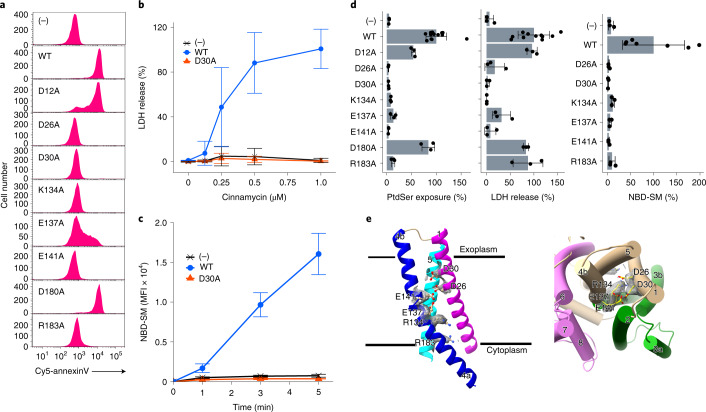
Requirement of charged residues in the membrane region of mXkr8 to scramble phospholipids. **a**, Ba/F3 (−) and its transformants expressing the WT or indicated mutant mXkr8 were stained with Cy5-annexin V and analyzed by flow cytometry. **b**, Ba/F3 (−) and its transformants expressing WT or D30A mutant mXkr8 were incubated at 4 °C for 15 min with the indicated concentrations of cinnamycin and the released LDH was quantified. Experiments were performed three times. The data, expressed as a percentage of that released with 1% Triton-X100, are presented as mean values ± s.d. **c**, Ba/F3 (−) and its transformants expressing WT or D30A mutant mXkr8 were incubated at 20 °C with NBD-SM for the indicated periods. Incorporated NBD-SM was analyzed by flow cytometry, and its mean fluorescence intensity (MFI) was determined. Experiments were performed three times. Data are presented as mean values ± s.d. **d**, The scramblase activity of Ba/F3 transformants expressing WT or indicated mutant mXkr8 was assayed for the exposure of PtdSer (at 4 °C for 15 min), sensitivity to cinnamycin (0.5 μM)-induced LDH release (at 4 °C for 15 min) and the incorporation of NBD-SM (at 20 °C for 5 min). Experiments were performed at least three times. Relative activities to that of WT are presented as mean values ± s.d. **e**, Close-up side and top views of charged amino acids (Glu, Asp and Arg) in the α1, α4 and α5 helices. The side chains of charged amino acids are represented in a colored ball and stick model with the cryo-EM map superimposed (gray mesh). Source data for **b**–**d** are available online. Source data

**Extended Data Fig. 8:**
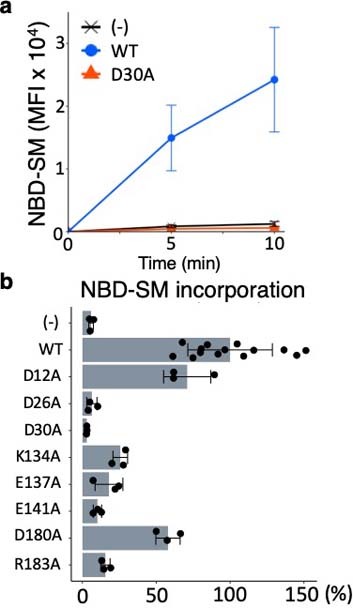
Charged residues for the internalization of NBD-SM. Ba/F3 cells were transformed with wild-type or indicated mXkr8 mutants. Parental Ba/F3 and its transformants were incubated at 4 °C with NBD-SM for the indicated periods (**a**) or 5 min (**b**), treated with lipid-free BSA, and analyzed by flow cytometry. In **a**, incorporated NBD-SM was analyzed by flow cytometry. Mean Fluorescence Intensity was regarded as inward scrambling activity. Experiments were performed three times. Data are presented as mean values + S.D. In **b**, the scrambling activity of each mutant was normalized by its expression level estimated by GFP fluorescence. Experiments were performed three times. The data, relative activities to that of the wild-type, are presented as mean values + S.D. Source data for **a** and **b** are available online. Source data

The eight other mutants (D12A, D26A, D30A, K134A, E137A, E141A, D180A and R183A) were expressed as efficiently as wild-type mXkr8 and localized at plasma membranes (Fig. 3b,c). mXkr8-mediated lipid scrambling was activated in mouse Ba/F3 cells by phosphorylation at the C-terminal tail region and PtdSer exposure occurred at 4 °C, a temperature at which P4-ATPase activity should be strongly reduced^16^. We used this system to examine the scrambling activities of mXkr8 mutants. As shown in Fig. 4a, Ba/F3 cell transformants expressing wild-type or D12A or D180A mutant mXkr8 exposed PtdSer. On the other hand, the exposure of PtdSer was markedly reduced in transformants expressing the D26A, D30A, K134A, E137A, E141A and R183A mutants, indicating that these residues play an indispensable role in scrambling PtdSer from the inner to outer leaflets of plasma membranes. Xkr8 nonspecifically scrambles various phospholipids^9^. Accordingly, the treatment with cinnamycin (Ro 09-0918), which kills cells exposing PtdEtn^36^, lysed Ba/F3 transformants expressing wild-type mXkr8, D12A, D180A and R183A at 4 °C for 15 min (Fig. 4b,d). However, its killing activity was reduced in cells expressing the D26A, D30A, K134A, E137A or E141A mutants. Xkr8 scrambles phospholipids from the inner to outer leaflets and from the outer to inner leaflets^9^. Accordingly, wild-type mXkr8 and the D12A and D180A mutants efficiently internalized NBD-SM ((7S)-4-hydroxy-7-[(1R,2E)-1-hydroxy-2-hexadecen-1-yl]-N,N,N-trimethyl-14-[(7-nitro-2,1,3-benzoxadiazol-4-yl)amino]-9-oxo-3,5-dioxa-8-aza-4-phosphatetradecan-1-aminium, 4-oxide inner salt) at 4 °C (Extended Data Fig. 8). In contrast, the mutations in D26, D30, K134, E137, E141 and R183 markedly reduced this activity. Since sphingomyelin does not activate flippases (ATP11A and ATP11C) at plasma membranes^37^, we assayed the incorporation of NBD-SM at 20 °C (Fig. 4c,d). Consistent with the results obtained at 4 °C, Ba/F3 cell transformants expressing wild-type Xkr8 efficiently incorporated NBD-SM. However, the six mutants exhibited negligible activity to internalize NBD-SM.

The structure of hXkr8 indicated that these six amino acids (D26, D30, R134, E137, E141 and R183) were arranged from top to bottom inside the molecule (Fig. 4e; PDB 7DCE) and appeared to provide a path for scrambling phospholipids.

### A hydrophobic cleft on the surface

**Fig. 5:**
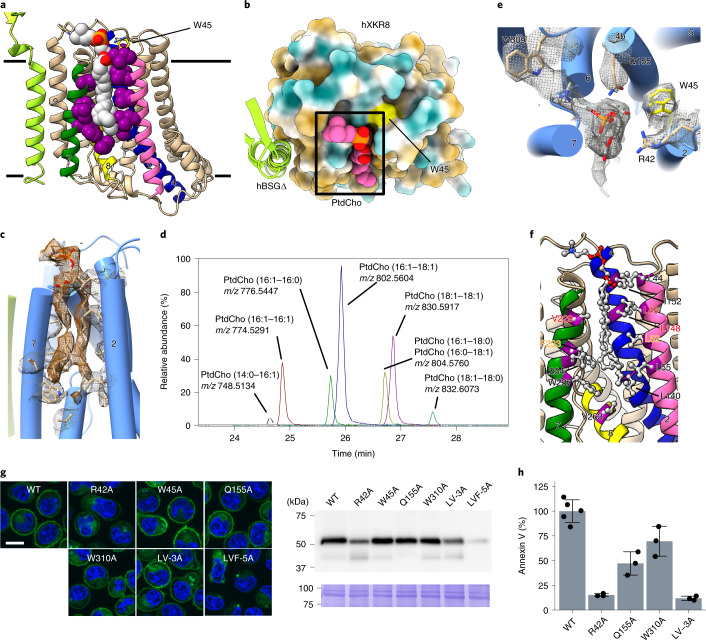
A lipid-binding cleft in hXkr8. **a**, Front view of the hXkr8–hBSGΔ complex. Hydrophobic residues surrounding PtdCho are in magenta spheres. PtdCho is in the silver sphere with the colored element. **b**, Top view of the complex. hXkr8 is in the colored surface view, brown, hydrophobic; blue, polar; white, neutral. PtdCho is colored in pink with oxygen in red and phosphorus in orange. **c**, A cryo-EM map of PtdCho (brown mesh) and surrounding residues (black mesh) is superimposed on the atomic model. **d**, LC–MS analysis of the lipid in the hXkr8–hBSGΔ complex in the negative ionization mode. The *m/z* values corresponding to the formate adduct ions of PtdCho species are indicated. **e**, A close-up top view of PtdCho with residues (W309, Q155, W45 and R42) that coordinate with the head group of PtdCho. The side chains of these amino acids and PtdCho are in a stick model with the superimposed cryo-EM map (gray mesh). **f**, Hydrophobic residues (Leu, Ile, Val, Phe and Trp) surrounding PtdCho are in a ball and stick structure. In the LV-3A mutant, L48, L148 and V229 were replaced by Ala (red). The LVF-5A mutant carries additional L52A and F233A mutations (orange). **g**, GFP-tagged WT and indicated mutant mXkr8 were expressed in mouse Ba/F3, and observed under a fluorescent microscope. On the right, whole-cell lysates were analyzed by western blotting with anti-GFP or stained by CBB. Scale bar, 10 μm. **h**, The scrambling activity of the indicated mutants was examined by incubating on ice for 15 min with Cy5-Annexin V. MFI obtained by each mutant was normalized by its expression level, and the percentage of that observed with WT mXkr8 was determined. Experiments were performed three times. The data are presented as mean values ± s.d. Source data for **g** and **h** are available online. Source data

**Extended Data Fig. 9:**
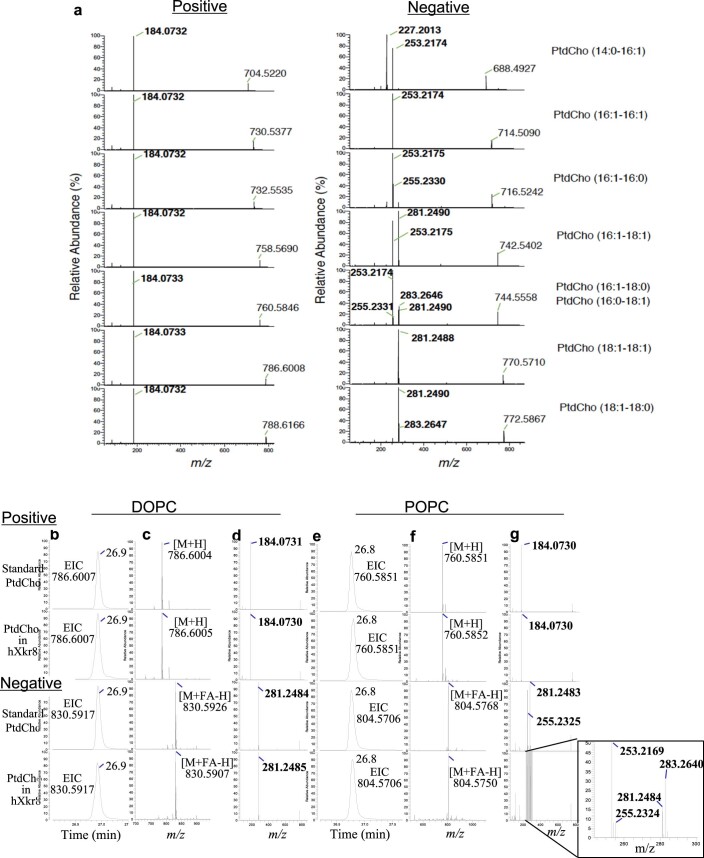
MS/MS spectra of PtdCho species from the hXkr8-hBSGΔ complex. **a**, The MS/MS spectra of PtdCho species extracted from the hXkr8-hBSGΔ complex (hXkr8) in positive (left) and negative (right) ionization modes. The numbers in bold represent the *m/z* values of fragment ions specific for each phospholipid and fatty acid class. In the positive ionization mode, a prominent peak at *m/z* 184.073 was assigned to phosphocholine^68^. In the negative ionization mode, the fatty acid composition of PtdCho was identified by the *m/z* values of fragment ions. **b-g**, Data-dependent LC-MS/MS was performed in positive and negative ionization modes with PtdCho standards, DOPC (18:1-18:1) (**b**-**d**) and POPC (16:0-18:1) (**e**-**g**), and PtdCho species from hXkr8. **b,e**, Extracted ion chromatograms (EICs) of the indicated *m/z* values corresponding to theoretical *m/z* of ionized DOPC and POPC. **c,f**, Precursor ion spectra at 26.9 and 26.8 min, respectively. **d,g**, Product ion spectra of the indicated *m/z* precursor ions. Numbers in bold represent *m/z* values of fragment ions specific for each phospholipid class and fatty acid class.

The hXkr8–hBSG complex contains a hydrophobic cleft facing the lipid bilayer in the upper middle part of the complex (Fig. 5a,b). The aperture was mainly surrounded by α2, α4, α6 and α7. Tilted α8, as well as the loop between α8 and α9, supported the crack from below. We detected an excess density in this cleft, which was consistent with PtdCho (Fig. 5c), and an LC–MS/MS analysis of the complex confirmed its presence (Fig. 5d and Extended Data Fig. 9). Bound phospholipids were expected to originate from Sf9 cells because extra phospholipids were not added during protein preparation, indicating the strong affinity of hXkr8 for phospholipids. The lipid fit well in the cleft (Fig. 5a,b). The contact area between hXkr8 and PtdCho, calculated by Protein Interfaces, Surfaces and Assemblies (PISA)^38^, was approximately 800 Å^2^, which was similar to that observed between the PtdCho transfer protein (PCTP) and PtdCho (PDB 1ln2)^39^. The head group of PtdCho fit well into the space comprising R42, W45, Q155 and W309 (Fig. 5e). The two acyl chains of PtdCho in the narrow cleft were linked by 12 hydrophobic amino acid residues (L44, L48, L52, L55, L140, L148, I152, V229, F233, L234, W237 and V263) (Fig. 5f). The arrangement of these amino acids with the lipid head group and acyl groups indicated that the aperture accepts other phospholipids.

We mutated three (LV-3A; L48, L148 and V229) or five (LVF-5A; L48, L52, L148, V229 and F233) hydrophobic amino acids in the middle of the aperture (Fig. 5f); R42, W45, Q155 and W310 (Fig. 5e) to alanine; C-terminally tagged them with GFP; and expressed them in Ba/F3 cells. All mutants, except for LVF-5A, were expressed at plasma membrane; however, their expression levels differed (Fig. 5g). W310A and Q155A supported the exposure of PtdSer substantially less than wild-type Xkr8, whereas R42A and LV-3A lost the ability to expose PtdSer (Fig. 5h). These results suggest that in addition to stabilizing the complex, the hydrophobic cleft occupied by PtdCho contributed to scrambling phospholipids.

### Constitutively active mutation

**Fig. 6:**
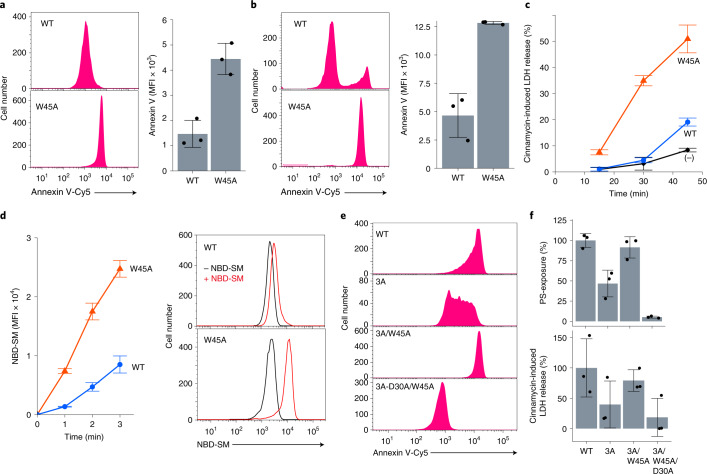
Regulation of phospholipid scrambling by a tryptophan at the extracellular end of the hydrophilic pathway. **a**,**b**, Effects of the W45A mutation on the exposure of PtdSer. Ba/F3 transformants expressing WT or W45A mutant mXkr8-GFP were subjected to Annexin V binding at 4 °C for 1 min (**a**) or at 20 °C for 15 min (**b**). Experiments were performed three times. The MFI data are presented as mean values ± s.d. **c**, Effects of the W45A mutation on the exposure of PtdEtn. Parental Ba/F3 (−) and its transformants expressing WT or W45A mXkr8-GFP were treated with 0.0625 µM cinnamycin at 37 °C for the indicated period. LDH released into the buffer was quantified. Experiments were performed three times. Data are presented as mean values ± s.d. **d**, Effects of the W45A mutation on the incorporation of NBD-SM. Ba/F3 transformants expressing WT or W45A were incubated at 20 °C for the indicated period, and analyzed by flow cytometry. Experiments were performed three times. The MFI data are presented as mean values ± s.d. The right panels show representative fluorescence-activated cell sorting (FACS) profiles before and after the 1-min incubation with NBD-SM. **e**,**f**, Phosphorylation-independent scrambling by the W45A mutant mXkr8. Ba/F3 cells were transformed with the WT, 3A (T356A/S361A/T375A), 3A-W45A or 3A-W45A/D30A mutant of mXkr8-GFP; incubated with Cy5-Annexin V; and analyzed by flow cytometry (**e**). In **f**, scramblase activity in Ba/F3 transformants expressing WT or indicated mutant mXkr8 was assessed at 4 °C for the exposure of PtdSer (for 15 min) and sensitivity to cinnamycin (0.5 μM)-induced LDH release (for 15 min). Experiments were performed three times. Relative activities to that of the WT are presented as mean values ± s.d. Source data for **a**–**d** and **f** are available online. Source data

The W45A mutant was expressed as efficiently as wild-type mXkr8, and localized to the plasma membrane of Ba/F3 cells (Fig. 5g). When we examined the exposure of PtdSer at 4 °C, the W45A transformants exposed PtdSer more abundantly and uniformly than wild-type mXkr8 (Fig. 6a), suggesting that scrambling activity was enhanced by the W45A mutation. The Annexin V-binding assay was then performed at room temperature to examine whether enhanced scramblase activity overcomes the flippase activity that translocates PtdSer from the outer leaflet to the inner leaflet. As shown in Fig. 6b, a small cell population expressing wild-type mXkr8 bound Annexin V, whereas all the population of W45A transformants firmly bound it (Fig. 6b). Furthermore, a low concentration of cinnamycin efficiently lysed W45A-expressing cells at 37 °C (Fig. 6c), indicating W45A mutant Xkr8 scrambled PtdEtn to the cell surface against flippase activity to transport PtdEtn. The ability of W45A mutant mXkr8 to internalize NBD-SM at 20 °C was approximately threefold stronger than that of wild-type mXkr8 (Fig. 6d), confirming the enhanced scrambling activity of the W45A mutant.

mXkr8 is activated by phosphorylation^16^, and the mutation of three phosphorylation sites in its C-terminal tail region (3A; T356A/S361A/T375A) markedly reduced its scrambling activity, which is consistent with previous findings^16^ (Fig. 6e,f). On the other hand, the transformants expressing the 3A-W45A mutant intensely exposed PtdSer, indicating that the W45A mutation provided mXkr8 constitutively active or phosphorylation-independent scramblase. Similarly, the 3A mutation reduced sensitivity to cinnamycin, and the W45A mutation rescued this effect (Fig. 6f). The exposure of PtdSer and sensitivity to cinnamycin caused by the W45A mutation were entirely blocked by mutating D30 to alanine (3A-D30A/W45A) (Fig. 6e,f). These results indicate that W45 serves as an essential residue regulating the phospholipid-scrambling activity of Xkr8 at plasma membranes.

## Discussion

A critical step in the translocation of phospholipids between the outer and inner leaflets of lipid bilayers is the movement of their hydrophilic head group across the hydrophobic core, and scramblase may provide a hydrophilic path(s) to facilitate this process^23^. Regarding TMEM16F of Ca^2+^-dependent scramblases and its homologs, various models have been proposed for scrambling phospholipids^24–27^. The Xkr8–BSG complex has a cuboid-like structure with no similarities to TMEM16 family members or other proteins in the database. Salt bridges between hydrophilic amino acids connected several α-helices in the middle of the transmembrane segments (BSG with α9; among α6–α9; α3 with α4). The critical residues for the interactions between α6 and α9 (R214 and D295) and between α4a and α3b (D129 and R98) were well conserved among the XK family (Supplementary Fig. 3), suggesting that these members have a similar structure. In fact, Straub et al.^42^ recently reported the tertiary structure of rat Xkr9 with a similar fold as human Xkr8.

The amino acid sequence of Xkr8 is evolutionally well conserved among vertebrates, and a model predicted an almost identical structure of mXkr8 to that of hXkr8. Among ten members of the TMEM16 family, mouse lymphoma Ba/F3 cells only express TMEM16F as their plasma membrane Ca^2+^-dependent scramblase^40^. Ba/F3 cells lacking TMEM16F and Xkr8 genes do not expose PtdSer when stimulated with a Ca^2+^ ionophore or apoptosis inducer. We previously demonstrated that when this cell line was transformed by mXkr8, they exposed PtdSer at 4 °C, but not at room temperature or higher^16^. Although the scramblase activity of TMEM16F and Xkr8 is not markedly affected by temperature^16,40,41^, lowering the temperature inhibits P4-ATPase activity^37^. Since the kinase inhibitor blocks the exposure of PtdSer in Ba/F3 transformants expressing mXkr8, phosphorylation by intrinsic kinase(s) may activate mXkr8 in Ba/F3 cells^16^. Using this mouse system, we assayed the scramblase activity of mXkr8 mutants. mXkr8 mutants were stably expressed in Ba/F3 cells deficient in TMEM16F and Xkr8 (ref. ^16^), and scramblase activity for the exposure of PtdSer or PtdEtn was measured at 4 °C to inhibit flippase. The internalization of NBD-SM, which was not expected to be affected by the flippases^37^, was performed at a higher temperature. The replacement of a tryptophan residue (W45) located at the extracellular boundary to alanine made the molecule constitutively active or phosphorylation-independent, suggesting that the active form of the mXkr8–BSG complex has a similar structure to that elucidated in the present study. This may agree with the report that caspase cleavage does not induce a substantial conformational change in rat Xkr9 (ref. ^42^).

We detected a hydrophobic aperture occupied by a single PtdCho molecule on one surface of hXkr8 facing the outer leaflet of the lipid bilayer. The alanine mutations of three hydrophobic residues (L48, L148 and V229) surrounding the PtdCho blocked the phospholipid scrambling evoked by the mXkr8–BSG complex. The mutations of the residues (R42 and Q155) that coordinate to the head group of PtdCho also affected the scrambling activity. A series of eight charged residues were present in the helixes of the internal side of the molecule, and at least six of them were indispensable for scrambling phospholipids, thereby supporting their provision of a pathway for phospholipid scrambling. These mutations, except for R183A in the exposure of PtdEtn, similarly affected the outward (PtdSer and PtdEtn exposure) and inward translocation (the incorporation of NBD-SM) of phospholipids, indicating that different phospholipids use the same or overlapping paths for inward and outward movements in most cases.

**Extended Data Fig. 10:**
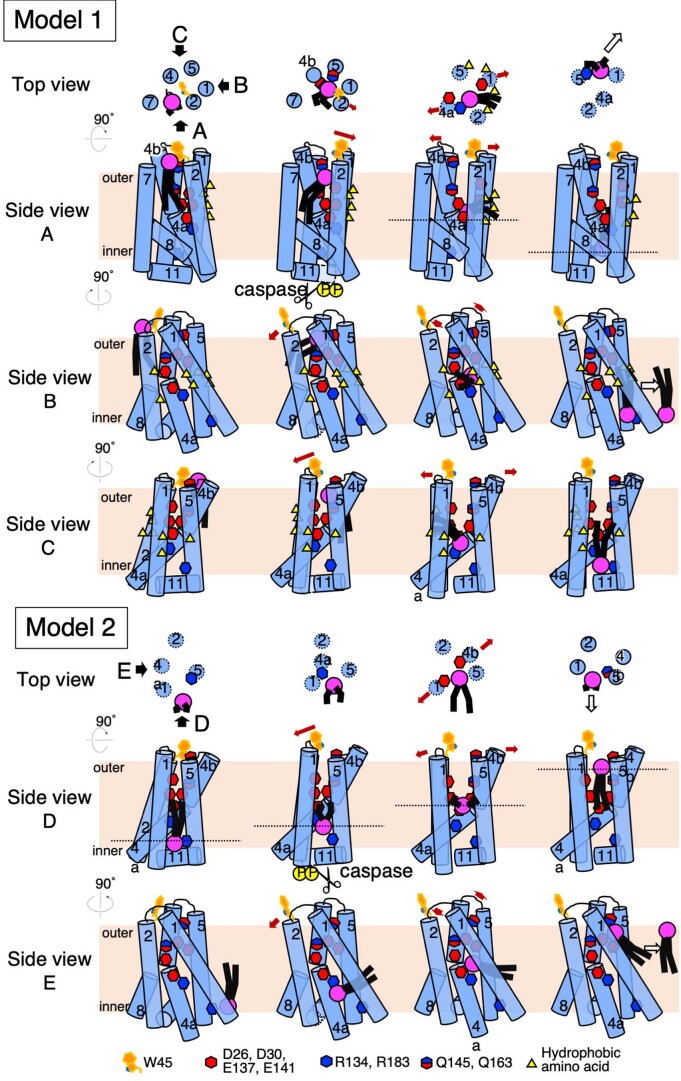
Two models for Xkr8-mediated phospholipid scrambling. In the first model, a phospholipid enters the hydrophobic cleft between α2 and α7 (Step 1). Caspase 3 cleaves Xkr8 in the middle of α11 or a putative kinase(s) phosphorylates residues at the C-terminal tail region, which causes the exterior bending of α2 and removes W45 from the path. It causes the opening of the upper space between α2 and α4b. The head group of the phospholipid enters the upper area of the track, and its interaction with Q163 and Y37 may open the hydrophilic path (Step 2). The contact of the phosphate group with acidic residues (D26, D30, E137, and E141) in the track generates the repulsion force and widens the pathway for the phospholipid to move. The hydrophobic residues (F23, L27, L31, V49, L50, and L53) between α1 and α2 facilitate the movement of acyl groups (Step 3). The phospholipid moves to the cytoplasmic site by interacting with positively charged residues (R134 and R183) and leaves the molecule between α1 and α5 (Step 4). In this model, the movement of phospholipids from the outer to inner leaflets opens the pathway. The second model is similar to the credit card model^23^. When Xkr8 is activated, the cytoplasmic space between α1 and α5 becomes wider, and the phospholipid head group is recruited into the space via positively charged residues (R134 and R183), leaving the acyl groups in the membrane area (Steps 1 and 2). The contact of the negatively charged head group with acidic residues (D26, D30, E137, and E141) broadens the upper space between α1 and α5 (Step 3), and the phospholipid leaves the molecule (Step 4). In this model, the hydrophobic cleft between α2 and α7 is not for phospholipid entry, and the PtdCho in the aperture stabilizes the molecule.

Phospholipid scrambling is a downhill reaction that does not require ATP and serves to destroy the asymmetrical distribution of phospholipids between two lipid layers^2^. Based on the structure of hXkr8–BSG and the results obtained from the mutational analysis of mXkr8–BSG, we propose the following working models for the activation mechanism of Xkr8 to scramble phospholipids (Extended Data Fig. 10). In the first model, phospholipids, such as PtdCho and sphingomyelin, in the outer layer of plasma membranes are recruited to the hydrophobic cleft of Xkr8–BSG. Caspase 3 activated during apoptosis cleaves α11 to remove the C-terminal tail region, or a putative kinase(s) phosphorylates residues at the C-terminal tail region^16^. The W45A mutant works as a constitutive-active form, suggesting that the caspase cleavage may cause the bending of W45. The phospholipid in the aperture then approaches through α2 and α4b to the scrambling path via an interaction with R158, Q163 and Q145. As proposed for the scrambling mechanism of TMEM16 and opsin^43–46^, once the phospholipid enters the trail, it is likely to affect or modify the pathway. The essential role of acidic residues (D26, D30, E137 and E141) for the scrambling activity suggests that the repulsion force generated between the negative charge of the phospholipid’s head group and acidic residues in the path may widen the track to allow the phospholipid to pass. The α-helixes (α1, α2, α4 and α5) composing the hydrophilic pathway carry many hydrophobic residues between the helixes which may allow acyl groups to move through the molecule. The mutation of K134 (corresponding to R134 in human Xkr8) and R183 prevented the scrambling, suggesting that phospholipids that travel to the cellular side are recruited by R134 and R183.

In another model (Model 2), the activation of the Xkr8–BSG complex by caspase may broaden the space between α1 and α5, and phospholipids scramble according to the ‘credit card’ model^23^ through this area by inserting their charged head group into the hydrophilic cleft, leaving the acyl group into the lipid layer. In this model, phospholipids do not use the hydrophobic cleft as the entry site, and PtdCho serves to stabilize the molecule. The acidic residues (D26, D30, E137 and E141) in the hydrophilic cleft are unlikely to be used as stepping stones for the head group of phospholipids, and they may be used to widen the path. Although we prefer the first model, further studies on the structures of different intermediate states carrying the phospholipids will be needed to prove this model. As described above, Xkr8 is a member of the XK family, which comprises nine members in humans (Supplementary Fig. 3). The tertiary structures of human Xkr8–BSG complex and rat Xkr9 (ref. ^42^) will provide a template for understanding the molecular mechanisms underlying phospholipid scrambling and structure–function analyses of the XK family.

## Methods

### Cell lines, recombinant proteins and materials

*Spodoptera frugiperda* 9 (Sf9) cells (ATCC CRL-1711) were cultured in Sf-900IIISFM (Gibco); PLB985 cells^9^, EL-4 cells (ATCC TIB-39) and *BSG*^*−/−*^ *NPTN*^*−/−*^ W3 cells^15^ in RPMI-10% FCS and 50 μM β-mercaptoethanol; *TMEM16F*^*−/−*^*Xkr8*^*−/−*^Ba/F3 cells (ref. ^16^) in RPMI-10% FCS and 45 units per ml of IL-3; and HEK293T cells in DMEM-10% FCS.

HRP-rabbit anti-GFP antibody (anti-GFP pAb-HRP-DirecT) and HRP-mouse anti-HA monoclonal antibody (Direct-Blot HRP anti-HA.11 Epitope Tag antibody) were from MBL and BioLegend, respectively. Biotin-conjugated Ro 09-0198 (ref. ^36^) was a gift from K. Nagao (Kyoto University). Cy5-Annexin V was from BioVision. 1,2-dioleoyl-sn-glycero-3-phosphocholine (DOPC) was from Avanti Polar Lipids. NBD-SM was from Cayman CHEMICAL. LMNG, glycol-diosgenin (GDN), octaethyleneglycol mono-*n*-dodecylether (C_12_E_8_) and fluorinated Fos-choline 8 (FC8) were from Anatrace. Cholesteryl hemisuccinate (CHS) and tobacco etch virus (TEV) protease were from Sigma-Aldrich.

### Complementary DNAs and expression plasmids

The DNA sequence for hXkr8 (UniProt: Q9H6D3) was custom-designed to enhance messenger RNA stability and translational efficiency and synthesized by Thermo Fisher Scientific. hXkr8 cDNA was introduced into pBAC-3bV, a derivative of pFastBac1 (Thermo Fisher Scientific), to express the protein tagged C-terminally with a TEV cleavage sequence, eight histidine residues, a c-Myc, eight histidine residues and monomeric EGFP (A206K) in this order^48^. This construct is hereafter referred to as hXkr8–TH–mGFP. cDNAs for hBSG, mBSG, mXkr8 and mXkr8 S/T-3A (T356A/S361A/T375A) mutant were described previously^15,16^. hBSG cDNA lacking the immunoglobulin-1 (Ig1) (amino acids 23–102) was produced by PCR, fused to a FLAG at the C terminus and introduced into pBAC-3bV. The DNA fragments for LV-3A (L48A, L148A, V229A) and LVF-5A (L48, L52, L148, V229, F233) of mXkr8 were custom-synthesized by Eurofins Genomics. Other mutants of hBSG, hXkr8 and mXkr8 were prepared by PCR using primers described in Supplementary Table 1. The resultant cDNAs were introduced into pMXs-Puro c-GFP^9^, pCX4-bsr c-HA^15^, pPEF-mEGFP-FLAG^16^ or pNEF-HA (pEF-BOS-EX^49^ carrying a neomycin resistance gene and HA tag). The authenticity of cDNAs was confirmed by sequencing.

### Purification of hXkr8–hBSGΔ complex

Baculovirus carrying hXkr8–TH–mGFP or hBSGΔ cDNA was produced using Bac-to-Bac Baculovirus Expression System (Thermo Fisher Scientific). Sf9 cells were co-infected with baculoviruses carrying hXkr8–TH–mGFP or hBSGΔ, and cultured for 60 h. Infected Sf9 cells were suspended in 20 mM HEPES-NaOH buffer (pH 7.0) containing 150 mM NaCl, 1 mM EDTA, 1 mM EGTA, 0.4 mM Pefabloc SC, 1 mM pAPMSF, 1 mM Tris(2-carboxyethyl)phosphine (TCEP) and protease inhibitors (cOmplete, EDTA-free, Roche Diagnostics). After adding 1.3 units per ml of benzonase (Merck), cells were disrupted on ice using an ultrasonic disrupter (Sonifier 450 Advanced; Branson) (0.5-s sonication at a level of 4 and 0.5-s cooling intervals for a total sonication period of 2.5 min). Unbroken cells and nuclei were removed by centrifugation at 6,000*g* for 25 min, and crude membrane fractions were collected by centrifugation at 99,650*g* for 1 h. Membranes were homogenized using a Dounce homogenizer in 20 mM HEPES-NaOH buffer (pH 7.0) containing 150 mM NaCl, 25% glycerol, 50 mM imidazole, 0.4 mM Pefabloc SC, 1 mM pAPMSF, 1 mM TCEP and protease inhibitors, and subjected to sonication (0.5-s flash with 0.5-s cooling; total sonication time of 30 s) at an output level of 3. LMNG and CHS were added to 1.0% and 0.1%, and incubated at 4 °C for 2 h. After centrifugation at 99,650*g* for 1 h, the lysates were loaded onto a HisTrap High Performance column (Cytiva) attached to an HPLC system (Prominence; Shimadzu), and washed with 60–80 mM imidazole in Buffer A (20 mM HEPES-NaOH buffer (pH 7.0), 0.01% LMNG, 0.001% CHS, 150 mM NaCl and 1 mM TCEP) containing 25% glycerol. The hXkr8–TH–mGFP/hBSGΔ complex was eluted with a linear 80–400 mM imidazole gradient in Buffer A containing 25% glycerol and treated at 4 °C with TEV protease for 12–18 h while dialyzing against Buffer A containing 10% glycerol and 1 mM EDTA. The sample was re-loaded over a HisTrap column equilibrated with 20 mM HEPES-NaOH buffer (pH 7.0), 0.005% LMNG, 0.0005% CHS, 150 mM NaCl, 10% glycerol and 1 mM TCEP, and the flowed-through fractions were concentrated approximately 100-fold by ultrafiltration using an Amicon Ultra-15 50K filter.

Analytical size-exclusion chromatography was performed at 4 °C with a Superose 6 Increase 10/300 GL column (Cytiva) attached to an HPLC system. The column was developed with TBS (20 mM Tris-HCl (pH 7.5) and 150 mM NaCl) containing 0.005% LMNG, 0.0005% CHS, 10% glycerol, 1 mM DTT, 10 µM pAPMSF and 100 µM Pefablock SC at a flow rate of 0.28 ml min^−1^. Proteins were detected using absorbance at 280 nm.

### Monoclonal antibodies against the hXkr8–hBSG complex

Rabbit monoclonal antibodies against hXkr8–hBSGΔ were established as described^50^. New Zealand White rabbits from KITAYAMA LABES were immunized with hXkr8–hBSGΔ using TiterMax (Sigma-Aldrich) or aluminum salts as adjuvants. IgG-positive B cells from peripheral mononuclear cells and splenocytes were cultured at 37 °C for 11 d at 2 cells per well together with 2.5 × 10^4^ Mitomycin C-treated murine EL-4 cells. Screening using the Octet Red96 System (ForteBio) identified two clones (XBA14 and XBA18) that recognized hXkr8–hBSGΔ. Their amino acid sequences differed at four positions at the variable region. However, their CDR3 domain had the same sequences, suggesting that they recognize the same epitope. DNA fragments for the variable regions of the heavy and light chains were inserted into pEF-BOS-based expression vectors carrying the constant region of human IgG heavy or light chain. The recombinant IgG (XBA14 and XBA18) was produced in FreeStyle 293-F cells (Thermo Fisher Scientific), and purified by Protein A-Sepharose (Cytiva) or MabSelect SuRe (Cytiva).

All animal studies were approved by the Animal Care and Use Committees of the Research Institute of Microbial Diseases, Osaka University and Chugai Pharmaceutical Co.

### Preparation of the hXkr8–hBSGΔ–Fab complex

To prepare the Fab fragment, IgG was digested at 35 °C for 2 h with Lys-C in Tris-HCl buffer (pH 8.0), and Fc fragments were removed using HiTrap SP HP (Cytiva) and MabSelect SuRe columns. Fab fragments (Fab14 and Fab18) were purified by gel filtration (Superdex 200 pg 16/600, Cytiva) in 20 mM HEPES-NaOH (pH 7.1) containing 100 mM NaCl. The hXkr8–hBSGΔ was then incubated with the Fab at 4 °C for 20–30 h in 16 mM Tris-HCl (pH 7.5), 4 mM HEPES-NaOH (pH 7.1), 140 mM NaCl and 8% glycerol, and subjected to gel filtration using Superose 6 Increase 10/300 GL column (Cytiva) equilibrated with TBS containing 10% glycerol. Fractions containing hXkr8–hBSGΔ–Fab were collected and concentrated by ultrafiltration using Vivaspin 2-100K (Cytiva).

In the cryo-EM analysis, detergents in the sample were removed by GraDeR as described^29^. The hXkr8–hBSGΔ was incubated with the Fab18 at 4 °C for 20–30 h in 20 mM HEPES-NaOH buffer (pH 7.0), 136 mM NaCl, 7% glycerol and 0.7 mM TCEP. Using a Gradient Master (BIO COMP), a linear gradient of glycerol (10% to 30%) with a reverse gradient of detergents (0.00225% to 0% for LNMG and 0.00075% to 0% for GDN) was established in 4 ml of 20 mM HEPES-NaOH buffer (pH 7.0) containing 100 mM NaCl, 1 mM EGTA, 1 mM TCEP, 10 µM *p*APMSF and 100 µM Pefabloc SC. Protein samples (0.1–0.2 ml) were loaded on the top of the gradient, and centrifuged at 200,614*g* for 15 h using a Beckman SW55Ti rotor. Gradients were fractionated using the Piston Gradient Fractionator (BIO COMP), and fractions carrying hXkr8–hBSGΔ–Fab18 were pooled and concentrated by ultrafiltration using a Vivaspin 2-100K. Glycerol was removed by repeating concentration and 30–40-fold dilution with the grid buffer (20 mM HEPES-NaOH (pH 7.0), 100 mM NaCl, 1 mM EGTA, 1 mM TCEP, 10 µM *p*APMSF and 100 µM Pefabloc SC) three times.

### Crystallization and structural analysis

For crystallization of Fab14, 100 nl of 7.1 mg ml^−1^ Fab14 was mixed with an equal volume of reservoir solution (0.1% n-Octyl-β-d-glucoside, 0.1 M sodium citrate tribasic dihydrate and 22% PEG3350 (Hampton)) and kept at 20 °C. Crystals were collected after 10 d; soaked in 20 mM HEPES-NaOH buffer (pH 7.1) containing 0.1% n-Octyl-β-d-glucoside, 0.1 M sodium citrate tribasic dihydrate, 22% PEG3350, 100 mM NaCl and 15% ethylene glycol; and flash-frozen in liquid nitrogen. Diffraction data were collected by helical scanning at BL32XU (SPring-8) on an EIGER X 9M detector (Dectris) and processed with autoPROC^51^, STRANISO^52^ and the CCP4 program^53^. The structure was elucidated by molecular replacement with Molrep^54^ using a rabbit antibody (PDB 4ma3) as a search model, and refined by Refmac5 (ref. ^55^), phenix.refine^56^ and *Coot*^57^. The final model for Fab14 included all amino acid residues, except for residues from positions 127 to 132 of the heavy chain.

The hXkr8–hBSGΔ–Fab14 was crystallized by the sitting drop vapor diffusion method with MemGold system (Molecular Dimensions). hXkr8–hBSGΔ–Fab14 (8.1 mg ml^−1^) in a glass tube coated with a film of 90 µg of DOPC was supplemented with 225 µg of C_12_E_8_ (lipid detergent ratio of 1:2.5) and incubated overnight while mixing. The lipidated protein was mixed with an equal volume of 0.1 M Tris-HCl (pH 8.0) buffer containing 0.1 M sodium chloride, 0.1 M cadmium chloride hemi(pentahydrate) and 33% PEG400, and kept at 20 °C. Crystals were collected after 2 months and flash-frozen in liquid nitrogen. X-ray diffraction data were collected by helical scanning using KUMA and SHIKA programs at the BL32XU beamline at SPring-8 and processed as described above. The phase was identified by molecular replacement by Molrep^54^ using Fab14, and domain 2 of BSG (PDB 3B5H) was placed by the *Coot* program^57^. The model was refined by *Coot*^57^ and phenix.refine^56^ programs, resulting in the structure of Fab14 and the extracellular region of hBSGΔ. Other parts, including hXkr8 and the transmembrane region of hBSG, were not modeled because the electron density map did not visualize them. hXkr8 likely dissociated from hBSGΔ during the crystallization. The crystallographic and refinement statistics are summarized in Table 1. Molecular graphics were illustrated using PyMOL Molecular Graphics System, v.2.4.0. (Schrodinger)

### Electron microscopy

In negative-staining electron microscopy, the hXkr8–hBSGΔ–Fab complex (0.01 mg ml^−1^) was applied to freshly glow-discharged, carbon-coated 200-mesh copper grids (Nisshin EM), and briefly blotted with filter paper (Whatman no. 1). Samples were stained with 2% uranyl acetate, and subjected to transmission electron microscopy (H7650, HITACHI).

For cryo-EM, immediately before preparing grids, fluorinated FC8 was added at a final concentration of 0.075% to hXkr8–hBSGΔ–Fab complex (8 mg ml^−1^). Samples were applied to the glow-discharged Quantifoil holey carbon grid (Cu/Rh, R1.2/1.3, 200-mesh), blotted by Vitrobot (Thermo Fisher Scientific) for 4 s under 100% humidity at 6 °C with a blot force of +10 and plunge-frozen in liquid ethane. Movies were acquired by a Titan Krios (Thermo Fisher Scientific) equipped with the K3 summit direct electron detector (Gatan) operated at 300 kV and working under low-dose conditions (48 frames at 48 e^−^ /Å^2^).

Movies were subjected to beam-induced motion correction using RELION 3.1, and contrast transfer function parameters were estimated by CTFFIND4 (ref. ^58^). All of the following processes were performed using RELION 3.1. Particles were picked from 200 randomly selected micrographs using template-free Laplacian-of-Gaussian picking, and subjected to multiple rounds of reference-free two-dimensional (2D) classification. Good 2D classes were selected as templates and 2D template-based particle picking was performed. In total, 864,702 particles from 1,977 micrographs were auto-picked, extracted with down-sampling to a pixel size of 3.32 Å per pixel and subjected to 2D classification. Among 655,655 particles subjected to 3D classification, 192,162 particles were selected, re-extracted with a pixel size of 1.245 Å per pixel and subjected to 3D refinement. The 3D map and particle set yielded were subjected to per-particle defocus refinement, beam-tilt refinement, Bayesian polishing and 3D refinement. No-align focused 3D classification with a mask covering hXkr8–hBSGΔ and the Fv domain of Fab resulted in one class of map with 62,124 particles. Final 3D refinement and postprocessing yielded a map with an overall resolution of 3.8 Å, estimated by the gold-standard FSC = 0.143 criterion. The processing strategy is described in Extended Data Fig. 3.

### Model building and refinement

At a resolution of 3.8 Å, the cryo-EM density map had sufficient quality for the de novo model building of hXkr8 and the transmembrane helix of hBSG. A polyalanine helix model was initially built by Coot^57^, and amino acids were subsequently assigned based on the density of bulky side chains. The structure of the extracellular domain of hBSG and Fab, elucidated by the X-ray diffraction analysis, was fit to the map by rigid body refinement (phenix.real_space_refine)^59^. The model was then manually fit to the map using *Coot*^57^, and simulated annealing at 500 K using the program of phenix.real_space_refine. The first 6 amino acids and last 35 amino acids of hXkr8, and the last 35 amino acids of hBSG, were not modeled. Molecular graphics were illustrated using UCSF Chimera^60^ and ChimeraX^61^.

### Expression level of Xkr8

For the western blotting with the whole-cell lysates, cells were lysed on ice for 10 min in radioimmunoprecipitation assay (RIPA) buffer (50 mM HEPES-NaOH buffer (pH 8.0), 1% Nonidet P-40, 0.1% SDS, 0.5% sodium deoxycholate and 150 mM NaCl) containing protease inhibitors, and insoluble materials were removed by centrifugation. The lysates were incubated at room temperature for 30 min in SDS sample buffer (40 mM Tris-HCl buffer (pH 6.8), 2% SDS, 5% glycerol, 1% 2-mercaptoethanol and 0.01% bromophenol blue), and separated by 10–20% SDS–PAGE (Nacalai Tesque). Proteins were transferred to a PVDF membrane (Merck), blotted with 10,000-fold-diluted HRP-rabbit anti-GFP antibody (anti-GFP pAb-HRP-DirecT) and visualized using Immobilon Western Chemiluminescent HRP substrate (Merck).

For western blotting with the light membrane fractions, cells were homogenized with a Dounce homogenizer in 10 mM Tris-HCl buffer (pH 7.5) containing 1 mM *p*APMSF and mixed with an equal volume of 10 mM Tris-HCl buffer (pH 7.5) containing 0.5 M sucrose, 0.1 M KCl, 10 mM MgCl_2_, 1 mM EGTA and 1 mM *p*APMSF. After successive centrifugations at 800*g* for 10 min and at 8,000*g* for 10 min, membrane fractions were collected by centrifugation at 100,141*g* for 1 h. Crude membranes were suspended in 10 mM Tris-HCl buffer (pH 7.5) containing 50 mM KCl, 5 mM MgCl_2_, 0.5 mM EGTA, 1 mM *p*APMSF and 40% (w/v) sucrose, and were loaded between 17% and 50% (w/v) sucrose layers in centrifugation buffer. After centrifugation at 100,141*g* at 4 °C for 150 min, the light membrane fraction at the 17%/40% (w/v) sucrose interface was collected, diluted with centrifugation buffer and collected by centrifugation. The precipitates were homogenized in TBS containing 10% glycerol and protease inhibitors by passing through a non-dead-space 29 G needle (TERUMO). A 1/10 volume of 10% LMNG -1% CHS was added to the sample and rotated at 4 °C for 2 h. After removing insoluble materials by centrifugation, the supernatants were recovered as light membrane lysates.

### Crosslinking experiment

Crosslinking of Cys-substituted hXkr8 and hBSG was performed as described^62^. Briefly, HEK293T cells were transfected with the expression vectors for hXkr8 and hBSG, cultured for 2 d, suspended in TBS with protease inhibitors and disrupted by sonication for 30 s at an amplitude of 20 using an ultrasonic disrupter (Q55; Qsonica). After centrifugation at 6,000*g* for 25 min, the crude membrane fractions were collected by centrifugation at 99,731*g* for 1 h; homogenized in TBS-10% glycerol, 1 mM EGTA and protease inhibitors; and treated at room temperature for 5 min with 1 mM reduced glutathione. The membranes were incubated at room temperature for 10 min with copper phenanthroline (0.3 mM CuSO_4_ and 0.9 mM 1,10-phenanthroline). After adding 0.25 vol. of 200 mM Tris-HCl buffer (pH 6.8) containing 10% SDS, 25% glycerol, 25 mM N-ethylmaleimide and 0.05% bromophenol blue, the lysates (0.75 μg) were separated by 10–20% SDS–PAGE, transferred to a PVDF membrane, blotted with 10,000-fold-diluted HRP-rabbit anti-GFP antibody or 3,000-fold-diluted HRP-mouse anti-HA monoclonal antibody (Direct-Blot HRP anti-HA.11 Epitope Tag antibody) and visualized by Immobilon Western Chemiluminescent HRP substrate.

### Establishment of stable transformants and the phospholipid scrambling assay

PLB and *BSG*^*−/−*^ *NPTN*^*−/−*^ W3 cells were transformed with a pantropic retrovirus as described^9^. Briefly, HEK293T cells were transfected with a pMXs-Puro or pCX4-bsr vector together with pGP (Takara Bio), and pCMV-VSV-G-RSV-Rev (RIKEN). Virus was concentrated by centrifugation at 6,000*g* for 16 h, and used to infect cells. Stable transformants were selected with 1 μg ml^−1^ puromycin or 10 μg ml^−1^ blasticidin. *TMEM16F*^*−/−*^*Xkr8*^*−/−*^Ba/F3 cells^16^ were transfected by electroporation using NEPA21 (Nepa Gene), and selected with 0.5–1 mg ml^−1^ G418 or 1.0 µg ml^−1^ puromycin. Stable transformants were observed to localize Xkr8-GFP by confocal microscopy (FV-1000D; Olympus).

Apoptosis was induced by incubating cells with 10 units per ml of FasL for 1 h at 37 °C as described^9^. Phospholipid scrambling was assayed by the exposure of PtdSer or PtdEtn, or the internalization of NBD-SM^9,41,63^. The PtdSer exposure was detected by incubating cells with 1,000-fold-diluted Cy5-Annexin V in Annexin V buffer (10 mM HEPES-NaOH buffer (pH 7.4), 140 mM NaCl and 2.5 mM CaCl_2_) containing 5 μg ml^−1^ propidium iodide followed by flow cytometry using FACSCanto II (BD Biosciences)^9,41^. The data were analyzed by FlowJo v.7.6.5 and FACSDiva v.6.1.3 (BD Biosciences). For NBD-SM-incorporation, cells (1.0 × 10^6^ cells per ml) were incubated with 0.5 μM NBD-SM in Annexin V buffer. After incubation, an aliquot was mixed with an equal volume of Annexin V buffer containing 5 mg ml^−1^ fatty-acid-free BSA (Sigma-Aldrich) and 5 nM SYTOX red (Thermo Fisher Scientific), and analyzed by FACSCanto II (ref. ^9^). The gating strategies in flow cytometry for the cells that bound Annexin V or incorporated NBD-SM are presented in Supplementary Fig. 4. The PtdEtn exposure was examined by Ro 09-0198-induced cytotoxicity as described^36^. In brief, cells at 1.0 × 10^5^ per ml in Annexin V buffer containing 5 mg ml^−1^ BSA were incubated with Ro 09-0198-biotin, and the lactate dehydrogenase (LDH) activity in the supernatant was measured with the LDH Cytotoxicity Detection Kit (Takara). The respective scrambling activity of mutant mXkr8-GFP was normalized by its expression level (mean fluorescence intensity of their GFP) compared with that of the wild-type mXkr8-GFP except for parental Ba/F3 cells.

### LC–MS/MS analysis of phospholipids

To extract lipids from hXkr8–hBSGΔ, the protein was mixed and vortexed with 1 ml of methanol/chloroform/water (10:5:3, v/v/v) as described^64,65^. The mixture was sonicated at 4 °C with a Bioruptor II (Cosmo Bio) at maximum power for 30 s five times with 30-s intervals, and spun at 16,000*g* for 5 min. To a 700-µl aliquot of the supernatant, 195 μl of chloroform and 195 μl of water were added, and the aqueous and organic layers were separated by centrifugation. The 200 μl of the organic layer was evaporated in a SpeedVac concentrator and dissolved in 100 µl of 10 mM ammonium formate containing 25% (v/v) isopropanol and 25% (v/v) acetonitrile. LC–MS/MS analysis was performed using an UltiMate 3000 HPLC coupled with a Q Exactive Hybrid Quadrupole Orbitrap mass spectrometer (Thermo Fisher Scientific). The mass spectrometer was equipped with an electrospray ionization source. The LC–MS conditions with Phenomenex Kinetex C8 column (Shimadzu GLC) were: injection volume, 1 or 2 μl; mobile phase A, 20 mM ammonium formate; mobile phase B, isopropanol/acetonitrile (1:1, v/v); flow rate, 0.2 ml min^−1^; modifier (B) gradient: 20% at 0–1 min, 45% at 2 min, 92.5% at 25 min, 100% at 26–35 min, 20% at 35.1–41 min; column temperature, 45 °C. The MS tune conditions were: ionization mode, positive and negative; capillary voltage, 3.5 kV for both positive and negative ionization; capillary temperature, 350 °C. The following full mass scan/data-dependent MS/MS (full MS/dd-MS^2^) mode was used for phospholipid analysis: scan range, 500–1,100 *m/z*; mass resolution, 70,000; AGC target, 3 × 10^6^; maximum injection time, 100 ms. The parameters for data-dependent product ion scanning were: mass resolution, 17,500; AGC target, 1 × 10^5^; maximum injection time, 100 ms; loop count, 5; isolation width, 2.0 *m/z*; stepped normalized collision energy, 15, 25 and 35. The LC–MS/MS system was controlled by Chromeleon 6.8 and Xcalibur 4.0 software (Thermo Fisher Scientific). Phospholipid species were analyzed by Xcalibur Qual Browser 4.3 software.

### Statistics and reproducibility

Statistical analysis was performed using RStudio v.1.1.463. Error bars indicate standard deviation. For all experiments presented as representative images in Figs. 1a,b, 2e, 3b and 5g and Extended Data Fig. 1b, three to five biological replicates were performed. The experiments presented in Figs. 2c and 3c were performed twice. The experiments presented in Fig. 1a (SDS–PAGE) and Extended Data Fig. 1d,e were performed more than five times. All other experiments were performed at least three times as described in the legend for each figure.

### Reporting Summary

Further information on research design is available in the Nature Research Reporting Summary linked to this article.

## Online content

Any methods, additional references, Nature Research reporting summaries, source data, extended data, supplementary information, acknowledgements, peer review information; details of author contributions and competing interests; and statements of data and code availability are available at 10.1038/s41594-021-00665-8.

## Supplementary information

Supplementary Table 1 and Figs. 1–4.

Reporting Summary

Peer Review Information

## Data Availability

The cryo-EM density map for hXkr8–hBSGΔ–Fab18 was deposited in the Electron Microscopy Data Bank (accession number: EMD-30636). The coordinates for the models of the hXkr8–hBSGΔ–Fab18 complex were deposited in the Protein Data Bank (PDB) under the accession code PDB 7DCE. The coordinates and structural factors of Fab14 and the hBSG (domain2)–Fab14 complex were deposited in PDB under accession codes PDB 7D9Z and PDB 7DAA, respectively. The mass spectrometry data were deposited in the ProteomeXchange Consortium under accession code PXD 027776. Source data are provided with this paper. Several structural coordinates in the PDB database were used in this study, which can be located by accession numbers 3B5H, 4ma3 and 1ln2.

## Source data

Source Data Fig. 1 Unprocessed CBB-stained gels.
Source Data Fig. 2 Unprocessed western blots and pictures of fluorescent microscope.
Source Data Fig. 3 Unprocessed western blots, CBB-stained gels and pictures of fluorescent microscope.
Source Data Fig. 4 Statistical source data.
Source Data Fig. 5 Unprocessed western blots and CBB-stained gels, pictures of fluorescent microscope.
Source Data Fig. 5 Statistical source data.
Source Data Fig. 6 Statistical source data.
Source Data Extended Data Fig. 1 Unprocessed CBB-stained gels, and pictures of fluorescent microscope.
Source Data Extended Data Fig. 8 Statistical source data.
