# Supplementary material for: The tertiary structure of the human Xkr8–Basigin complex that scrambles phospholipids at plasma membranes
Source: Nat Struct Mol Biol. 2021 Oct 8;28(10):825–34. doi: 10.1038/s41594-021-00665-8 (PMC8500837; doi:10.1038/s41594-021-00665-8)
Supplement: Supplementary file 1 — Supplementary Table 1 and Figs. 1–4. [file 41594_2021_665_MOESM1_ESM.pdf]

nature portfolio

https://doi.org/10.1038/s41594-021-00665-8

Supplementary information

The tertiary structure of the human Xkr8–Basigin complex that scrambles phospholipids at plasma membranes

In the format provided by the authors and unedited

Supplementary Table 1. Primers for mutagenesis of human BSG, and human and mouse Xkr8

hBSG mutants N152Q and N186Q

	forward primer	reverse primer
	TCCGCTAGCGCGGCCAACTCCTAAAAAACCGCCACCATG	TCTCGAATTCGTCGATCACTTGTC
	complementary mutagenizing primers	
N152Q	CTCATGCAAGGCTCCGAGAGCAGGTTCTTC	GGAGCCTTGCATGAGGGCCTTGTCCTCAGAG
N186Q	CGGTGCCAAGGCACCAGCTCCAAGGGC	GGTGCCTTGGCACCGGTACTGGCCGGG

hBSG mutants A207C and P211C

	forward primer	reverse primer
	GTGTCGTGAGGAATTCGCCACCATGGCGGCTGCGCTGTTC	ATGGGTACATGAATTCGGAAGAGTTCCTCTGGCGGAC
	complementary mutagenizing primers	
A207C	CACCTGTGCGCCCTCTGGCCCTTCCTGG	GAGGGCGCACAGGTGGCTGCGCACGC
P211C	CTCTGGTGCTTCCTGGGCATCGTGGCTGAGG	CAGGAAGCACCAGAGGGCGGCCAGGTG

hBSG mutant E230C

	forward primer	reverse primer
	CTAGACTGCCGGATCCGCCACCATGGCGGCTG	ATGGGTACATGAATTCGGAAGAGTTCC
	complementary mutagenizing primers	
E230A	ATCTACGCGAAGCGCCGGAAGCCC	GCGCTTCGCGTAGATGAAGATGATGGTGACCAGC

mXkr8 mutants

	forward primer	reverse primer
	GTGAGGAATTGGATCCATCATGC	ACAGATTCTCGAATTCGAGGACTCC
	complementary mutagenizing primers	
D12A	GCCTTAGCCGTGGTCGTAGGCCTGGTG	GACCACGGCTAAGGCCACATGGTGGTGC
D26A	CTGCTGGCTCTGGTCGCTGACCTGTGG	GACCAGAGCCAGCAGGAAAGACAAGATACTCAC
D30A	GTCGCTGCCCTGTGGGCCGTTGTCCAG	CCACAGGGCAGCGACCAGATCCAGCAGG
R42A	CCTTGGCGCTTATCTGTGGGCCGCGCTG	AGATAAGCGCCAAGGAGCACGTACTGGAC
W45A	TTATCTGGCGGCCGCGCTGGTACTGG	GCGGCCGCCAGATAACGGCCAAGGAGCACG
R98A	CTGTATGCGTGTTTGCACGGAATGCATCAAGG	CAAACACGCATACAGGTAGCCGAGCTGCAG
D129A	TCCCTGGCCATCAGCATGCTGAAGCTTTTCGAG	GCTGATGGCCAGGGAGAGAAAGTCTGCGTAG
K134A	ATGCTGGCGCTTTTCGAGAGCTTCCTGGAGG	GAAAAGCGCCAGCATGCTGATGTCCAGGGAG
E137A	CTTTTCGCGAGCTTCCTGGAGGCGACG	GAAGCTCGCGAAAAGCTTCAGCATGCTGATG
E141A	TTCCTGGCGGCGACGCCACAGCTC	CGTCGCCGCCAGGAAGCTCTCGAAAAGCTTC
Q155A	TGTATTGGCGAATGGCCAGGCGGAATACTACC	CCATTCGCCAATACAATTGCCAGCACCAGTGTG
D180A	CTGCTGGCTTACCATCGGTCTCTGCGTACC	ATGGTAAGCCAGCAGTGCCCACGAGATG
R183A	TACCATGCGTCTCTGCGTACCTGTCTTCCC	CAGAGACGCATGGTAATCCAGCAGTGCCCAC
W310A	GGCACCGCGCTGCCCAGTGGGATCTCATTG	GGGCAGCGCGGTGCCGTGTGTCACCCAG

hXkr8 mutants, R214G, R280E, R284E, and D295K

	forward primer	reverse primer
	AGTTAATTAAGGATCCGCCACCATGCCCTGGTCTAG	CCTTGCTCACGAATTCTCCTTTCACAGGGCTGGCG
	complementary mutagenizing primers	
R214G	GTGGCCCGGAGTGCTGGCCGTGGCC	AGCACTCCGGGCCACAGCAGCAGC
R280E	AGGGCGAGACAAGAGGCAGAGCCATCATC	CTCTTGTCTCGCCCTCGGCCACGTTG
R284E	AGAGGCGAAGCCATCATCCACTTCGCTTTTC	GATGGCTTCGCCTCTTGTCCGGCCCTC
D295K	CTGAGCAAGAGCATCCTGCTGGTGGCTAC	GATGCTCTTGCTCAGCAGAAAAGCGAAGTGG

hXkr8 mutants, T302C and T305C

	forward primer	reverse primer
	GTGAGGAATTGGATCCGCCACCATGCCCTGGTCTAG	ACAGATTCTCGAATTCTCCTTTCACAGGGCTGGCG
	complementary mutagenizing primers	
T302C	GTGGCTTGCTGGGTCACCCACTCTAGCTGG	GACCCAGCAAGCCACCAGCAGGATGCTGTC
T305C	TGGGTCTGCCACTCTAGCTGGCTGCCTAGC	AGAGTGGCAGACCCAGGTAGCCACCAGCAG

*Mutated residues are underlined. The first 15 nucleotides are complementary to each other between the complementary mutagenizing primers. Each mutant was prepared by PCR. Using hBSG, mXkr8 or hXkr8 cDNA as a template, PCR was carried out with the forward primer and the second mutagenizing primer for the 5' part, and with the first mutagenizing primer and the reverse primer for 3' part. The resultant PCR products for the 5' and 3' parts and linearized vector were fused by In-Fusion HD Cloning Kit (Takara).

```
              ---------10---------20---α1---30---------40---------50----α2--60---------70---------80----
human         MPWSSRGALLRDLVLGVLGTAAFLLDLGTDLWAAVQYALGGRYLWAALVLALLGLASVALQLFSWLWLRADPAGLHGSQP----
mouse         MPLSVHHHVALDVVVGLVSILSFLLDLVADLWAVVQYVLLGRYLWAALVLVLLGQASVLLQLFSWLWLTADPTELHHSQL----
Chicken      MAARGPARFGPWQWLLAAAGAAAAVWDAGADVWVAVGYARAGQPGCAALGLGLWAAGSAARQACSWLWFRSDPSTLRPEL-----
Fugu      MRVMGQATISNYSWIDFVFSVIGVFTFFVDWGADVWVATEFYSRGDFFWFGLLVSLMVLSSVLVQMFSWFWLKYDRELPDVCRQSGGG

          ----------α3a--90-------100-α3b---110-------120---α4a-130-------140-------α4b-------160
human     -------PRRCLALLHLLQLGYLYRCVQELRQGLLVWQQEEPSEFDLAYADFLALDISMLRLFETFLETAPQLTLVLAIMLQSGRAE
mouse     -------SRPFLALLHLLQLGYLYRCLHGMHQGLSMCYQEMPSECDLAYADFLSLDISMLKLFESFLEATPQLTLVLAIVLQNGQAE
Chicken   -------SRRPLAALHLLQLGFLYRCLRALRLGWRLCREEAT-EEERDHMAFLTHDISMLRLFETFLENAPQLAVLLYSVLRTNKAE
Fugu      TTVLFGDRVQLSWLLHVLQLGFLCRHISAIRQGFRVWWRQQ---EGSEYAVYLTHDLSMLRLIETFSESAPQLTLMVHVMLCTNRAR

          -------170----α5-180-------190-------200-------210--α6---220-------230---α7--240-------
Human     YYQWVGICTSFLGISWALLDYHRALRTCLPSKPLLGLGSSVIYFLWNLLLLWPRVLAVALFSALFPSYVALHFLGLWLVLLLWVWLQ
mouse     YYQWFGISSSFLGISWALLDYHRSLRTCLPSKPRLGRSSSAIYFLWNLLLLGPRICAIALFSAVFPYYVALHFFSLWLVLLFWIWLQ
Chicken   PSQGMGICTALLCVTWSLLDYHQSLRSFLQDKYEMGWGSSVIYFLWNLFLLCPRILALALFALLWPYGVAVHFPLVWLAMFIWVSLQ
Fugu      TVQ-----------SWMVVDYHRSLRAFLPDKAKQGWGSSLIYFLWNFLLIAPRVAALALFASVVGGFVAVHFLLLWCVFVMWAWLQ

          250-------260--α8---270-------280-------290--α9---300--------310-------320--α10--330---
Human     GTDFMPDPSSEWLYRVTVATILYFSWFNVAEGRTRGRAIIHFAFLLSDSILLVAT-WVTHSSWLPSGIPLQLWLPVGCGCFFLGLAL
mouse     GTNFMPDSKGEWLYRVTMALILYFSWFNVSGGRTRGRAVIHLIFIFSDSVLLVTTSWVTHGTWLPSGISLLMWVTIGGACFFLGLAL
Chicken   GTDFMESPGPEQLYRAMVAVILYFSWFNVAPGRTLYRSIIYHSFILLDSTLLTLA-WLWGHPPEEQRPYLIPVLCAALPCYLLGLLL
Fugu      GTEFMDSVCGEGLYRATVGIIWYFSWFNVAEGQTRGRSIIYHSFITTDGGILLLT-WWCYRDPVQTEPYGLALLVTLLFSYLLGLLF

          ------340--------350--α11--360-------------------------------------------------------
human     RLVYYHWLHPSCCW---KPDPDQVDGARSLLSP-EGYQLPQNRRMTHLAQKFFPKAKDEAASPVKG--------------------
mouse     RVIYYLWLHPSCSW-----DPDLVDGTLGLLSPHRPPKLIYNRRATLLAENFFAKAKARAVLTEEVQLNGVL---------------
hicken    RVTYYGWLHPNVRAQP-RSGGDEVDAEGRHDGVVEFRSISEPSELRSVSGPDLVNSRLRCLARSHFPLSALAEPRLLNGAAAV----
Fugu      KTVYYCCFHPTMRRPPARESSDLPDAEVTFRHFSIQDGAPSSPLLNRRMAAHAARFYSERRAVKNLGGVDAATSSPP----------
```

Supplementary Fig. 1. Sequence alignment of Xkr8 orthologues. Sequences of Xkr8 from humans (UniProt: Q9H6D3), mice (UniProt: Q8C0T0), chickens (Chick) (UniProt; Q49M60), and fugu (UniProt: H2TYQ9) were analyzed by the MUSCLE Program (EMBL-EBI). Numbers above the first line are the amino acid positions for hXkr8. Conserved residues are indicated in red. Eleven α-helices are shadowed and numbered. Negatively and positively charged residues in the lipid layer are highlighted in green, while the hydrophobic amino acids forming the cleft for PtdCho are highlighted in yellow.

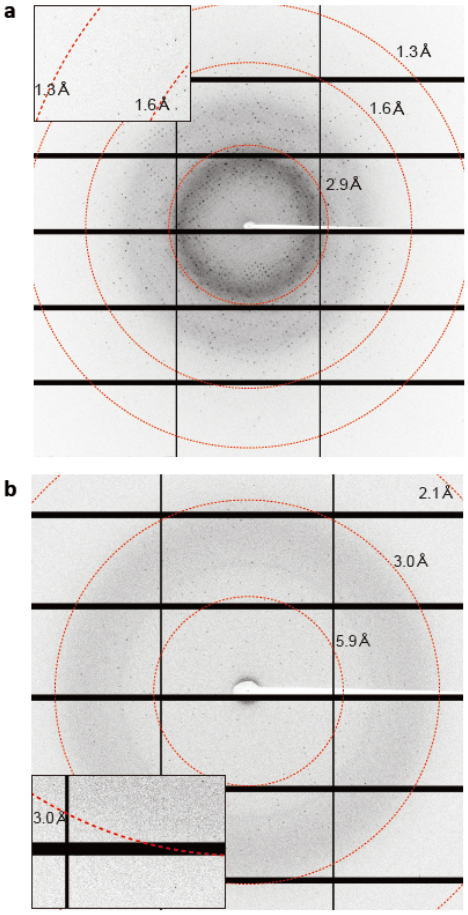

Supplementary Fig. 2. X-ray diffraction analysis of the hBSGΔ-Fab complex. The representative X-ray diffraction pattern of a crystal of Fab14 (**a**) and the lipidated hBSGΔ-Fab complex (**b**) in buffer containing 33% PEG400. The area of high resolution was enlarged in insets.

```
                                            20  α1              40         α2
XKR8 ----------------------MPWSSRGALLRDLVLGVLGTAAFLLDLGTDLWAAVQYALGGRYLWAALVALL
XK   ------------------------------MKFPASVLASVFLFVAETTAALSLSSTYRSGGDRMWQALTLLFS
XKR2 MDRVYEIPEEPNVDPVSSLEEDVIRGANPRFTFPFSILFSTFLYCGEAASALYMVRIYRKNSETYWMTYTFSFF
XKR3 METVFEEMDEESTGGVSSSKEEIVLGQRLHLSFPFSIIFSTVLYCGEVAFGLYMFEIYRKANDTFWMSFTISFI
XKR4 MAAKSDGRLK--85--ALCLRLGREQRRYSLWDCLWILAAVAVYFADVGTDVWLAVDYYLRGQRWWFGLTLFFV
XKR5 -------------------------------MHARLLGLSALLQAAEQSARLYTVAYYFTTGRLLWGWLAL-AV
XKR6 MAAKSDGGGV-101--AARPEPPPPQVERPWLDCLWIVLALLVFFGDVGTDLWLALDYYRKGDYVYFGLTLFFV
XKR7 MAAKSDGAAA--29--PGVVGAGGPGPRYELRDCCWVLCALLVFFSDGATDLWLAASYYLQNQHTYFSLTLLFV
XKR9 ---------------------------MKYTKQNFMMSVLGIIYVTDLIVDIWVSVRFFHEGQYVFSALALSFM

          60                      80     α3a          100 α3b
XKR8 GLASVALQLFSWLWLRADPAGLHG----SQPPRRCLALLHLLQLGYLYRCVQELRQGLLVWQQEEPS-------
XK   LLPCALVQLTL-LFVHRDLSRDRP----------LVLLLHLLQLGPLFRCFEVFCIYFQSGNNEEPY-14-SEE
XKR2 MFSSIMVQLTL-IFVHRDLAKDKP----------LSLFMHLILLGPVIRCLEAMIKYLTLWKKEEQE-16-EVL
XKR3 IVGAILDQIIL-MFFNKDLRRNKA----------ALLFWHILLLGPIVRCLHTIRNYHKWLKNLKQE-11-NTM
XKR4 VLGSLSVQVFSFRWFVHDFSTEDS-72-SFCIWLLQSLIHILQLGQIWRYFHTIYLGIRSRQSGEND-------
XKR5 LLPGFLVQALSYLWFRADGHP----------GHCSLMMLHLLQLGVWKRHWDAALTSLQKELEAPHR-------
XKR6 LVPSLLVQSLSFRWFVQDYTGGGL-41-RLSVWIWQSVIHLLQMGQVWRYIRTMYLGIQSQRRKEHQ-------
XKR7 LLPSLVVQLLSFRWFVYDYSEPAG-53-RLCIWLLQTLVHLLQLGQVWRYLRALYLGLQSRWRGERL-------
XKR9 LFGTLVAQCFSYSWFKADLKKA------GQESQHCFLLLHCLQGGVFTRYWFALKRGYHAAFKYDSN----TSN

             120  α4a            140     α4b          160       α5       180
XKR8 -----EFDLAYADFLALDISMLRLFETFLETAPQLTLVLAIMLQSGR-AEYYQWVGICTSFLGISWALLDYH-R
XK   IEKEVGQAEGKLITHRSAFSRASVIQAFLGSAPQLTLQLYISVMQQD-VTVGRSLLMTISLLSIVYGALRCNIL
XKR2 IEWEVGHSIRTLAMHRNAYKRMSQIQAFLGSVPQLTYQLYVSLISAE-VPLGRVVLMVFSLVSVTYGATLCNML
XKR3 LEREIAFSIRDNFMQQKAFKYMSVIQAFLGSVPQLILQMYISLTIRE-WPLNRALLMTFSLLSVTYGAIRCNIL
XKR4 ----RWRFYWKMVYEYADVSMLHLLATFLESAPQLVLQLCIIVQTHS-LQALQGFTAAASLVSLAWALASYQ-K
XKR5 ---------GWLQLQEADLSALRLLEALLQTGPHLLLQTYVFLASDF-TDIVPGVSTLFSWSSLSWALVSYT-R
XKR6 -----RRFYWAMMYEYADVNMLRLLETFLESAPQLVLQLYIMLQKNS-AETLPCVSSVTSLMSLAWVLASYH-K
XKR7 ----RRHFYWQMLFESADVSMLRLLETFLRSAPQLVLQLSLLVHRGGAPDLLPALSTSASLVSLAWTLASYQ-K
XKR9 FVEEQIDLHKEVIDRVTDLSMLRLFETYLEGCPQLILQLYILLEHGQ-ANFSQYAAIMVSCCAISWSTVDYQ-V

                     200          α6     220             α7  240
XKR8 ALRTCLPSKPLLGL-GSSVIYFLWNLLLLWPRVLAVALFSALFPSYVALHFLGLWLVLLLWVWLQ----GTDFM
XK   AIKIKYDEYEVKVKPLAYVCIFLWRSFEIATRVVVLVLFTSVLKTWVVVIILINFFSFFLYPWILFWCSGSPFP
XKR2 AIQIKYDDYKIRLGPLEVLCITIWRTLEITSRLLILVLFSATLKLKAVPFLVLNFLIILFEPWIKFWRSGAQMP
XKR3 AIQISNDDTTIKLPPIEFFCVVMWRFLEVISRVVTLAFFIASLKLKSLPVLLIIYFVSLLAPWLEFWKSGAHLP
XKR4 ALRDSRDDKKPISY-MAVIIQFCWHFFTIAARVITFALFASVFQLYFGIFIVLHWCIMTFWIVHC----ETEFC
XKR5 FMGFMKPGHLAMPW-AALFCQQLWRMGMLGTRVLSLVLFYKAYHFWVFVVAGAHWLVMTFWLVAQ----QSDII
XKR6 LLRDSRDDKKSMSY-RGAIIQVFWRLFTISSRVISFALFASIFQLYFGIFVVVHWCAMAFWIIHG----GTDFC
XKR7 VLRDSRDDKRPLSY-KGAVAQVLWHLFSIAARGLAFALFASVYKLYFGIFIVAHWCVMTFWVIQG----ETDFC
XKR9 ALRKSLPDKKLLNGLCPKITYLFYKLFTLLSWMLSVVLLLFLNVKIALFLLLFLWLLGIIWAFKN----NTQFC

                     260 α8                        280          α9      300
XKR8 ----------PDPSSEWLYRVTVATILYFSWFNVA----------EGRTRGRAIIHFAFLLSDSI-LLVATWVT
XK   ENIEKALSRVGTTIVLCFLTLLYTGINMFCWSAVQLKIDSPDLISKSHNWYQLLVYYMIRFIENA-ILLLLWYL
XKR2 NNIEKNFSRVGTLVVLISVTILYAGINFSCWSALQLRLADRDLVDKGQNWGHMGLHYSVRLVENV-IMVLVFKF
XKR3 GNKENNSNMVGTVLMLFLITLLYAAINFSCWSAVKLQLSDDKIIDGRQRWGHRILHYSFQFLENV-IMILVFRF
XKR4 ----------ITKWEEIVFDMVVGIIYIFSWFNVK----------EGRTRCRLFIYYFVILLENT-ALSALWYL
XKR5 ----------DSTCHWRLFNLLVGAVYILCYLSFW----------DSPSRNRMVTFYMVMLLENI-ILLLLAT-
XKR6 ----------MSKWEEILFNMVVGIVYIFCWFNVK----------EGRTRYRMFAYYTIVLTENA-ALTFLWYF
XKR7 ----------MSKWEEIIYNMVVGIIYIFCWFNVK----------EGRSRRRMTLYHCIVLLENA-ALTGFWYS
XKR9 ----------TCISMEFLYRIVVGFILIFTFFNIK----------GQNTKCPMSCYYIVRVLGTLGILTVFWVC

                  320      α10        340          α11    360
XKR8 HSSWLPSGIPLQLWLPVGCGCFFLGLALRLVYYHWLHPSCCWKPDPDQVDGARSLLSPEGYQLPQ--25--END
XK   FKTDIYMYVCAPLLVLQLLIGYCTAILFMLVFYQFFHPCKKLFSSSVSEGFQRWLRCFCWACRQQ--42--END
XKR2 FGVKVLLNYCHSLIALQLIIAYLISIGFMLLFFQYLHPLRSLFTHNVVDYLHCVCCHQHPRTRVE--15--END
XKR3 FGGKTLLNCCDSLIAVQLIISYLLATGFMLLFYQYLYPWQSGKVLPGRTENQPEAPYYYVNIEKT--30--END
XKR4 YKAPQIADAFAIPALCVVFSSFLTGVVFMLMYYAFFHPNGPRFGQSPSCACEDPAAAFTLPPDVA-108--END
XKR5 -DFLQGASWTSLQTIAGVLSGFLIGSVSLVIYYSLLHPKSTDIWQGCLRKSCGIAGGDKTERRDS-339--END
XKR6 YRDPETTDSYAVPALCCVFISFVAGIAMMLLYYGVLHPTGPRAKILASSCCAELLWGIPLPPDVE-114--END
XKR7 SRNF-STDFYSLIMVCVVASSFALGIFFMCVYYCLLHPNGPMLGPQAPGCIFRKASEPCGPPADA-111--END
XKR9 PLTIFNPD-YFIPISITIVLTLLLGILFLIVYYGSFHPNRSAETKCDEIDGKPVLRECRMYFLME---------
```

Supplementary Fig. 3. Alignment of amino acid sequences of the human XKR family. Amino acid sequences of XKR8 (Q9H6D3), XK (P51811), XKR2 (Q6PP77), XKR3 (Q5GH77), XKR4 (Q5GH76), XKR5 (Q6UX68), XKR6 (Q5GH73), XKR7 (Q5GH72), and XKR9 (Q5GH70) were aligned by introducing several gaps to obtain maximum homology. Amino acids that were identical among all members are shown in red, while those in the same categories (non-polar: G, A, V, I, L, P, F, W, and M;

uncharged polar: S, T, C, Y, Q, and N; charged polar: D, E, K, R, and H) are in orange. Eleven α-helices are shadowed and numbered. Hydrophobic amino acids forming the cleft carrying PtdCho are highlighted in green, while the charged amino acids that lie in the putative phospholipid path are in yellow. A pair of amino acids in XK (R222 and E327), the mutation of which was identified as missense mutations in McLeod syndrome[34,35], are highlighted in magenta. Numbers above the first line are the amino acid positions for human XKR8.

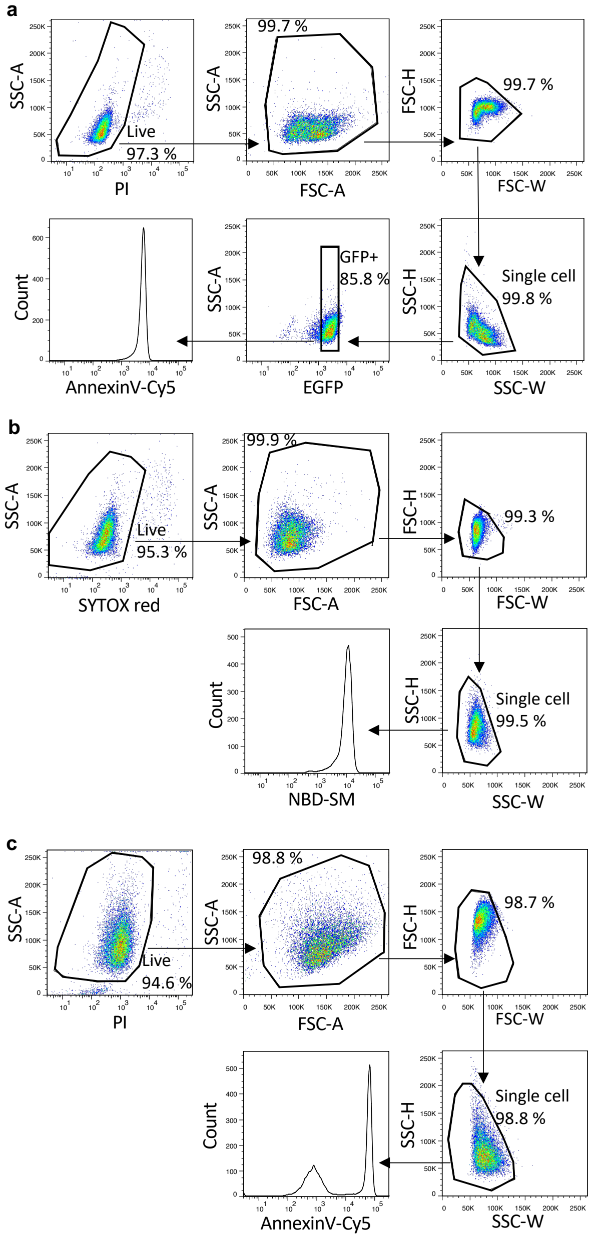

Supplementary Fig. 4. Representative gating strategy for flow cytometric analysis. a, Gating strategy for flow cytometric analysis of Annexin V staining of Ba/F3 transformants expressing the GFP-tagged hXkr8 (Fig. 4**a**,**d**, Fig. 5**h**, and Fig. 6**a**,**b**,**e**,**f**.). **b**, Gating strategy for flow cytometric analysis of NBD-SM incorporation by Ba/F3 transformants (Fig. 4**c**,**d**, Fig. 6**d**, and Extended Data Fig. 8**a**,**b**). **c**, Gating strategy for flow cytometric analysis of Annexin V staining of W3 transformants (Extended Data Fig. 1**c**).
