# Supplementary material for: The tertiary structure of the human Xkr8–Basigin complex that scrambles phospholipids at plasma membranes
Source: Nat Struct Mol Biol. 2021 Oct 8;28(10):825–34. doi: 10.1038/s41594-021-00665-8 (PMC8500837; doi:10.1038/s41594-021-00665-8)
Supplement: Supplementary file 2 — Reporting Summary [file 41594_2021_665_MOESM2_ESM.pdf]

nature research

Corresponding author(s): Shigekazu Nagata

Last updated by author(s): Aug 11, 2021

Reporting Summary

Nature Research wishes to improve the reproducibility of the work that we publish. This form provides structure for consistency and transparency in reporting. For further information on Nature Research policies, see our Editorial Policies and the Editorial Policy Checklist.

Statistics

For all statistical analyses, confirm that the following items are present in the figure legend, table legend, main text, or Methods section.

n/a	Confirmed	
☐	☒	The exact sample size (*n*) for each experimental group/condition, given as a discrete number and unit of measurement
☐	☒	A statement on whether measurements were taken from distinct samples or whether the same sample was measured repeatedly
☒	☐	The statistical test(s) used AND whether they are one- or two-sided<br>*Only common tests should be described solely by name; describe more complex techniques in the Methods section.*
☒	☐	A description of all covariates tested
☒	☐	A description of any assumptions or corrections, such as tests of normality and adjustment for multiple comparisons
☐	☒	A full description of the statistical parameters including central tendency (e.g. means) or other basic estimates (e.g. regression coefficient) AND variation (e.g. standard deviation) or associated estimates of uncertainty (e.g. confidence intervals)
☒	☐	For null hypothesis testing, the test statistic (e.g. *F*, *t*, *r*) with confidence intervals, effect sizes, degrees of freedom and *P* value noted<br>*Give P values as exact values whenever suitable.*
☒	☐	For Bayesian analysis, information on the choice of priors and Markov chain Monte Carlo settings
☒	☐	For hierarchical and complex designs, identification of the appropriate level for tests and full reporting of outcomes
☒	☐	Estimates of effect sizes (e.g. Cohen's *d*, Pearson's *r*), indicating how they were calculated

Our web collection on statistics for biologists contains articles on many of the points above.

Software and code

Policy information about availability of computer code

Data collection	SHIKA as of October 2018, KUMA as of October 2018
Data analysis	autoPROC v1.0.5, STRANISO v2.3.36, CCP4 v7.1, Molrep v11.7.03, Refmac v5.8.0267, PHENIX v1.19rc2_4022, Coot v0.9, CTFFIND v4, RELION v3.1, RStudio v1.1.463, PyMol v2.4.0, UCSF Chimera v1.13.1, UCSF ChimeraX v0.93, FlowJo7.6.5, FACSDiva v6.1.3

For manuscripts utilizing custom algorithms or software that are central to the research but not yet described in published literature, software must be made available to editors and reviewers. We strongly encourage code deposition in a community repository (e.g. GitHub). See the Nature Research guidelines for submitting code & software for further information.

Data

Policy information about availability of data

All manuscripts must include a data availability statement. This statement should provide the following information, where applicable:

- Accession codes, unique identifiers, or web links for publicly available datasets
- A list of figures that have associated raw data
- A description of any restrictions on data availability

The cryo-EM density map for hXkr8-hBSGΔ-Fab18 was deposited in the Electron Microscopy Data Bank (accession number: EMD-30636). The coordinates for the models of the hXkr8-hBSGΔ-Fab18 complex were deposited in the Protein Data Bank (PDB) under the accession code PDB 7DCE. The coordinates and structural factors of Fab14 and the hBSG (domain2)-Fab14 complex were deposited in PDB under accession codes PDB 7D9Z and PDB 7DAA, respectively. The mass spectrometry data was deposited in the ProteomeXchange Consortium under accession code PXD027776. All other data are available from the corresponding authors upon reasonable request. Several structural coordinates in the PDB database were used in this study, which can be located by accession numbers 3B5H, 4ma3, and 1ln2.

Field-specific reporting

Please select the one below that is the best fit for your research. If you are not sure, read the appropriate sections before making your selection.

☒ Life sciences ☐ Behavioural & social sciences ☐ Ecological, evolutionary & environmental sciences

For a reference copy of the document with all sections, see nature.com/documents/nr-reporting-summary-flat.pdf

Life sciences study design

All studies must disclose on these points even when the disclosure is negative.

Sample size	Sample sizes were not pre-determined. X-ray diffraction and Cryo-EM images were collected until structures of satisfactory quality were solved, which suggested sufficient sample size. For biochemical and cellular experiments, no information was derived about a population based on sampling, and therefore sample size determination was not necessary.
Data exclusions	No data were excluded.
Replication	For all experiments presented as representative images in Fig. 1a and 1b, Fig. 2e, 3b, 5g and Extended Data Fig. 1b, 3-5 biological replicates were performed. The experiments presented in Fig. 2c and Fig. 3c were performed twice. The experiments presented in Fig. 1a (SDS-PAGE) and Extended Data Fig. 1d and 1e were performed more than 5 times. All other experiments were performed at least three times and described in legend for each Figure.
Randomization	No group allocation was performed in this study
Blinding	Blinding was not performed as subjective analysis was not needed. Each experiment was analyzed using consistent methods. Quantitative measurements using various approaches and reaction kits as described in the methods minimized biased assessments.

Reporting for specific materials, systems and methods

We require information from authors about some types of materials, experimental systems and methods used in many studies. Here, indicate whether each material, system or method listed is relevant to your study. If you are not sure if a list item applies to your research, read the appropriate section before selecting a response.

Materials & experimental systems

n/a	Involved in the study	
☐	☒	Antibodies
☐	☒	Eukaryotic cell lines
☒	☐	Palaeontology and archaeology
☐	☒	Animals and other organisms
☒	☐	Human research participants
☒	☐	Clinical data
☒	☐	Dual use research of concern

Methods

n/a	Involved in the study	
☒	☐	ChIP-seq
☐	☒	Flow cytometry
☒	☐	MRI-based neuroimaging

Antibodies

Antibodies used	Rabbit HRP-anti-GFP antibody (Anti-GFP pAb-HRP-DirecT) MBL Cat# 598-7 (1: 10000)<br>Mouse HRP-anti-HA mAb (Direct-Blot HRP anti-HA.11 Epitope Tag antibody) (Clone 16B12) BioLegend Cat#901519 (1:3000)<br>Rabbit mAb against human Xkr8-Basigin complex (established in this report) (Clones XBA14 and XBA18)
Validation	HRP-labeled rabbit anti-GFP Ab was validated by MBL for Western blot (https://ruo.mbl.co.jp/bio/e/dtl/A/?pcd=598-7) and previous publications (PMID:23840565, 26272249, 27578797, 27707755, 28369861, 28384198, 28588310, 30367048, and 32866183). HRP-labeled mouse anti-HA mAb was validated by BioLegend for Western blot (https://www.biolegend.com/en-us/products/direct-blot-hrp-anti-ha11-epitope-tag-antibody-14398?GroupID=GROUP26) and previous publications (PMID: 28827538, 28598330, 28082682, 28087701, 28053031, 28069948, 27909245, 27797818, 27834731)<br>Rabbit anti-human Xkr8-BSG mAb (clones XBA14 and XBA18), established in this study, was validated by Flow cytometry with mouse cell transformants expressing or not-expressing human Xkr8-Bsg complex. The Cryo-EM image of Xkr8-Bsg-Fab (XBA18) confirmed the binding of this mAb to the Xkr8-Bsg complex.

Eukaryotic cell lines

Policy information about cell lines

Cell line source(s)	HEK293T (ATCC CRL-3216)

Cell line source(s)	DKO, a derivative of WR19L (ATCC TIB-52)<br>Spodoptera frugiperda (SF) 9 cells (ATCC CRL-1711)<br>Human PLB985 cells, a subline of human HL-60 (ATCC CCL-240)<br>TMEM16-/-Xkr8-/- Ba/F3, a derivative of mouse Ba/F3 (ATCC HB-283)<br>FreeStyle™ 293-F Cells (Thermo Fisher Scientific R79007)<br>EL-4 (ATCC TIB-39)
Authentication	None of the cell lines used were authenticated
Mycoplasma contamination	All cell lines were negative for mycoplasma.
Commonly misidentified lines<br>(See ICLAC register)	Not used

Animals and other organisms

Policy information about studies involving animals; ARRIVE guidelines recommended for reporting animal research

Laboratory animals	New Zealand White (NZW) rabbits, male, age of 13 weeks
Wild animals	This study did not involve wild animals.
Field-collected samples	This study did not involve samples collected from the field.
Ethics oversight	All animal studies were approved by the Animal Care and Use Committee of the Research Institute of Microbial Diseases, Osaka University, and Chugai Pharmaceutical Co.

Note that full information on the approval of the study protocol must also be provided in the manuscript.

Flow Cytometry

Plots

Confirm that:

☒ The axis labels state the marker and fluorochrome used (e.g. CD4-FITC).

☒ The axis scales are clearly visible. Include numbers along axes only for bottom left plot of group (a 'group' is an analysis of identical markers).

☒ All plots are contour plots with outliers or pseudocolor plots.

☒ A numerical value for number of cells or percentage (with statistics) is provided.

Methodology

Sample preparation	DKO cells, Ba/F3 cells, and their derivatives were cultured in RPMI1640 containing 10% FCS, collected by centrifugation, and suspended in Annexin V-staining buffer.
Instrument	BD FACSCanto II (BD Biosciences)
Software	Data was acquired using BD FACSDiva software v6.1.3, and analysed using FlowJo7.6.5
Cell population abundance	In some cases, stable transformant expressing the GFP-tagged XKR8 were sorted for GFP.
Gating strategy	Dead cells were omitted from the analysis by standard SSC/FFC gating, PI or SytoxRed

☒ Tick this box to confirm that a figure exemplifying the gating strategy is provided in the Supplementary Information.
