# Supplementary figures and images for: The tertiary structure of the human Xkr8–Basigin complex that scrambles phospholipids at plasma membranes
Source: Nat Struct Mol Biol. 2021 Oct 8;28(10):825–34. doi: 10.1038/s41594-021-00665-8 (PMC8500837; doi:10.1038/s41594-021-00665-8)

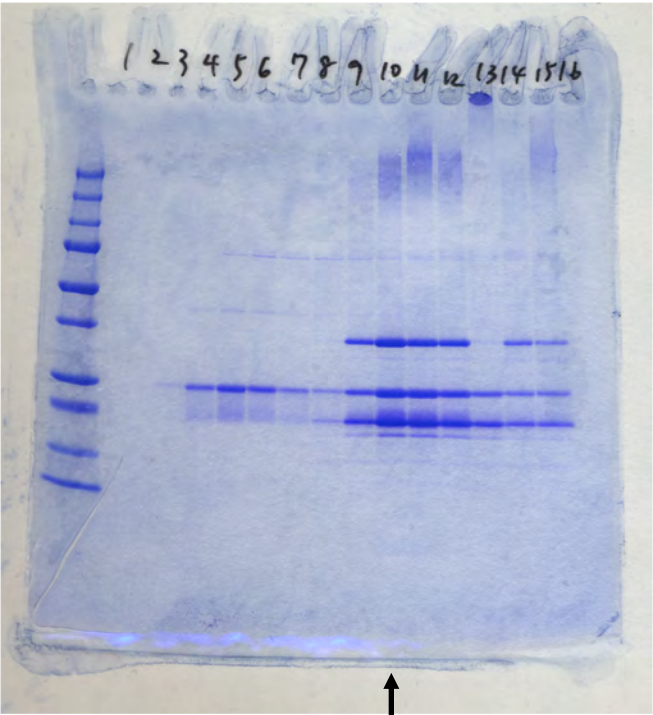

Fig. 1a (Left, Gel)

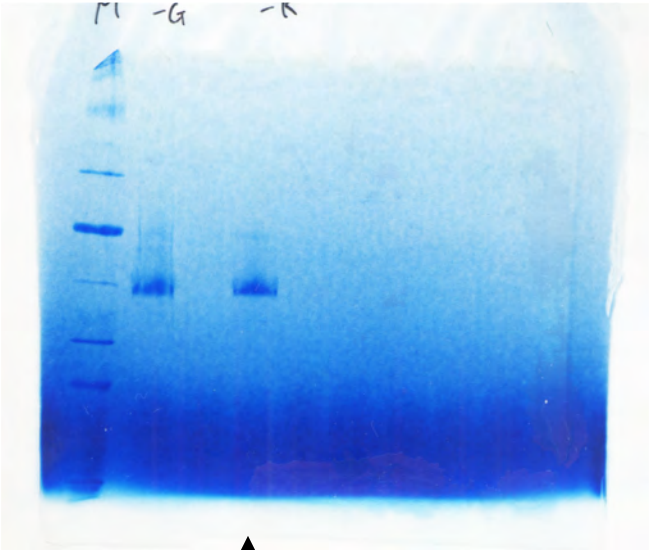

Fig. 1a (Right, Gel)

Supplement: Source Data Fig. 1 — Unprocessed CBB-stained gels. [file 41594_2021_665_MOESM4_ESM.pdf]

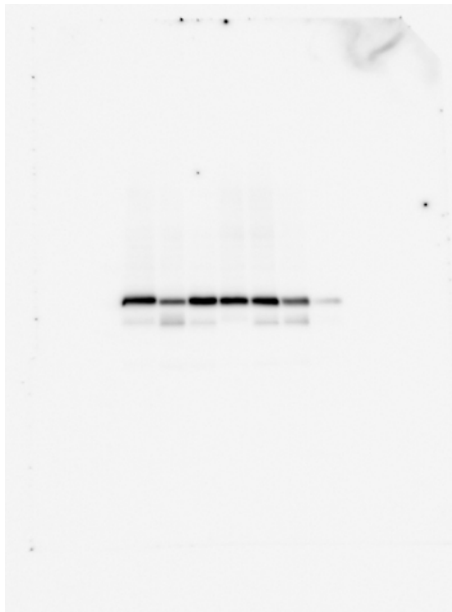

Fig. 5g (Blot)

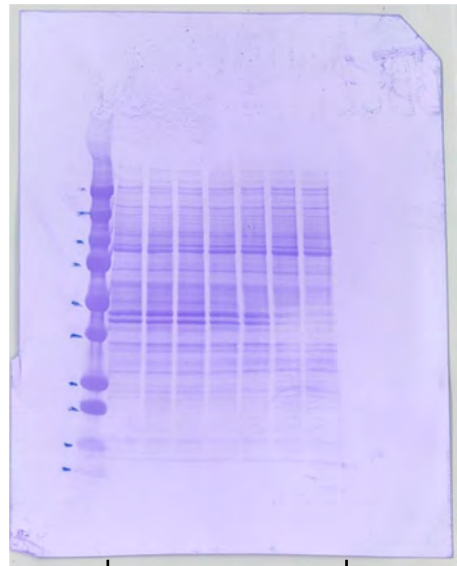

Fig. 5g (CBB staining)

WT

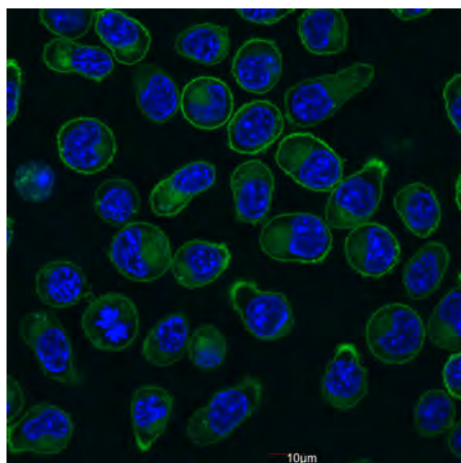

R42A

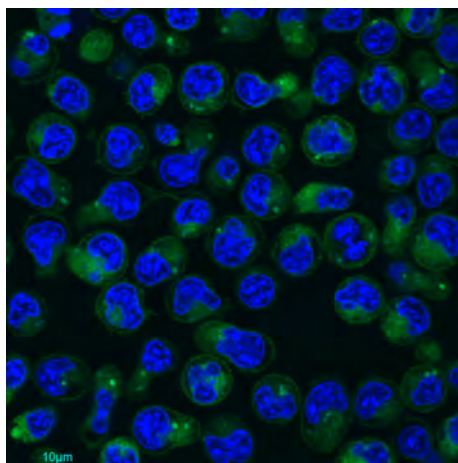

W45A

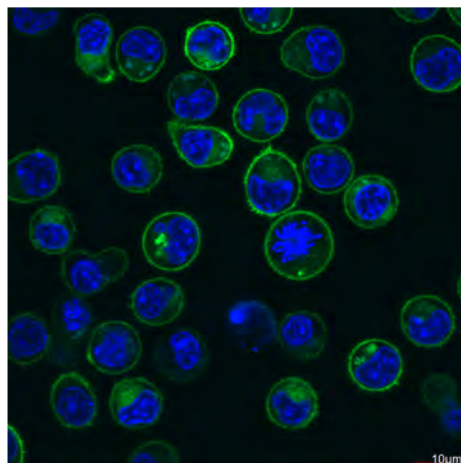

Q155A

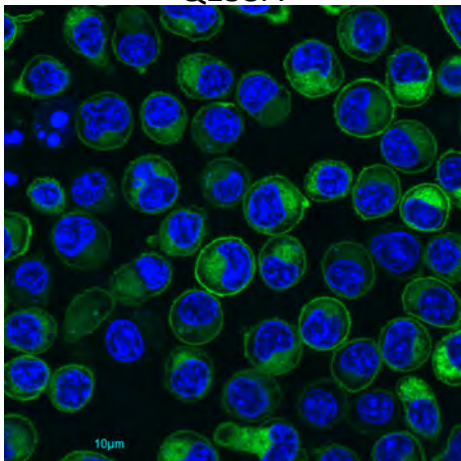

W310A

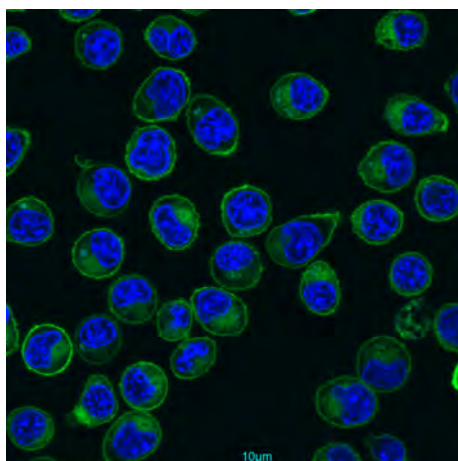

LV-3A

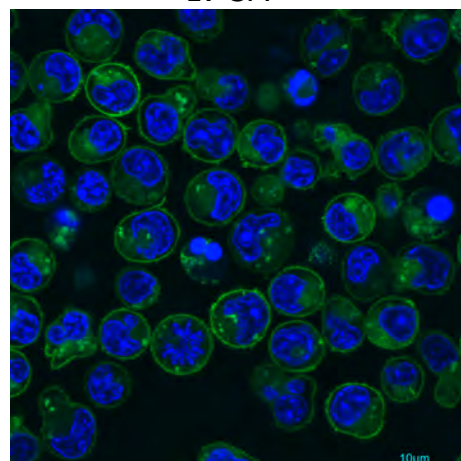

LVF-5A

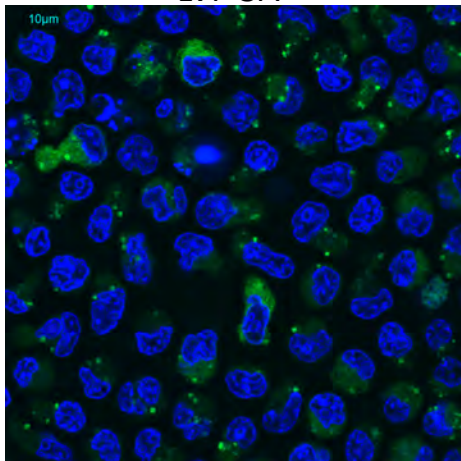

Fig. 5g (Picture)

Supplement: Source Data Fig. 5 — Unprocessed western blots and CBB-stained gels, pictures of fluorescent microscope. [file 41594_2021_665_MOESM8_ESM.pdf]

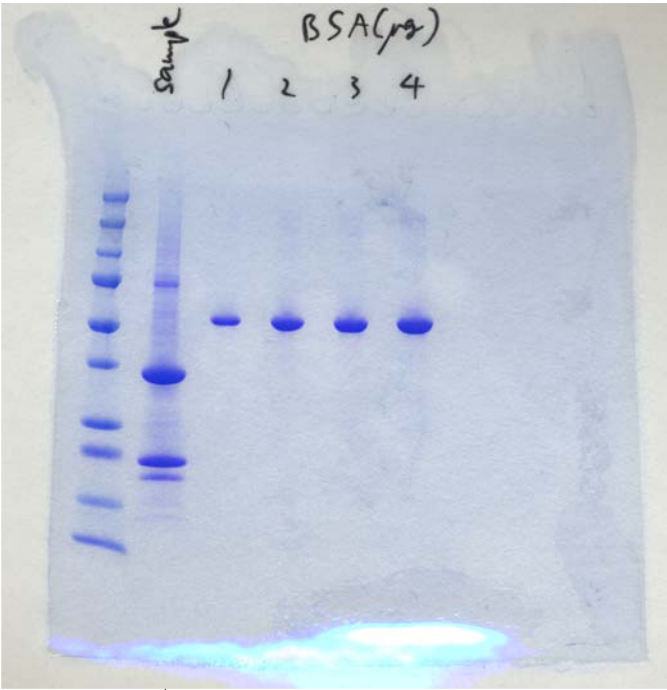

Extended Data Fig. 1d (Left, Gel)

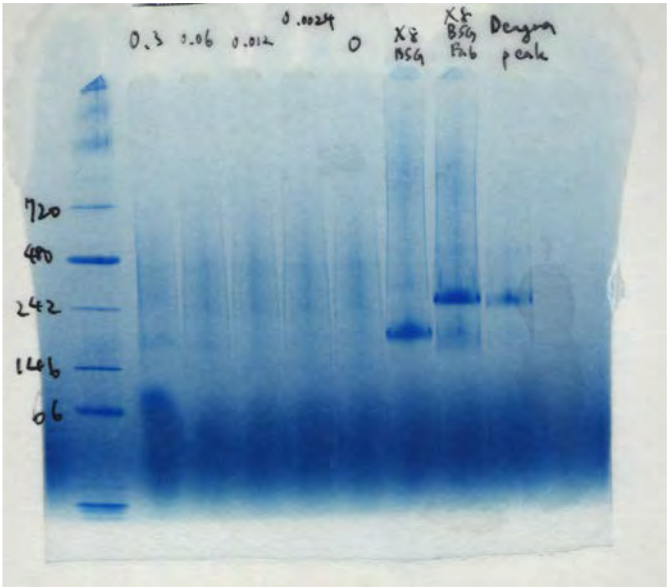

Extended Data Fig. 1d (Right, Gel)

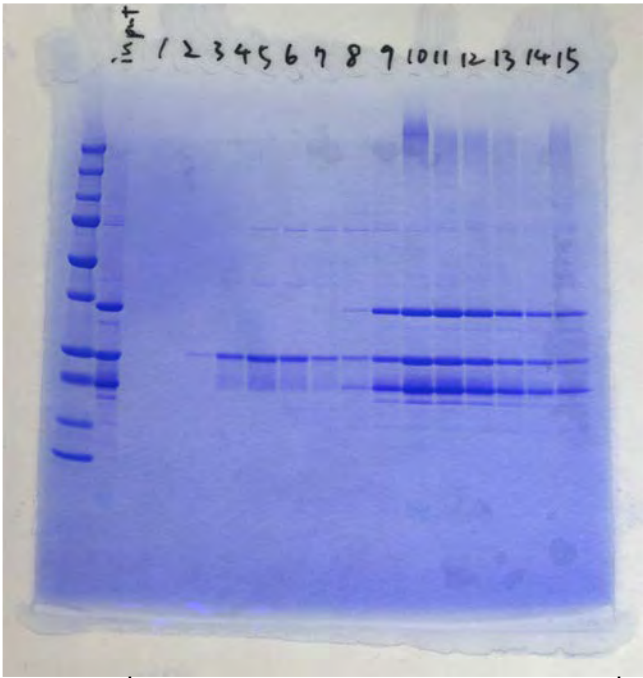

Extended Data Fig. 1e (Gel)

Supplement: Source Data Extended Data Fig. 1 — Unprocessed CBB-stained gels, and pictures of fluorescent microscope. [file 41594_2021_665_MOESM11_ESM.pdf]
