# Supplementary material for: The tertiary structure of the human Xkr8–Basigin complex that scrambles phospholipids at plasma membranes
Source: Nat Struct Mol Biol. 2021 Oct 8;28(10):825–34. doi: 10.1038/s41594-021-00665-8 (PMC8500837; doi:10.1038/s41594-021-00665-8)
Supplement: Source Data Fig. 2 — Unprocessed western blots and pictures of fluorescent microscope. [file 41594_2021_665_MOESM5_ESM.pdf]

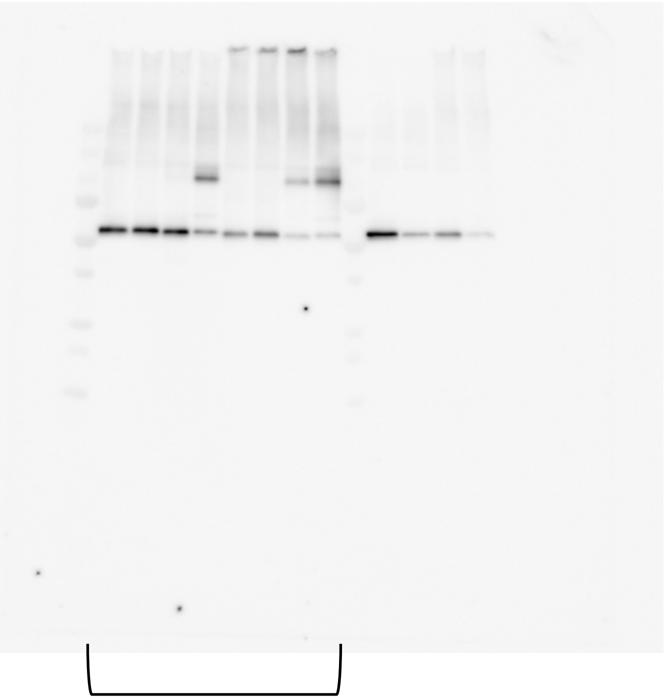

Fig. 2c (Top, Blot)

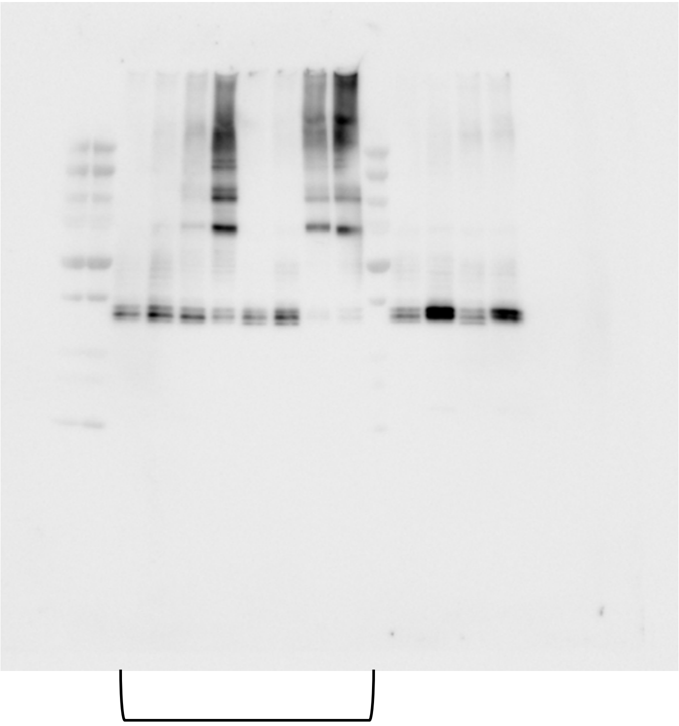

Fig. 2c (Bottom, Blot)

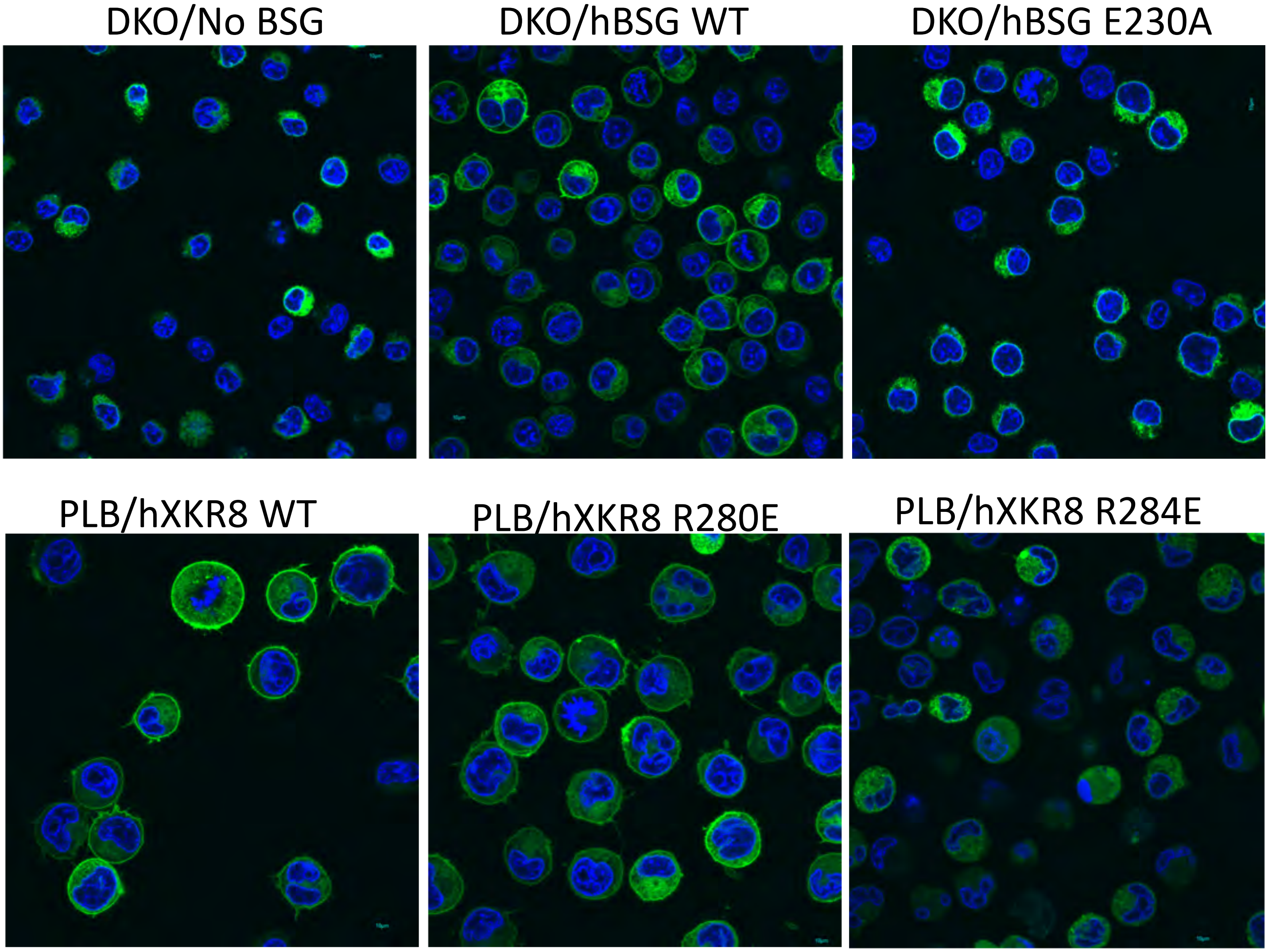

Fig. 2e (Picture)
