# Supplementary material for: The tertiary structure of the human Xkr8–Basigin complex that scrambles phospholipids at plasma membranes
Source: Nat Struct Mol Biol. 2021 Oct 8;28(10):825–34. doi: 10.1038/s41594-021-00665-8 (PMC8500837; doi:10.1038/s41594-021-00665-8)
Supplement: Source Data Fig. 3 — Unprocessed western blots, CBB-stained gels and pictures of fluorescent microscope. [file 41594_2021_665_MOESM6_ESM.pdf]

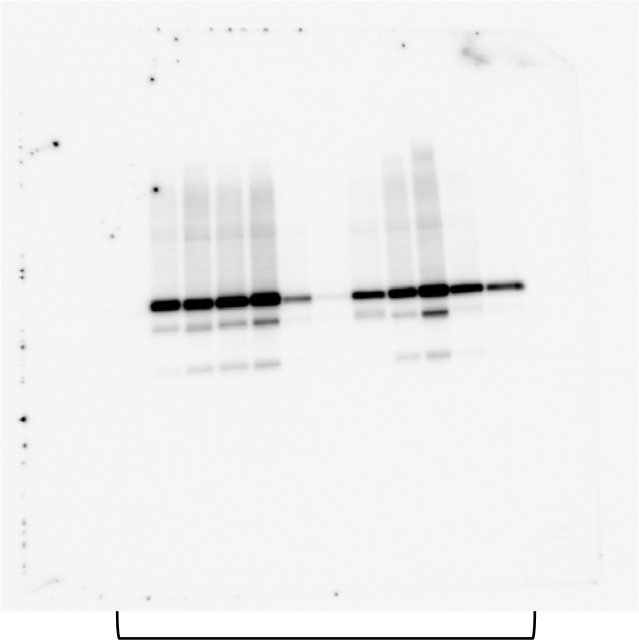

Fig. 3c (Left, Blot)

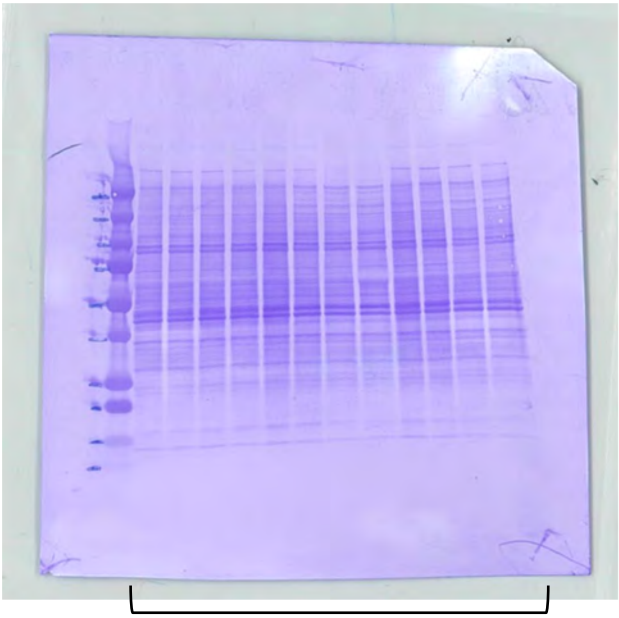

Fig. 3c (Left, CBB staining)

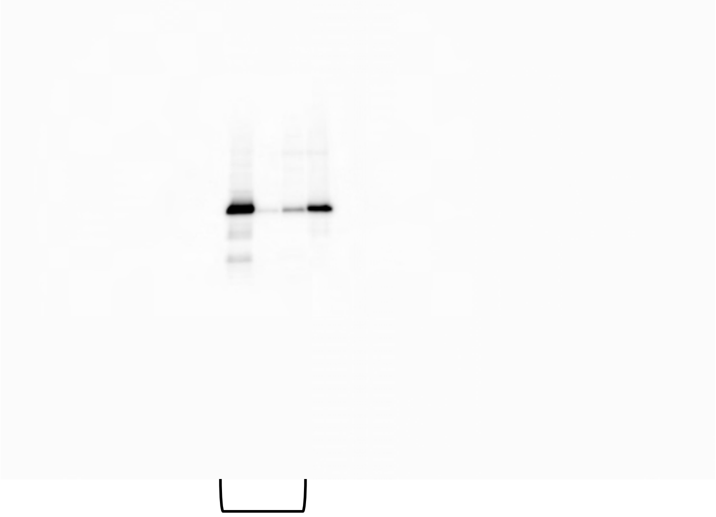

Fig. 3c (Right, Blot)

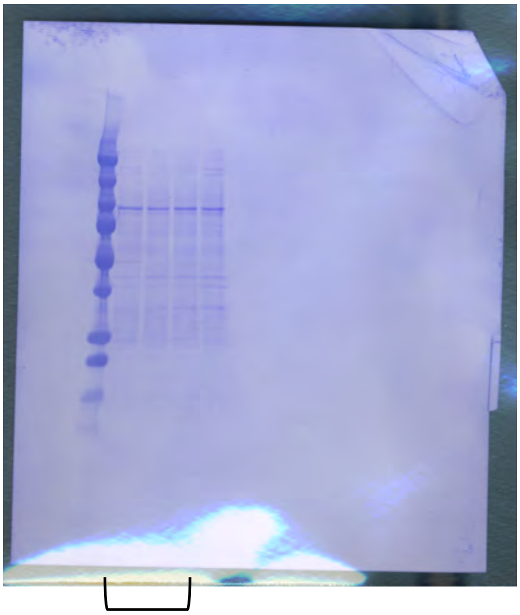

Fig. 3c (Right, CBB staining)

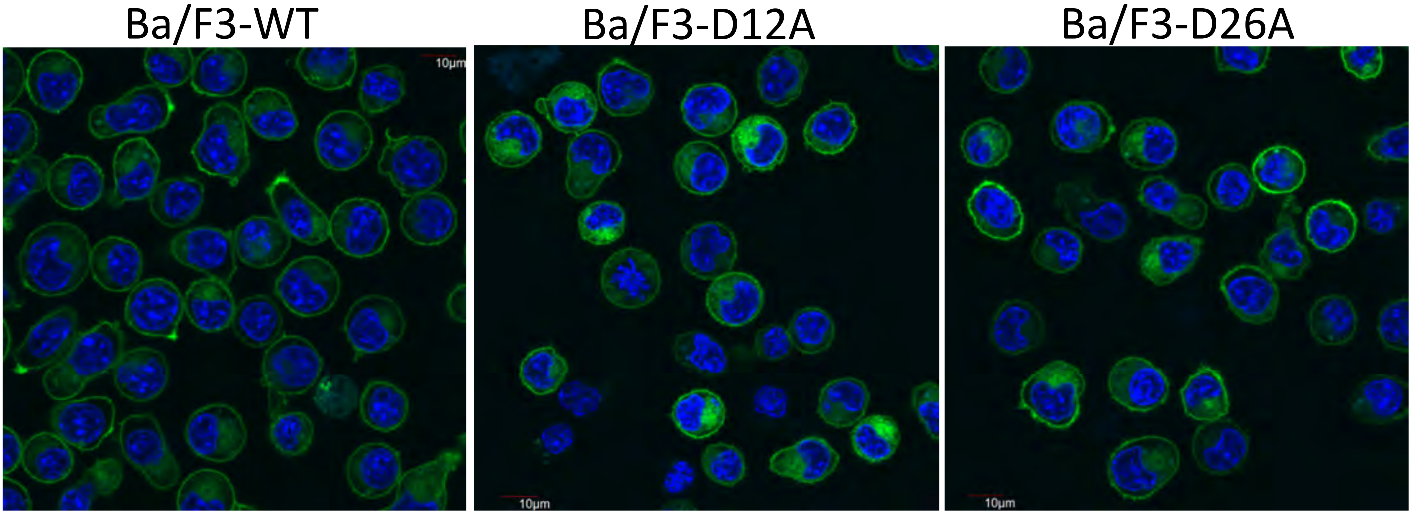

Fig. 3b (Picture)

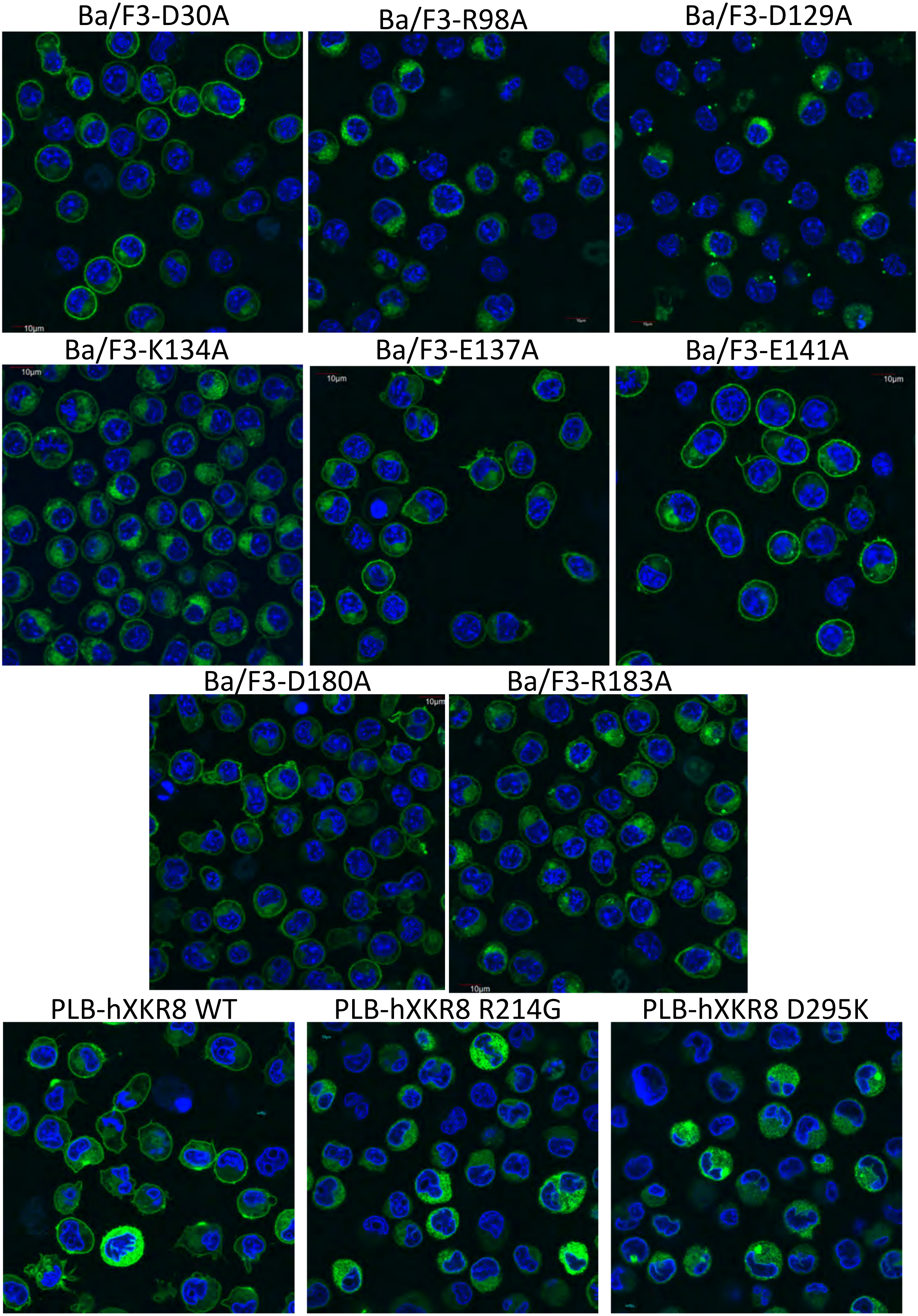

Fig. 3b (Picture)
