# Supplementary material for: The tertiary structure of the human Xkr8–Basigin complex that scrambles phospholipids at plasma membranes
Source: Nat Struct Mol Biol. 2021 Oct 8;28(10):825–34. doi: 10.1038/s41594-021-00665-8 (PMC8500837; doi:10.1038/s41594-021-00665-8)
Supplement: Supplementary file 3 — Peer Review Information [file 41594_2021_665_MOESM3_ESM.pdf]

natureresearch

Peer Review Information

Journal: Nature Structural and Molecular Biology
Manuscript Title: The tertiary structure of the human Xkr8-Basigin complex that scrambles phospholipids at plasma membranes
Corresponding author name(s): Shigekazu Nagata

Reviewer Comments & Decisions:

Decision Letter, initial version:

15th Apr 2021

Dear Shige,

Thank you again for submitting your manuscript "An intramolecular scrambling path controlled by a gatekeeper in Xkr8 phospholipid scramblase". I apologize for the delay in processing your manuscript, which resulted from difficulties in obtaining referees' reports. Nevertheless, we now have comments from 3 reviewers who have evaluated your manuscript (below). You will see that while the referees find the work potentially interesting, all of them raise serious concerns about the strength of the mechanistic claims made. Moreover, reviewers 2 and 3 express major reservations about some of the experimental approaches and the robustness of the data. Based on these comments, I am afraid we cannot offer to publish the study in Nature Structural & Molecular Biology in its current form.

However, if further experimentation, analysis, and revisions allow you to address the referees concerns in full, we would be prepared to consider an appeal of our decision, on the condition that no related work is published in the interim or has been accepted in our journal. If you believe this is a realistic option, please contact me to discuss an appeal and potential revision. Please note that, until we have the opportunity to read the revised manuscript in its entirety, we cannot promise that it will be sent back for peer review.

The required new experiments and data include, but are not limited to:

(1) further functional experiments to confirm and extend the proposed scrambling mechanism, including the roles of residues proposed to act as 'gate keeper' and 'stepping stones';
(2) a protein-specific scrambling assay at room temperature or above, e.g. in proteoliposomes;
(3) further data or analysis to address the problems with the crystallographic data mentioned by reviewer 3.

I would like to stress again that all concerns raised by the referees will have to be addressed to their satisfaction for us to consider publication of a revised version.

I am sorry we could not be more positive on this occasion. I hope that you find the referees' comments useful in deciding how best to proceed.

Kind regards,
Florian

Florian Ullrich, Ph.D.
Associate Editor
Nature Structural & Molecular Biology
ORCID 0000-0002-1153-2040

Referee expertise:

Referee #1: scramblases, structural biology

Referee #2: scramblases, structural biology

Referee #3: phospholipid translocation, structural biology

Reviewers' Comments:

Reviewer #1:

This study examines the structure of the Xkr8 scramblase in complex with the Basigin chaperon. Mutagenesis of residues forming salt bridges between Xkr8 and BSG confirms their involvement in protein interactions required for surface expression of Xkr8. Mutagenesis of 11 charged residues located within the transmembrane domain identified D12A and D180A mutants with constitutive scramblase activity and five alanine substitutions, of D26, D30, E137, E141 and R183, that reduce scramblase activity, leading to their conclusion that these five charged residues provide a path with “stepping stones” for scrambling phospholipids. The authors also tested alanine substitution of W45 leading to their conclusion that this tryptophan at the top of the path “serves as a gatekeeper for the path in mXkr8 to scramble phospholipids or serves as a key residue for transferring phospholipids in both the inward and outward directions at plasma membranes.” However, it is unclear how a tryptophan at or near the extracellular end of the path could fulfill this role.

Why do the authors consider W45 but not D12 and D180 the gatekeeper even though mutations of all three lead to constitutive activation of Xkr8 scramblase? The bound PtdCho has its headgroup coordinated by W45 as well as W309, Q155 and R42 whereas its fatty acid tails contact ten hydrophobic residues. Do mutations of other residues besides W45 that form the binding site of PtdCho affect scramblase activity?

Specific comments:

1. Page 2. “The molecule carried phosphatidylcholine in a cleft on the surface that may function as an entry site for phospholipids.” How would this cleft on the extracellular surface function as an entry site for Xkr8 to expose phosphatidylserine (PtdSer)?
2. Page 2. If the tryptophan at the extracellular end is a gatekeeper, would PtdSer move from the inner leaflet up the hydrophilic pathway to just beneath this tryptophan?
3. Page 3. “The tertiary structure showed that P4-ATPases and TMEM16F family members have hydrophilic clefts between peripheral transmembrane helices…” Please identify the publication that reports the presence of hydrophilic clefts in TMEM16F.
4. Page 8. Please explain the assay used to quantify internalized NBD-SM.
5. Page 9. The bound PtdCho spans the upper leaflet, half the width of the bilayer, as shown in Extended Data Fig. 8. Its two acyl chains contact more than 10 hydrophobic residues. Is this phospholipid supposed to be in the process to be scrambled from the outer leaflet to the inner leaflet? If so, how do the authors envision its dissociation from its long and narrow cleft binding site? How might this cleft function as the entry or exit point of the phospholipid scrambling path?
6. How might mutations of this PtdCho binding site other than W45 affect scramblase activity?
7. Page 9. What exactly does it mean when the authors say “W45 localized at the top of the path appeared to prevent PtdCho from entering the path”? Is W45 located on the extracellular side of PtdCho including its polar headgroup? If so, where do the authors think this PtdCho come from when it enters the path? Shouldn’t it come from the outer leaflet rather than the extracellular side of the membrane?
8. Page 11. “In the extracellular boundary of hXKr8 facing the lipid environment, we detected a hydrophobic cleft occupied by a single PtdCho molecule. The vestibule of this lipophilic cleft was near the extracellular end of the stairway, and a tryptophan (W45) localized between them (Fig. 5c).” This is unclear as to what the vestibule is and what the authors mean by saying that the W45 is between the vestibule and the cleft.
9. It is unclear whether this PtdCho is actually surrounded by hydrophobic residues forming the lipophilic cleft, or it is partially exposed to the membrane interior.
10. Page 12. The proposed model suggesting caspase cleavage or phosphorylation of residues at the C-terminal tail “to open the W45 gate into the hydrophilic cluster. Phospholipids are recruited to the pathway by hydrophilic amino acids, slip down using them as “stepping stones”, and exit through crevasses…” does not seem to naturally connect with “Tryptophan (W176) and tyrosine (Y181) localized to the middle of α5 may destabilize the lipid bilayer and facilitate phospholipid scrambling, as proposed for TMEM16F.” Do the authors find indications for membrane distortion? Are they proposing two very different models with one stemming from their mutagenesis study and the other deriving from the McLeod Syndrome mutations?

Reviewer #2:

The Xkr proteins mediate the externalization of PS and PE in apoptotic cells, which is an essential signal for the recognition and engulfment of dying cells by macrophages. Despite their importance, the structural basis of Xkr lipid scrambling activity remained unknown. The present manuscript reports the structure of the mXkr8/basiginin complex, and therefore provides an invaluable framework to elucidate the basis of phospholipid scrambling by these proteins. The authors show that Xkr8/Basiginin complex adopts a novel fold and identify several regions important for the stability, trafficking and function of the complex. I expect the results presented here to be of interest to a wide audience of readers with diverse interests, from physiology and cell biology to biophysics and structural biology.

Despite its clear merits, the manuscript is weakened by far-fetched mechanistic interpretations of the data. As such, additional experiments are needed to support the proposed mechanism of Xkr-mediated scrambling. Alternatively, the authors should tone down and/or revise some of their statements and proposals.

Major

- The mutagenesis experiments described in Fig. 4 and 5 unambiguously show that the identified charged residues and the Trp side chain are important for scrambling. However, I think that the proposed mechanistic interpretations for their respective roles as stepping-stones and gatekeeper are not well founded for several reasons:
1) The identified residues (D12A, D26A, D30A, E137A, E141A, D180A, and R183A) are mainly negatively charged (6 of 7). How can these negatively charged residues serve as stepping stones for the negatively charged PS headgroups? I would expect that the electrostatic repulsion to prevent PS permeation.
2) Xkr8 scrambles lipids in a non-selective manner. The how can heads with different size/charge/properties be transported non-selectively through an environment that is so highly charged and narrow?
3) The helices harboring the acidic residues are tightly packed against each other. The conformational rearrangements needed to accommodate lipids would be extensive, as these helices would need to move ~10A apart from each other.
4) The authors propose that both lipid heads and tails pass through this highly charged and narrow crevice. However, the charged side chains would present an inhospitable environment for the hydrophobic lipid tails, which would then present a very high barrier to lipid permeation.

Overall, additional evidence supporting the authors' hypothesis is needed. Ideally, the structure of the activated Xkr would clarify this point and eliminate concerns.

- Why are the scrambling experiments performed at 4 oC? Was this done throughout the manuscript, or only for the experiment described in Fig. 4? Scramblase activation should be able to overcome the activity of the flippases at physiological temperatures. Indeed, if the Xkr scramblases have a lower temperature dependence than the flippases, by performing the experiments at 4 oC the authors are selectively dampening the activity of the latter, creating a non-physiological context to evaluate the activity of the mutant proteins. These experiments should be performed at RT or at 37 oC to show that temperature does not play a role.

- The authors identification of W45 as a critical component of Xkr function is remarkable. However, several concerns remain with the authors interpretation of its mechanistic role.
1) Does the W45A mutant induce constitutive activity also in the WT Xkr8, or only on the background of the 3A mutant?
2) Did the authors test the functionality of the W45A mutant at RT or at 37 oC or only at 4 oC?
3) While the finding that the W45A rescues the 3A inhibitory phenotype is interesting, I think that the identification of this residue as a gatekeeper is a bit premature. Other alternatives are possible (i.e. indirect effects on gating or permeation), and should be discussed.

Minor
Pg. 12 The authors state that W176 and Y181 located in the middle of a5 might destabilize the

membrane. Why? Tyr and Trp side chains are generally well tolerated in a membrane environment.
Pg. 7 The argument that replacing the small side chains of hBSG with the larger ones of hEMB results in steric clashes is reasonable. However, this observation alone does not explain the inability of hEMB to chaperone Xkr8, at most it provides a testable hypothesis. Steric clashes of side chains could be resolved with small, local rearrangements of neighboring residues. This should be toned down or experimentally tested for example by showing that the corresponding mutations in hEMB result in enhanced chaperone activity.
Pg. 5 Please clarify if the crystallized protein samples contained the intact Xkr8/hBSGΔ and the TM regions were disordered, or if the isolated soluble domains were crystallized.
Pg. 3, last paragraph and Pg. 11, first paragraph contain imprecisions and missing references.
The authors seem to conflate two proposed mechanisms for TMEM16-mediated scrambling. The credit card mechanism (Pomorski and Menon, 2006) postulates that lipid traverse the membrane with their head group moving through the hydrophilic cleft while exposing their acyl chains to the lipid environment. In contrast, the out of the groove mechanism postulates that the headgroups do not need to enter the hydrophilic cleft during transport, hence the “out of the groove”. Importantly, both mechanisms are compatible with the idea that membrane distortion enables scrambling. These points should be clarified. The authors should refer to Malvezzi et al., PNAS, 2018 when discussing the out of the groove mechanism of scrambling, and to Falzone et al., Elife, 2019 when discussing the membrane distortion proposal, as these ideas were first presented in these publications.

Reviewer #3:

Xkr8 has been previously investigated by the same group, and has been described to be a phospholipid scramblase that is activated by kinase or caspase. Xkr8 was also reported to form a heterodimer with Basigin (BSG), which allows it to localize to the plasma membrane. The activity of scramblases is important, as exposure of phosphatidylserine (PtdSer) to the cell surface have important functional consequences for the cell, e.g., exposure of PtdSer in apoptotic cells that signals macrophages to perform phagocytosis of dying cells. The structure and mechanisms of TMEM16F and other TMEM16 family members scramblases have been fairly well characterized in the past. However, the mechanism by which Xkr8 scramble phospholipids is not known. Elucidating this mechanism would be of interest to the fields of lipid transport and general cell biology.
In this manuscript, the authors elucidate structures of human Xkr8 bound to a truncated construct of BSG and in complex with a Fab. The authors elucidated structures of this complex at 2.51Å by X-ray crystallography and 3.8 Å by single particle cryo-electron microscopy (cryo-EM). The cryo-EM reconstruction map shows a density for a phosphatidylcholine (PtdCho). Based on these structures, the authors perform different biological assays to investigate the potential function of distinct residues and interactions. These results are then summarized in a mechanistic model.

Elucidating structures of Xkr8 would indeed bring some light to its novel mechanism for phospholipids scrambling, which would merit publication in NSMB. However, there are multiple flaws on the structural data presented (X-ray and cryo-EM) weakening any claim on mechanistic aspects. I would also encourage the authors to perform scrambling assays in proteoliposomes as it has been presented in previous studies of TMEM16 scramblases (References 13,14). These assays would add to the qualitative cells-based assays shown here and would help to validate the function of Xkr8-BSG complex in an isolated form. Furthermore, the data showing cells microscopy, SDS-PAGE, and BN-PAGE, lack common practices for figures presentation.

In conclusion, due to the major concerns exposed below and the lack of rigorous presentation of the results, the manuscript in its current form is far from being a solid mechanistic study that merit publication in NSMB.

Major concerns:

1. Page 5. "…..X-ray crystallography of Fab and hXkr8-hBSGD-Fab revealed the structures of their extracellular regions at a high resolution of 2.51 Å….." and "….Based on the density map obtained by cryo-EM and X-ray crystallography data, the structure of the hXkr8-hBSGΔ-Fab complex was elucidated..."

Although the authors obtained crystals of hXkr8-hBSGD-Fab complex that diffracted to 2.51 Å, they performed phasing only with models of the Fab and the domain 2 of BSG. They also mention in the Supplementary material (crystallization section) that they did not see hXkr8 in the electron density map. My conclusion after reading this, is that there are major issues with this data set, potentially there is no hXkr8 in these crystals. There are multiple comments that one can make on this:

- Although only the Fab and domain 2 of BSG were modelled in the electron density map, the Rwork/Rfree factors are very low (0.228/0.283). In my experience, if one is missing a major part of the model in the asymmetric unit (hXkr8 in this case), the Rwork/Rfree factors would not decrease this much as the model is very far from what is the real content of the crystal. What I would interpret in this case, is that this crystal does not contain hXkr8. I would advice the authors to: (i) Unambiguously demonstrate that these crystals have indeed hXkr8, (ii) Show figures of the electron density map of the asymmetric unit, (iii) Show crystal lattice contacts.
- If indeed, hXkr8 is part of the content of these crystals, the authors should do phasing using the model obtained from the cryo-EM data and re-interpret the electron density map.
- The only figures referring to this crystal structure, are two diffractions patterns. Please show figures of (i) Electron density map of the asymmetric unit, individual helices, Fab-contacts, etc. and (ii) Crystal lattice contacts.

2. Cryo-EM map validation.

- Please provide FSC curves for unmasked, masked, and corrected maps.
- The resolution of the transmembrane region of hXrk8 is low, specially at the cytoplasmic side, which contains important functional residues that were mutated based on their interactions with neighbors. Please show density maps for these interaction networks (Fig. 2A, 2D, 3D, 4E, and 5C).

3. Lipid (PtdCho) cryo-EM density and mechanistic relevance.

The authors describe that a PtdCho lipid bound to the cleft formed by TMs 7 and 2 is indicative of an entrance to the scrambling pathway. There are multiple comments that one can make on this:

- Strong claims on functional lipid/ligands should be accompanied by figures showing unambiguous density maps. Only then, the reader would be convinced that the lipid/ligand was indeed observed above the noise level. There is no density for this lipid in the map shown at the end of Ext. Fig. 4D.

Also, it is barely visible in Ext. Fig. 4E. Whereas, in Ext. Fig. 5B is shown isolated. Please include a main Figure that shows the density map of this lipid and all surrounding residues at the same contour level in the last refined map.

• Since phospholipids were not added during the purification. The authors should provide further data that validates that this is indeed a phospholipid.

4. Cell assays

Figure 2E and 3B show important assays where the cellular location of the complex is studied for different mutants. The authors do not provide a figure showing a larger population of cells (currently only 2 or 3 cells are observed). This does not allow the reader to assess whether the phenotype is well distributed and represented. Please provide such figures as supplementary material. If a large proportion of the population do not present the phenotype, the results should be re-interpreted.

5. No protein-specific scrambling assays.

All the assays presented in the manuscript are cells-based assays. This makes difficult the assessment of the real scrambling activity of Xkr8 as there are other lipid transporters present in the membrane (e.g. P4-type ATPases). Although, the authors mention that at 4ºC the activity of such proteins is reduced, so does the activity of Xkr8 since the fluidity of the membrane drastically changes with temperature (lower temperatures = lower fluidity). Thus, I would suggest the authors to perform scrambling assays such as those presented in the TMEM16 publications (References 13,14). Fluorescently labelled phospholipids are commercially available and the assays have been well described in the literature. I consider that making these assays would give strong basis to every claim on the impact of mutations on the scrambling activity of Xkr8.

6. Human Xkr8 (structure) vs mouse Xkr8 (functional data)

• I do understand that mouse Xkr8 and human Xkr8 are very similar (69% identity). However, I would suggest to explicitly mention in the manuscript that the structure and the functional data are obtained from different proteins.

• Please show the superposition of the Homology model of mXkr8 and hXkr8 using cartoon instead of cylinder representation (Ext. Fig. 7). The problem with cylinder representation is that it underestimates the degree of curvature of transmembrane helices. Please also provide, r.m.s.d of Ca superposition.

7. Activation by phosphorylation and caspase is missing

A very important finding made in the past by the same group, is that Xkr8 is a phospholipid scramblase that is activated by kinase or caspase. I was expecting to see a mechanistic explanation on this based on the structural data. However, this is barely mentioned and seems very ambiguous. I would suggest to include a paragraph or a chapter dedicated exclusively to this. This is indeed one of the most important mechanistic aspects of scramblases (activation mechanism).

Minor comments:

8. Page 7 "Using the Coot program, residues in the transmembrane region of hBSG were replaced by the corresponding residues in hEMB (Fig. 2f). The replacement of G214 by valine resulted in a steric crash with L298 of hXkr8, and the replacement of I225 by threonine and Y229 by cysteine abolished their hydrophobic and polar interactions with I287 and R280, respectively. These results explain the inability of hEMB to chaperone Xkr8."

Please indicate what is the percentage of identity and similarity of BSG, NPTN and EMB. Mutating residues in Coot would be valid only if these proteins have an extremely high percentage of identity.

9. Page 6 "….The location of the membrane was tentatively assigned by referring to the position of tryptophan residues near the end of the helices…"

Please show a Figure of the electrostatic surface potential of the complex. Is the surface facing the membrane hydrophobic?

10. Is the functional data presented in Figures 4B, C, and D and 5E normalized against protein expression levels?

11. The hXkr8- hBSGΔ-Fab complex is 110 kDa in size. However, the BN-PAGE (Fig. 1A) shows a size of about 240kDa. Please clarify this.

12. The SDS-PAGE and BN-PAGE figures should show the full gel and molecular weight marker lanes, or provide a supplementary figure where this is shown.

13. Please indicate the type of column used for Size Exclusion Chromatography in Ext. Fig. 2D.

14. The negative staining micrograph in Fig 1A is unnecessary. I would rather add a cryo-EM micrograph.

15. Reference to PDBeFold is missing

16. Please provide a figure with the distances between residues that were crosslinked A207-T305 and T302-P211. They seem far apart in Fig. 2A and 2D. Although, clearly the western blot indicate that crosslinking works fine.

17. Page 7 "…A Western blot analysis showed that the expression of these mutant proteins was markedly weaker than that of wild- type Xkr8 (Fig. 2c). ". You mean Fig 3C

18. Page 7 "…transmembrane region of the complex (Fig. 2d)". You mean Fig. 3D

****

Although we cannot offer to publish your paper in Nature Structural & Molecular Biology, the work may be appropriate for another journal in the Nature Research portfolio. If you wish to explore suitable journals and transfer your manuscript to a journal of your choice, please use our manuscript transfer portal. If you transfer to Nature-branded journals or to the Communications journals, you will not have to re-supply manuscript metadata and files. This link can only be used once and remains active until used.
All Nature Research journals are editorially independent, and the decision to consider your manuscript will be taken by their own editorial staff. For more information, please see our manuscript transfer FAQ page. Note that any decision to opt in to In Review at the original journal is not sent to the receiving journal on transfer. You can opt in to <i>In Review</i> at receiving journals that support this service by choosing to modify your manuscript on transfer. In Review is available for primary research manuscript types only.

** For Springer Nature Limited general information and news for authors, see http://npg.nature.com/authors.

Author Rebuttal to Initial comments

Reviewer #1:

This study examines the structure of the Xkr8 scramblase in complex with the Basigin chaperon. Mutagenesis of residues forming salt bridges between Xkr8 and BSG confirms their involvement in protein interactions required for surface expression of Xkr8. Mutagenesis of 11 charged residues located within the transmembrane domain identified D12A and D180A mutants with constitutive scramblase activity and five alanine substitutions, of D26, D30, E137, E141 and R183, that reduce scramblase activity, leading to their conclusion that these five charged residues provide a path with "stepping stones" for scrambling phospholipids. The authors also tested alanine substitution of W45 leading to their conclusion that this tryptophan at the top of the path "serves as a gatekeeper for the path in mXkr8 to scramble phospholipids or serves as a key residue for transferring phospholipids in both the inward and outward directions at plasma membranes." However, it is unclear how a tryptophan at or near the extracellular end of the path could fulfill this role.

We agree that it is fascinating to see how the extracellular region of Xkr8 regulates phospholipids' inward and outward translocation. We believe that a similar situation applies to TMEM16, which does not show the continuous conductive pathway for phospholipids (Lee et al., 2018). The intracellular phospholipid entry site is wide enough for phospholipids, but the exocellular entry site is too narrow for phospholipid to access the path. Khelashvill et al. (2020) proposed that the phospholipids serve as substrates and provide a mechanically responsive environment for the scramblase. A phospholipid that has entered the pathway widens the path (Khelashvili et al., 2020). We think that a similar mechanism operates for the Xkr8 scramblase. Once W45 turns around, phospholipids enter from the extracellular entry site and widen the path to open the cytoplasmic entry site. Once it is open,

phospholipid can travel in an opposite direction. We discussed this point in the text with a model (Fig. 7).

Why do the authors consider W45 but not D12 and D180 the gatekeeper even though mutations of all three lead to constitutive activation of Xkr8 scramblase?

Sorry for the unclear presentation of the data. Mouse (m)Xkr8 scramblase is activated by a kinase(s) in IL-3 dependent mouse Ba/F3 cells. The Ba/F3 stable transformants expressing mXkr8 expose phosphatidylserine (PtdSer) at 4°C but not at room temperature or higher temperature. In Fig. 4A of the original manuscript, the PtdSer exposure was examined at 4°C with the transformants expressing various Xkr8 mutants and shows that the transformants of D12A and D180A expose PtdSer just like the transformants expressing the wild-type Xkr8. On the other hand, the transformants expressing other mutants (D26A, D30A, E137A, E141A, and R183A) lost the ability to expose PtdSer. In this condition, the W45A mutant showed significantly enhanced PtdSer-exposure compared with that of the wild-type mXkr8. We showed the results in Fig. 6a of the revised manuscript. To more clearly demonstrate the enhancing effect of W45A, we performed the Annexin V binding assay (PtdSer-exposure) at room temperature. As shown in Fig. 6b of the revised manuscript, the W45A mutants intensely exposed PtdSer, while most of the wild-type did not.

The bound PtdCho has its headgroup coordinated by W45 as well as W309, Q155 and R42 whereas its fatty acid tails contact ten hydrophobic residues. Do mutations of other residues besides W45 that form the binding site of PtdCho affect scramblase activity?

According to the advice of this referee, we mutated W310 in mXkr8 (corresponding to W309 of hXkr8, Q155, and R42 to alanine. As shown in Fig. 5h, the Q155A lost the scrambling activity substantially. Although the expression level of R42A was low, this mutant completely lost the scrambling activity even after normalization with the expression level.

We mutated 3 or 5 hydrophobic residues interacting with the acyl group of phospholipids to Ala (LV-3A; L48A, L148A, and V229A: LVF-5A; L48A, L52A, L148A, V229A, and F233A). Although the 5A mutant was not expressed, the 3A mutant was expressed as efficiently as the wild-type. Yet, this mutant did not show the scrambling activity.

From these results, we think that the narrow hydrophobic cleft on the upper surface of the molecule provides an entry site for the phospholipids at the outer leaflet. We discussed the model in Fig. 7.

Specific comments:

1. Page 2. “The molecule carried phosphatidylcholine in a cleft on the surface that may function as an entry site for phospholipids.” How would this cleft on the extracellular surface function as an entry site for Xkr8 to expose phosphatidylserine (PtdSer)?

We think that the cleft carrying PtdCho serves as an entry site for phospholipids in the outer-leaflet for their inward movement. The cytoplasmic entry site for PtdSer may be closed at this stage. As discussed above, we propose that when a phospholipid enters the pathway, the path would be widened as proposed for TMEM16 and opsin (Khelashvili et al., 2019; Lee et al., 2018; Morra et al., 2018). This would open the cytoplasmic entry site

that may be located between $\alpha1$ and $\alpha5$. We provide current models for the Xkr8's mediated phospholipids-scrambling in Fig. 7.

2. Page 2. If the tryptophan at the extracellular end is a gatekeeper, would PtdSer move from the inner leaflet up the hydrophilic pathway to just beneath this tryptophan?

As discussed above, the cytoplasmic entry site will open when a phospholipid enters from the outer-leaflet entry site to widen the path. A phospholipid in the inner leaflet enters the path only when the entire path widens. The phospholipid from the inner leaflet may not be trapped beneath the tryptophan because the outer leaflet gate is already open at this stage.

3. Page 3. "The tertiary structure showed that P4-ATPases and TMEM16F family members have hydrophilic clefts between peripheral transmembrane helices…" Please identify the publication that reports the presence of hydrophilic clefts in TMEM16F.

We removed the sentence.

4. Page 8. Please explain the assay used to quantify internalized NBD-SM.

This was described in the Method section of the original manuscript (page 12). "To incorporate NBD-SM, cells were washed with Annexin V buffer and incubated on ice for the indicated time with 0.5 μM NBD-SM at 1.0×10^6 cells/ml in Annexin V buffer. A 150-μl aliquot was mixed with an equal volume of Annexin V buffer containing 5 mg/ml fatty acid-free BSA (Sigma-Aldrich) and 5 nM SYTOX red (Thermo Fisher Scientific), and analyzed by FACSCanto II for the mean fluorescence intensity (MFI)." In this method, cells are incubated with the fluorescent-labeled NBD-SM, and the NBD-SM that are remained in the outer leaflet of the plasma membrane are extracted with fatty acid-free BSA. Then, the NBD-SM internalized or translocated into the inner leaflet is measured by FACS. This is widely used to assay the scrambling activity for the inward translocation (Haest et al., 1981; Kay et al., 2012; Suzuki and Nagata, 2014).

5. Page 9. The bound PtdCho spans the upper leaflet, half the width of the bilayer, as shown in Extended Data Fig. 8. Its two acyl chains contact more than 10 hydrophobic residues. Is this phospholipid supposed to be in the process to be scrambled from the outer leaflet to the inner leaflet? If so, how do the authors envision its dissociation from its long and narrow cleft binding site? How might this cleft function as the entry or exit point of the phospholipid scrambling path?

This is a very interesting question. The W45A mutant works as a constitutive active scramblase, suggesting that a large conformational change is probably unnecessary for its activation. Since the cleft is open to the lipid layer of the outer leaflet, we think that this cleft serves as the entry site for phospholipids. Phospholipid can be tightly trapped in the cleft as predicted by the referee. But, the association between ligand and template is a dynamic process, or ligands repeat rapid association and dissociation with the target protein in a nano-second scale (Boehr et al., 2018). When the tryptophan is moved away, there is a space between $\alpha2$ and $\alpha4b$ at the outer layer. The phospholipid may be recruited to the hydrophilic path via positively charged amino acids such as Q163 and Q145. Our model is presented in Fig. 7. We hope that the structure of the Xkr8 scramblase presented

in this manuscript will be a template for further studies to understand the molecular mechanism of how Xkr8 functions as a scramblase.

6. How might mutations of this PtdCho binding site other than W45 affect scramblase activity?

According to the advice of this referee, we mutated W310, Q155, and R42 to alanine. Three or 5 hydrophobic residues that interact with the acyl chain of PtdCho were also mutated to Ala (LV-3A; L48A, L148A, and V229A: LVF-5A; L48A, L52A, L148A, V229A, and F233A). The LV-3A mutant was well expressed in the plasma membrane, but lost the scrambling activity. The Q155A and W310A mutants were expressed well at the plasma membrane, but its scrambling activity was substantially lost (Fig. 5g and 5h). From these results, we propose that in addition to stabilizing the complex, PtdCho in the hydrophobic cleft is involved in scrambling phospholipids.

7. Page 9. What exactly does it mean when the authors say "W45 localized at the top of the path appeared to prevent PtdCho from entering the path"? Is W45 located on the extracellular side of PtdCho including its polar headgroup? If so, where do the authors think this PtdCho come from when it enters the path? Shouldn't it come from the outer leaflet rather than the extracellular side of the membrane?

The W45 is located next to the PtdCho's headgroup. The hydrophobic cleft is open to the outer leaflet of the membrane. We, as the referee, therefore think that phospholipids come from the outer leaflet of the plasma membranes. The mutational analysis indicates that the hydrophilic residues (D26, D30, E137, E141, R183, and K134) play an important role in scrambling phospholipids. Therefore, we propose that the PtdCho's head enters the pathway as proposed in the credit card model. In addition, the inhibitory effect of K134A and R183A suggests that phospholipids may get out from the space between $\alpha1$ and $\alpha5$. Although the space is narrow, we think that the inserted phospholipid will widen the pathway as proposed for TMEM16 and opsin (Khelashvili et al., 2020). Once this space is widened, the space between $\alpha1$ and $\alpha5$ may serve as the entry site for phospholipids at the inner leaflet. We describe our model in Fig. 7. We also provide an alternative "credit card" model.

8. Page 11. "In the extracellular boundary of hXKr8 facing the lipid environment, we detected a hydrophobic cleft occupied by a single PtdCho molecule. The vestibule of this lipophilic cleft was near the extracellular end of the stairway, and a tryptophan (W45) localized between them (Fig. 5c)." This is unclear as to what the vestibule is and what the authors mean by saying that the W45 is between the vestibule and the cleft.

Sorry for the unclear word of the vestibule. We removed the word, and explained our model in Fig.7.

9. It is unclear whether this PtdCho is actually surrounded by hydrophobic residues forming the lipophilic cleft, or it is partially exposed to the membrane interior.

The structure shown in Fig. 5a, 5b, and 5c shows that one side of the phospholipid is open to the lipid layer of the outer leaflet. We modified Fig. 5b to more clearly indicate this point.

10. Page 12. The proposed model suggesting caspase cleavage or phosphorylation of residues at the C-terminal tail "to open the W45 gate into the hydrophilic cluster. Phospholipids are recruited to the pathway by hydrophilic amino acids, slip down using them as "stepping stones", and exit through crevasses…" does not seem to naturally connect with "Tryptophan (W176) and tyrosine (Y181) localized to the middle of α5 may destabilize the lipid bilayer and facilitate phospholipid scrambling, as proposed for TMEM16F." Do the authors find indications for membrane distortion? Are they proposing two very different models with one stemming from their mutagenesis study and the other deriving from the McLeod Syndrome mutations?

Sorry for the confusing presentation. In this manuscript, we have not addressed whether the membrane is distorted or not. Accordingly, we deleted the sentence about membrane distortion in the Discussion. In the scrambling mechanism, we concentrated on the pathway identified by the mutational analysis. The McLeod syndrome mutation helped us to understand the structure of Xkr8, but did not suggest the scrambling mechanism. The Xkr8 carries many hydrophilic residues in the transmembrane regions. Our mutational analysis allowed us to divide these residues into two groups. One group (R98, D129, R214, and D295) for Xkr8's stability and/or localization to plasma membranes. And another (D26, D30, K134, E137, E141, R183) for passing phospholipids. The McLeod mutations belong to the former group.

References

Boehr, D.D., D'Amico, R.N., and O'Rourke, K.F. (2018). Engineered control of enzyme structural dynamics and function. Protein Sci. *27*, 825-838.

Haest, C.W., Plasa, G., and Deuticke, B. (1981). Selective removal of lipids from the outer membrane layer of human erythrocytes without hemolysis. Consequences for bilayer stability and cell shape. Biochim. Biophys. Acta *649*, 701-708.

Kay, J.G., Koivusalo, M., Ma, X., Wohland, T., and Grinstein, S. (2012). Phosphatidylserine dynamics in cellular membranes. Mol. Biol. Cell *23*, 2198-2212.

Khelashvili, G., Cheng, X., Falzone, M.E., Doktorova, M., Accardi, A., and Weinstein, H. (2020). Membrane lipids are both the substrates and a mechanistically responsive environment of TMEM16 scramblase proteins. J. Comput. Chem. *41*, 538-551.

Khelashvili, G., Falzone, M.E., Cheng, X., Lee, B.-C., Accardi, A., and Weinstein, H. (2019). Dynamic modulation of the lipid translocation groove generates a conductive ion channel in Ca2+-bound nhTMEM16. Nat. Commun. *10*, 4972.

Lee, B.-C., Khelashvili, G., Falzone, M., Menon, A.K., Weinstein, H., and Accardi, A. (2018). Gating mechanism of the extracellular entry to the lipid pathway in a TMEM16 scramblase. Nat. Commun. *9*, 3251.

Morra, G., Razavi, A.M., Pandey, K., Weinstein, H., Menon, A.K., and Khelashvili, G. (2018). Mechanisms of Lipid Scrambling by the G Protein- Coupled Receptor Opsin. Structure *26*, 356-367.

Suzuki, J., and Nagata, S. (2014). Phospholipid scrambling on the plasma membrane. Method Enzymol. *544*, 381-393.

Reviewer #2:

The Xkr proteins mediate the externalization of PS and PE in apoptotic cells, which is an essential signal for the recognition and engulfment of dying cells by macrophages. Despite their importance, the structural basis of Xkr lipid scrambling activity remained unknown. The present manuscript reports the structure of the mXkr8/basigignin complex, and therefore provides an invaluable framework to elucidate the basis of phospholipid scrambling by these proteins. The authors show that Xkr8/Basigignin complex adopts a novel fold and identify several regions important for the stability, trafficking and function of the complex. I expect the results presented here to be of interest to a wide audience of readers with diverse interests, from physiology and cell biology to biophysics and structural biology.

We thank the referee for the carefully reading our manuscript and his/her kind comments on our work.

Despite its clear merits, the manuscript is weakened by far-fetched mechanistic interpretations of the data. As such, additional experiments are needed to support the proposed mechanism of Xkr-mediated scrambling. Alternatively, the authors should tone down and/or revise some of their statements and proposals.

As recommended by the referee, we toned down our claim and changed the title in the revised manuscript.

Major

- The mutagenesis experiments described in Fig. 4 and 5 unambiguously show that the identified charged residues and the Trp side chain are important for scrambling. However, I think that the proposed mechanistic interpretations for their respective roles as stepping-stones and gatekeeper are not well founded for several reasons:

1) The identified residues (D12A, D26A, D30A, E137A, E141A, D180A, and R183A) are mainly negatively charged (6 of 7). How can these negatively charged residues serve as stepping stones for the negatively charged PS headgroups? I would expect that the electrostatic repulsion to prevent PS permeation.

We agree that among the 5 (not 7: D12A and D180A do not lose the scrambling activity) charged residues in the proposed pathway, four (D26, D30, E137, and E141) are acidic residues. We now found that K134A loses the scrambling activity (Fig. 4 of the revised manuscript). In addition, we can find two other residues (Q145 and W176) carrying positive charge in the pathway. We discussed our model (Fig. 7). We speculate that repulsion between the acidic residues and negatively charged phospholipid head group may widen the path.

2) Xkr8 scrambles lipids in a non-selective manner. The how can heads with different size/charge/properties be transported non-selectively through an environment that is so highly charged and narrow?

3) The helices harboring the acidic residues are tightly packed against each other. The conformational rearrangements needed to accommodate lipids would be extensive, as these helices would need to move ~10A apart from each other.

These are interesting questions. We believe that a similar question was raised for TMEM16 scramblase in which its Ca^{2+} bound structure does not show the continuous conductive pathway for phospholipids (Lee et al., 2018). The intracellular phospholipid entry site is wide enough for phospholipids, but the exocellular entry site is too narrow for phospholipids to access the path. Khelashvill et al. (2020) propose that phospholipids serve not only as substrates but also provide the mechanically responsive environment for the scramblase. That is, a phospholipid that has entered the pathway widens the path (Khelashvili et al., 2020). We propose a similar mechanism for the Xkr8 scramblase.

The W45A mutant constitutively translocates phospholipids. The position of W45 at the extracellular boundary suggests that its alanine mutation may not cause a big conformational change in the Xkr8/Bsg complex, suggesting that its active form may not be so different from that determined in this manuscript. When the tryptophan moves away, there is a space between α2 and α4b at the outer layer. The phospholipid in the cleft may be recruited into the hydrophilic path via positively charged amino acids such as Q163 and Q145. Phospholipids serve to widen the pathway via the interaction with acidic residues in the pathway. We discussed this in Fig. 7.

4) The authors propose that both lipid heads and tails pass through this highly charged and narrow crevice. However, the charged side chains would present an inhospitable environment for the hydrophobic lipid tails, which would then present a very high barrier to lipid permeation.

The cleft carrying a phospholipid suggests that it is the entry site for the outer leaflet phospholipids. The W45A mutant works as a constitutive-active form, suggesting that it serves as a gatekeeper. Mutations of 6 charged residues inactivated the scramblase activity, suggesting phospholipids pass the hydrophilic pathway. We think that the phospholipid interacts with the acidic residues to widen the path, and basic residue recruits them. As the referee suggested, we also think that the hydrophilic pathway is inhospitable for the hydrophobic lipid tails. But, there are many hydrophobic residues (leucine, isoleucine, and valine) between the α-helixes forming the path, which may help to pass the tail region. We presented our model in Fig. 7.

Overall, additional evidence supporting the authors' hypothesis is needed. Ideally, the structure of the activated Xkr would clarify this point and eliminate concerns.

We agree that the structure of the active form is necessary. We several times tried to analyze the caspase 3-treated Xkr8/Basigin complex. But, this treatment destabilized the complex, and they quickly got aggregated. On the other hand, the W45A mutant works as a constitutive-active form. The localization of W45 suggests that this mutation may not cause a large conformational change in the transmembrane region, and the structure of the "active" form may not be so different from that reported here. According to the advice of the referee, we tone down our claim. This is the third structure for phospholipid scramblase (TMEM16 family, opsin, and Xkr8) and the structure is completely different from TMEM16 and GPCR. We hope that the structure of the Xkr8 scramblase presented in this manuscript will stimulate studies to understand the molecular mechanism of how Xkr8 functions as a scramblase.

- Why are the scrambling experiments performed at 4 ^{o}C? Was this done throughout the

manuscript, or only for the experiment described in Fig. 4? Scramblase activation should be able to overcome the activity of the flippases at physiological temperatures. Indeed, if the Xkr scramblases have a lower temperature dependence than the flippases, by performing the experiments at 4 °C the authors are selectively dampening the activity of the latter, creating a non-physiological context to evaluate the activity of the mutant proteins. These experiments should be performed at RT or at 37 °C to show that temperature does not play a role.

The distribution of phospholipids at the plasma membrane is regulated by two different enzymes (flippases and scramblases). The flippases (ATP11A and ATP11C) that translocate PtdSer and PtdEtn from the outer to inner leaflet at plasma membrane require ATP, and cannot be activated at 4°C. While, scramblases (Xkr8 and TMEM16F) do not require ATP for their scrambling reaction and work even at 4°C, although the temperature increases the kinetics of scrambling (Gyobu et al., 2017; Watanabe et al., 2018). We used these properties to show the Xkr8's scrambling activity for the exposure of PtdSer and PtdEtn. That is, we assayed the exposure of PtdSer and PtdEtn at 4°C where the inward translocation of PtdSer or PtdEtn by flippase is inhibited. (During apoptosis, caspase 3 activates Xkr8 and inactivates flippases (Segawa et al., 2016; Segawa et al., 2014). Thus, the assay at 4°C may reflect this situation.

To respond to the referee's concern, we now carried out the assay for the NBD-SM incorporation at 20°C because this substrate (sphingomyelin) cannot activate the plasma membrane flippases (Segawa et al., 2014). The results were essentially the same as those obtained at 4°C. The Ba/F3 transformants expressing the wild-type Xkr8 efficiently incorporated NBD-SM at 20°C, but several mutants entirely lost their activity. We showed the new data obtained at 20°C in Fig. 4c and 4d and moved the original data at 4°C to the Extended Data Fig. 10.

- The authors identification of W45 as a critical component of Xkr function is remarkable. However, several concerns remain with the authors interpretation of its mechanistic role.

1) Does the W45A mutant induce constitutive activity also in the WT Xkr8, or only on the background of the 3A mutant?

No, the W45A mutant works as a constitutively active form in a wild-type (no 3A-mutation background) Ba/F3. In Fig. 6a, we analyzed the exposure of PtdSer of the Ba/F3 transformants expressing the wild-type and W45A mutant mXkr8 after 1 min incubation at 4°C. PtdSer or Annexin V-binding density of W45A transformants was much higher than that of the wild-type mXkr8.

2) Did the authors test the functionality of the W45A mutant at RT or at 37°C or only at 4°C?

Yes, we tested the functionality of W45A mutant at 20°C and 37°C in the wild-type Ba/F3 transformants. In Fig. 6b-d, we carried out the assay for mXkr8-mediated phospholipid scrambling at 20°C or 37°C. The PtdSer-exposure in the transformants expressing the wild-type Xkr8 was strongly reduced compared with that at 4°C (Fig. 6b). This is because the flippase translocates PtdSer at 20°C from the outer leaflet to the inner leaflet. On the other hand, since the W45A's strong scramblase activity beats the flippase, we observed the W45A-supported PtdSer exposure at 20°C (Fig. 6b). The cinnamycin killed W45A-expressing

cells much more efficiently than the cells expressing the wild-type mXkr8 at 37°C (Fig. 6c). We observed about three times higher scrambling activity with the W45A mutant for the internalization of NBD-SM than the wild-type (Fig. 6d) at 20°C.

3) While the finding that the W45A rescues the 3A inhibitory phenotype is interesting, I think that the identification of this residue as a gatekeeper is a bit premature. Other alternatives are possible (i.e. indirect effects on gating or permeation), and should be discussed.

W45 is located at the extracellular end of the hydrophilic path, and its mutation to alanine causes the molecule constitutive-active. We, therefore, think that W45 works at least one of the gatekeepers. However, as the referee advices, it is also possible that W45 is indirectly involved in scrambling. Therefore, we abandon using the word "gatekeeper". In addition, we discussed an alternative (credit card) model in Fig. 7.

Minor

Pg. 12 The authors state that W176 and Y181 located in the middle of α5 might destabilize the membrane. Why? Tyr and Trp side chains are generally well tolerated in a membrane environment.

We agree with the referee. We removed this discussion. Thank you.

Pg. 7 The argument that replacing the small side chains of hBSG with the larger ones of hEMB results in steric clashes is reasonable. However, this observation alone does not explain the inability of hEMB to chaperone Xkr8, at most it provides a testable hypothesis. Steric clashes of side chains could be resolved with small, local rearrangements of neighboring residues. This should be toned down or experimentally tested for example by showing that the corresponding mutations in hEMB result in enhanced chaperone activity.

To address the concerns of this referee, we prepared a mutant hBSG in which G214 was replaced by valine as found in hEMB. Surprisingly, the mutant still chaperoned hXkr8 to the plasma membrane. Therefore, we removed Fig. 2f of the original manuscript. We thank the referee for her/his accurate advice.

Pg. 5 Please clarify if the crystallized protein samples contained the intact Xkr8/hBSGΔ and the TM regions were disordered, or if the isolated soluble domains were crystallized.

In the density map of the Fab/Xkr8/BsgΔ crystals (Extended Data Fig. 7), we could not find the density for Xkr8 and the transmembrane region of Bsg. Meanwhile, we obtained the structure of Xkr8/Bsg by CryoEM. Using the model obtained by CryoEM, we re-evaluated the X-ray diffraction data. But, we still could not obtain a structure for Xkr8. On the other hand, the density map obtained by CryoEM is good enough to model the Xkr8/Bsg complex, we abandoned the approach with X-ray crystallography. We, therefore, do not know whether the crystals we obtained contained the Xkr8 or its structure was disordered. We mentioned it in the Methods section. I hope that this referee understands the situation.

Pg. 3, last paragraph and Pg. 11, first paragraph contain imprecisions and missing references. The authors seem to conflate two proposed mechanisms for TMEM16-mediated scrambling. The credit card mechanism (Pomorski and Menon, 2006) postulates that lipid traverse the

membrane with their head group moving through the hydrophilic cleft while exposing their acyl chains to the lipid environment. In contrast, the out of the groove mechanism postulates that the headgroups do not need to enter the hydrophilic cleft during transport, hence the "out of the groove". Importantly, both mechanisms are compatible with the idea that membrane distortion enables scrambling. These points should be clarified. The authors should refer to Malvezzi et al., PNAS, 2018 when discussing the out of the groove mechanism of scrambling, and to Falzone et al., Elife, 2019 when discussing the membrane distortion proposal, as these ideas were first presented in these publications.

Thank you for pointing out our mis-understanding. We corrected the citations.

References

Gyobu, S., Ishihara, K., Suzuki, J., Segawa, K., and Nagata, S. (2017). Characterization of the scrambling domain of the TMEM16 family. Proc. Nat. Acad. Sci. USA *114*, 6274-6279.

Khelashvili, G., Cheng, X., Falzone, M.E., Doktorova, M., Accardi, A., and Weinstein, H. (2020). Membrane lipids are both the substrates and a mechanistically responsive environment of TMEM16 scramblase proteins. J. Comput. Chem. *41*, 538-551.

Lee, B.-C., Khelashvili, G., Falzone, M., Menon, A.K., Weinstein, H., and Accardi, A. (2018). Gating mechanism of the extracellular entry to the lipid pathway in a TMEM16 scramblase. Nat. Commun. *9*, 3251.

Segawa, K., Kurata, S., and Nagata, S. (2016). Human type IV P-type ATPases that work as plasma membrane phospholipid flippases, and their regulation by caspase and calcium. J. Biol. Chem. *291*, 762-772.

Segawa, K., Kurata, S., Yanagihashi, Y., Brummelkamp, T., Matsuda, F., and Nagata, S. (2014). Caspase-mediated cleavage of phospholipid flippase for apoptotic phosphatidylserine exposure. Science *344*, 1164-1168.

Watanabe, R., Sakuragi, T., Noji, H., and Nagata, S. (2018). Single-molecule analysis of phospholipid scrambling by TMEM16F. Proc. Nat. Acad. Sci. USA *115*, 3066-3071.

Reviewer #3:

Xkr8 has been previously investigated by the same group, and has been described to be a phospholipid scramblase that is activated by kinase or caspase. Xkr8 was also reported to form a heterodimer with Basigin (BSG), which allows it to localize to the plasma membrane. The activity of scramblases is important, as exposure of phosphatidylserine (PtdSer) to the cell surface have important functional consequences for the cell, e.g., exposure of PtdSer in apoptotic cells that signals macrophages to perform phagocytosis of dying cells. The structure and mechanisms of TMEM16F and other TMEM16 family members scramblases have been fairly well characterized in the past. However, the mechanism by which Xkr8 scramble phospholipids is not known. Elucidating this mechanism would be of interest to the fields of lipid transport and general cell biology.

In this manuscript, the authors elucidate structures of human Xkr8 bound to a truncated construct of BSG and in complex with a Fab. The authors elucidated structures of this complex at 2.51Å by X-ray crystallography and 3.8 Å by single particle cryo-electron microscopy (cryo-EM). The cryo-EM reconstruction map shows a density for a phosphatidylcholine (PtdCho). Based on these structures, the authors perform different biological assays to investigate the potential function of distinct residues and interactions. These results are then summarized in a mechanistic model.

Elucidating structures of Xkr8 would indeed bring some light to its novel mechanism for phospholipids scrambling, which would merit publication in NSMB. However, there are multiple flaws on the structural data presented (X-ray and cryo-EM) weakening any claim on mechanistic aspects. I would also encourage the authors to perform scrambling assays in proteo-liposomes as it has been presented in previous studies of TMEM16 scramblases (References 13,14). These assays would add to the qualitative cells-based assays shown here and would help to validate the function of Xkr8-BSG complex in an isolated form. Furthermore, the data showing cells microscopy, SDS-PAGE, and BN-PAGE, lack common practices for figures presentation.

In conclusion, due to the major concerns exposed below and the lack of rigorous presentation of the results, the manuscript in its current form is far from being a solid mechanistic study that merit publication in NSMB.

We thank the referee for his/her careful reading of our manuscript, and the constructive comments. According to his/her advices, we revised the manuscript as much as we could.

Major concerns:

1. Page 5. "…..X-ray crystallography of Fab and hXkr8-hBSGD-Fab revealed the structures of their extracellular regions at a high resolution of 2.51 Å….." and "….Based on the density map obtained by cryo-EM and X-ray crystallography data, the structure of the hXkr8-hBSGΔ-Fab complex was elucidated..."

Although the authors obtained crystals of hXkr8-hBSGD-Fab complex that diffracted to 2.51 Å, they performed phasing only with models of the Fab and the domain 2 of BSG. They also mention in the Supplementary material (crystallization section) that they did not see hXkr8 in

the electron density map. My conclusion after reading this, is that there are major issues with this data set, potentially there is no hXkr8 in these crystals. There are multiple comments that one can make on this:

• Although only the Fab and domain 2 of BSG were modelled in the electron density map, the Rwork/Rfree factors are very low (0.228/0.283). In my experience, if one is missing a major part of the model in the asymmetric unit (hXkr8 in this case), the Rwork/Rfree factors would not decrease this much as the model is very far from what is the real content of the crystal. What I would interpret in this case, is that this crystal does not contain hXkr8. I would advice the authors to: (i) Unambiguously demonstrate that these crystals have indeed hXkr8, (ii) Show figures of the electron density map of the asymmetric unit, (iii) Show crystal lattice contacts.

• The only figures referring to this crystal structure, are two diffractions patterns. Please show figures of (i) Electron density map of the asymmetric unit, individual helices, Fab-contacts, etc. and (ii) Crystal lattice contacts.

We once again carefully examined the X-ray data and agreed with the referee that there is no XKR8 in this crystal, although we cannot rule out the possibility that XKR8 was in the crystal but disordered. Considering that XKR8 dissociates from BSG in the presence of some kinds of detergents (Suzuki et al., 2016), it is likely that XKR8 was removed from BSG during crystallization in a buffer containing Octaethylene Glycol Monododecyl Ether (C12E8). We mentioned this in the revised manuscript. According to the referee's advice, we added the electron density map of asymmetric unit, and crystal lattice contacts as Extended Data Figures 7 and 4, respectively.

• If indeed, hXkr8 is part of the content of these crystals, the authors should do phasing using the model obtained from the cryo-EM data and re-interpret the electron density map.

According to the referee's advice, we performed molecular replacement using the hXKR8 model obtained from cryo-EM single-particle analysis. But we did not get a reasonable result, which agrees with the referee's assumption that Xkr8 is released from the complex.

2. Cryo-EM map validation.

• Please provide FSC curves for unmasked, masked, and corrected maps.

As requested by the referee, FSC curves for unmasked, masked, and corrected maps are shown in Extended Data Fig. 5.

• The resolution of the transmembrane region of hXrk8 is low, specially at the cytoplasmic side, which contains important functional residues that were mutated based on their interactions with neighbors. Please show density maps for these interaction networks (Fig. 2A, 2D, 3D, 4E, and 5C).

We agree that the resolution of the transmembrane region of hXKR8 is not good enough to determine the side-chain orientation precisely. We, therefore, introduced mutations in these residues and could show that these residues play essential roles in the stabilizing

protein complex or scrambling phospholipids. As requested by the referee, we provide density maps for these interaction networks in Fig. 2a, 2d, 3d, 4e, and 5e of the revised manuscript.

3. Lipid (PtdCho) cryo-EM density and mechanistic relevance.

The authors describe that a PtdCho lipid bound to the cleft formed by TMs 7 and 2 is indicative of an entrance to the scrambling pathway. There are multiple comments that one can make on this:

• Strong claims on functional lipid/ligands should be accompanied by figures showing unambiguous density maps. Only then, the reader would be convinced that the lipid/ligand was indeed observed above the noise level. There is no density for this lipid in the map shown at the end of Ext. Fig. 4D. Also, it is barely visible in Ext. Fig. 4E. Whereas, in Ext. Fig. 5B is shown isolated. Please include a main Figure that shows the density map of this lipid and all surrounding residues at the same contour level in the last refined map.

Thank for the comment. As requested by the referee, we provided the density map of the phospholipid and surrounding residues at the same contour level in Fig. 5c in the revised manuscript, which clearly shows the phospholipid density.

• Since phospholipids were not added during the purification. The authors should provide further data that validates that this is indeed a phospholipid.

According to the referee's comment, we analyzed the purified XKR8/BSG protein by LC-MS/MS and found that it carries phosphatidylcholine (DOPC and POPC). The ratio of DOPC vs POPC corresponds to the lipid composition in Sf9 (Marheineke et al., 1998). Sf9 contains a similar amount of PC and PE (Marheineke et al., 1998). We found a high concentration of PC but very little PE in the complex, which may support that the cleft is the entry site from the outer leaflet of the plasma membrane. (PE is exclusively localized to the inner leaflet, while PC to the outer leaflet.)

4. Cell assays
Figure 2E and 3B show important assays where the cellular location of the complex is studied for different mutants. The authors do not provide a figure showing a larger population of cells (currently only 2 or 3 cells are observed). This does not allow the reader to assess whether the phenotype is well distributed and represented. Please provide such figures as supplementary material. If a large proportion of the population do not present the phenotype, the results should be re-interpreted.

According to his/her advice, we showed figures showing a larger population in Data Source files. Except for R98A, D129A, R214G, and D295K, most GFP-tagged molecules are at the plasma membrane. The western blotting indicated that the mutants (D12A, D26A, D30A, K134A, E137A, E141A, D180A, and R183A) found at the plasma membrane were well expressed. In contrast, the mutants of R98A, D129A, R214G, and D295K were not localized at the plasma membrane and expressed at a low level.

5. No protein-specific scrambling assays.

All the assays presented in the manuscript are cells-based assays. This makes difficult the assessment of the real scrambling activity of Xkr8 as there are other lipid transporters present in the membrane (e.g. P4-type ATPases). Although, the authors mention that at 4ºC the activity of such proteins is reduced, so does the activity of Xkr8 since the fluidity of the membrane drastically changes with temperature (lower temperatures = lower fluidity). Thus, I would suggest the authors to perform scrambling assays such as those presented in the TMEM16 publications (References 13,14). Fluorescently labelled phospholipids are commercially available and the assays have been well described in the literature. I consider that making these assays would give strong basis to every claim on the impact of mutations on the scrambling activity of Xkr8.

We thank the referee for raising this point. We agree that to demonstrate the scramblase activity in Xkr8, the reconstitution with proteo-liposomes is the best. We previously could successfully reconstituteTMEM16F homodimer into the lipid bilayer (Watanabe et al., 2018). On the other hand, we could not incorporate the Xkr8/Bsg complex in the lipid bilayer probably because LMNG that we had to use to purify the complex may not be dissociated from the complex due to its extremely slow off-rate (Chung et al., 2012). Meanwhile, we found that the scramblase (TMEM16F) can function at 4°C in the cell-based as well as proteo-liposome-based assay (Gyobu et al., 2017; Watanabe et al., 2018). As pointed by the referee, the scramblase assay at 4°C should not be influenced by P4-ATPase(s). In addition, the kinetics of the scramblase activity at 4°C show a very nice time-dependent response in the cell-based assay (Gyobu et al., 2017).

Biochemical purification of the Xkr8 complex showed that it is complexed with Basigin or Neuroplastin but not with others (Suzuki et al., 2016). Mutational analysis in this manuscript indicated that many mutations in mXkr8 affect the scrambling activity. Although we cannot formally rule out the possibility that Xkr8 may indirectly activate the scramblase of an unidentified molecule, we think it is acceptable at this stage to regard Xkr8 as a scramblase.

6. Human Xkr8 (structure) vs mouse Xkr8 (functional data)

• I do understand that mouse Xkr8 and human Xkr8 are very similar (69% identity). However, I would suggest to explicitly mention in the manuscript that the structure and the functional data are obtained from different proteins.

Thank you for the comment. We have mentioned it in the text (for example, page 8 and page 11) of the revised manuscript.

• Please show the superposition of the Homology model of mXkr8 and hXkr8 using cartoon instead of cylinder representation (Ext. Fig. 7). The problem with cylinder representation is that it underestimates the degree of curvature of transmembrane helices. Please also provide, r.m.s.d of Ca superposition.

According to his/her advice, we showed the superposition of Homology model for human and mouse Xkr8 using cartoon representation in Extended Data Fig. 9. We calculated r.m.s.d. (Root Mean Square Deviation) with a program (ProFit) (Sippl and Weitckus, 1992) (http://www.proceryon.com/), and shown in Extended Data Fig. 9.

7. Activation by phosphorylation and caspase is missing

A very important finding made in the past by the same group, is that Xkr8 is a phospholipid scramblase that is activated by kinase or caspase. I was expecting to see a mechanistic explanation on this based on the structural data. However, this is barely mentioned and seems very ambiguous. I would suggest to include a paragraph or a chapter dedicated exclusively to this. This is indeed one of the most important mechanistic aspects of scramblases (activation mechanism).

We thank the referee for his/her kind words on our previous works. According to his/her advice, we now prepared a schema (Fig. 7) showing our current model for the stepwise translocation of phospholipids by Xkr8, and explained the model in a paragraph of sentences. We hope that this will be a template for the future analysis of this interesting molecule.

Minor comments:

8. Page 7 "Using the Coot program, residues in the transmembrane region of hBSG were replaced by the corresponding residues in hEMB (Fig. 2f). The replacement of G214 by valine resulted in a steric crash with L298 of hXkr8, and the replacement of I225 by threonine and Y229 by cysteine abolished their hydrophobic and polar interactions with I287 and R280, respectively. These results explain the inability of hEMB to chaperone Xkr8."

Please indicate what is the percentage of identity and similarity of BSG, NPTN and EMB. Mutating residues in Coot would be valid only if these proteins have an extremely high percentage of identity.

To address the concerns of the referee 2, we prepared a mutant hBSG in which G214 was replaced by valine found in hEMB. Surprisingly, the mutant still chaperoned hXkr8 to the plasma membrane. Therefore, we removed Fig. 2F of the original manuscript. We thank the referees for appropriate comments.

9. Page 6 "….The location of the membrane was tentatively assigned by referring to the position of tryptophan residues near the end of the helices…"

Please show a Figure of the electrostatic surface potential of the complex. Is the surface facing the membrane hydrophobic?

According to the advice, we determined the hydrophobicity using the UCSF Chimera X. The results are shown in Extended Data Fig. 8 together with the position of tryptophan. The surface facing the membrane is hydrophobic, and most of the tryptophan residues are present at the boundary between the hydrophilic and hydrophobic regions.

10. Is the functional data presented in Figures 4B, C, and D and 5E normalized against protein expression levels?

All mutant proteins were GFP-tagged, and MFI (mean fluorescent intensity) of GFP reflects their expression level. All the data for the scrambling activity was normalized against MFI of the wild-type mXkr8 in each experiment.

11. The hXkr8- hBSGΔ-Fab complex is 110 kDa in size. However, the BN-PAGE (Fig. 1A) shows a size of about 240kDa. Please clarify this.

The apparent mass of membrane proteins in Blue Native PAGE strongly varies in a detergent- and lipid- dependent manner (Crichton et al., 2013). The migration of membrane proteins in Blue Native PAGE is considerably slow, and its apparent Mr seen by Blue Native PAGE cannot be regarded as the real Mr. (Wittig et al., 2010). We showed this Figure to indicate the homogeneity of the Xkr/Bsg/Fab complex.

12. The SDS-PAGE and BN-PAGE figures should show the full gel and molecular weight marker lanes, or provide a supplementary figure where this is shown.

According to the advice of this referee, we showed the full gel and molecular weight marker lanes in the Data Source Files.

13. Please indicate the type of column used for Size Exclusion Chromatography in Ext. Fig. 2D.

We used Superose 6 Increase 10/300 GL Column. This was described in Method section.

14. The negative staining micrograph in Fig 1A is unnecessary. I would rather add a cryo-EM micrograph.

We now added a typical cryo-EM micrograph as Fig. 1b. We want to keep the negative staining to indicate the monodispersed nature of our sample. We hope that the referee can accept.

15. Reference to PDBeFold is missing

Sorry, we now add the reference (Krissinel and Henrick, 2004) for PDBeFold.

16. Please provide a figure with the distances between residues that were crosslinked A207-T305 and T302-P211. They seem far apart in Fig. 2A and 2D. Although, clearly the western blot indicate that crosslinking works fine.

Thank you for the kind word on our cross-linking experiments. According to her/his advice, we added the distances between A207-T305 and T302-P211 in Fig. 2a

17. Page 7 "…A Western blot analysis showed that the expression of these mutant proteins was markedly weaker than that of wild- type Xkr8 (Fig. 2c). ". You mean Fig 3C

Yes, Fig. 3c. Corrected. Thank you!

18. Page 7 "…transmembrane region of the complex (Fig. 2d)". You mean Fig. 3D

Yes, Fig. 3d. Corrected. Thank you.

References

Chung, K.Y., Kim, T.H., Manglik, A., Alvares, R., Kobilka, B.K., and Prosser, R.S. (2012). Role of detergents in conformational exchange of a G protein-coupled receptor. J. Biol. Chem. *287*, 36305-36311.

Crichton, P.G., Harding, M., Ruprecht, J.J., Lee, Y., and Kunji, E.R.S. (2013). Lipid, detergent, and Coomassie Blue G-250 affect the migration of small membrane proteins in blue native gels: mitochondrial carriers migrate as monomers not dimers. J. Biol. Chem. *288*, 22163-22173.

Gyobu, S., Ishihara, K., Suzuki, J., Segawa, K., and Nagata, S. (2017). Characterization of the scrambling domain of the TMEM16 family. Proc. Nat. Acad. Sci. USA *114*, 6274-6279.

Krissinel, E., and Henrick, K. (2004). Secondary-structure matching (SSM), a new tool for fast protein structure alignment in three dimensions. Acta Cryst. *D60*, 2256-2268.

Marheineke, K., Grünewald, S., Christie, W., and Reiländer, H. (1998). Lipid composition of Spodoptera frugiperda (Sf9) and Trichoplusia ni (Tn) insect cells used for baculovirus infection. FEBS Lett. *441*, 49-52.

Sippl, M.J., and Weitckus, S. (1992). Detection of native-like models for amino acid sequences of unknown three-dimensional structure in a data base of known protein conformations. Proteins *13*, 258-271.

Suzuki, J., Imanishi, E., and Nagata, S. (2016). Xkr8 phospholipid scrambling complex in apoptotic phosphatidylserine exposure. Proc. Nat. Acad. Sci. USA *113*, 9509-9514.

Watanabe, R., Sakuragi, T., Noji, H., and Nagata, S. (2018). Single-molecule analysis of phospholipid scrambling by TMEM16F. Proc. Nat. Acad. Sci. USA *115*, 3066-3071.

Wittig, I., Beckhaus, T., Wumaier, Z., Karas, M., and Schägger, H. (2010). Mass estimation of native proteins by blue native electrophoresis: principles and practical hints. Mol. Cell. Proteomics *9*, 2149-2161.

Decision Letter, first revision:

21st Jul 2021

Dear Shige,

Thank you again for submitting your revised manuscript "The tertiary structure of the human Xkr8-Basigin complex that scrambles phospholipids at plasma membranes". I apologize for the delay in responding, which resulted from the difficulty in securing all the referee reports. Nevertheless, we now have comments (below) from the 3 reviewers who had already evaluated the first version of your

paper. In light of those reports, we remain interested in your study and would like to see your response to the comments of the referees, in the form of a further revised manuscript.

You will see that all reviewers find that the revised manuscript has been significantly improved. However, reviewers #2 and #3 still have remaining concerns that should be addressed. Please be sure to respond to all suggestions of the referees in full in a point-by-point response and highlight all changes in the revised manuscript text file. If you have comments that are intended for editors only, please include those in a separate cover letter. We particularly encourage you to follow reviewer #2's advice to feature the proposed mechanistic models less prominently in the paper due to their speculative nature. Furthermore, we would like to ask you to include a reference to the recent preprint from the Dutzler lab (Straub et al; https://doi.org/10.1101/2021.05.03.442400).

We expect to see your revised manuscript within 2 weeks. If you cannot send it within this time, please contact us to discuss an extension; we would still consider your revision, provided that no similar work has been accepted for publication at NSMB or published elsewhere.

We are committed to providing a fair and constructive peer-review process. Do not hesitate to contact us if there are specific requests from the reviewers that you believe are technically impossible or unlikely to yield a meaningful outcome.

As you already know, we put great emphasis on ensuring that the methods and statistics reported in our papers are correct and accurate. As such, if there are any changes that should be reported, please submit an updated version of the Reporting Summary along with your revision.

Please follow the links below to download these files:

Reporting Summary:
https://www.nature.com/documents/nr-reporting-summary.pdf

Please note that the form is a dynamic 'smart pdf' and must therefore be downloaded and completed in Adobe Reader.

When submitting the revised version of your manuscript, please pay close attention to our href="https://www.nature.com/nature-research/editorial-policies/image-integrity">Digital Image Integrity Guidelines.

Finally, please ensure that you retain unprocessed data and metadata files after publication, ideally archiving data in perpetuity, as these may be requested during the peer review and production process or after publication if any issues arise.

If there are additional or modified structures presented in the final revision, please submit the corresponding PDB validation reports.

SOURCE DATA: we urge authors to provide, in tabular form, the data underlying the graphical representations used in figures. This is to further increase transparency in data reporting, as detailed in this editorial (http://www.nature.com/nsmb/journal/v22/n10/full/nsmb.3110.html). Spreadsheets can be submitted in excel format. Only one (1) file per figure is permitted; thus, for multi-paneled figures, the source data for each panel should be clearly labeled in the Excel file; alternately the data

can be provided as multiple, clearly labeled sheets in an Excel file. When submitting files, the title field should indicate which figure the source data pertains to. We encourage our authors to provide source data at the revision stage, so that they are part of the peer-review process.

Data availability: this journal strongly supports public availability of data. All data used in accepted papers should be available via a public data repository, or alternatively, as Supplementary Information. If data can only be shared on request, please explain why in your Data Availability Statement, and also in the correspondence with your editor. Please note that for some data types, deposition in a public repository is mandatory - more information on our data deposition policies and available repositories can be found below:
https://www.nature.com/nature-research/editorial-policies/reporting-standards#availability-of-data

We require deposition of coordinates (and, in the case of crystal structures, structure factors) into the Protein Data Bank with the designation of immediate release upon publication (HPUB). Electron microscopy-derived density maps and coordinate data must be deposited in EMDB and released upon publication. To avoid delays in publication, dataset accession numbers must be supplied with the final accepted manuscript and appropriate release dates must be indicated at the galley proof stage.

While we encourage the use of color in preparing figures, please note that this will incur a charge to partially defray the cost of printing. Information about color charges can be found at http://www.nature.com/nsmb/authors/submit/index.html#costs

Nature Structural & Molecular Biology is committed to improving transparency in authorship. As part of our efforts in this direction, we are now requesting that all authors identified as 'corresponding author' on published papers create and link their Open Researcher and Contributor Identifier (ORCID) with their account on the Manuscript Tracking System (MTS), prior to acceptance. This applies to primary research papers only. ORCID helps the scientific community achieve unambiguous attribution of all scholarly contributions. You can create and link your ORCID from the home page of the MTS by clicking on 'Modify my Springer Nature account'. For more information please visit please visit www.springernature.com/orcid.

Please use the link below to submit your revised manuscript and related files:

[REDACTED]

Note: This URL links to your confidential home page and associated information about manuscripts you may have submitted, or that you are reviewing for us. If you wish to forward this email to co-authors, please delete the link to your homepage.

We look forward to seeing the revised manuscript and thank you for the opportunity to review your work.

Kind regards,
Florian

Florian Ullrich, Ph.D.
Associate Editor
Nature Structural & Molecular Biology

ORCID 0000-0002-1153-2040

Reviewers' Comments:

Reviewer #1:
Remarks to the Author:
The authors have addressed my comments in the revised manuscript.

Reviewer #2:
Remarks to the Author:
The revised manuscript is improved, and the additional mutagenesis data is interesting. I still have some residual concerns about far-fetched mechanistic interpretation of the data:

1-The authors show that mutating some hydrophobic residues in the cleft that harbors the bound PC molecule impairs scrambling. These residues directly interact with the lipid bound to the scramblase. Wouldn't a simple explanation be that this cleft is part of the transport pathway?

2-Overall, neither of proposed models in Fig. 7 have support of experimental data. While the extensive mutagenesis performed by the authors did show some interesting hits, a coherent picture did not emerge. The series of detailed mechanistic steps presented in Fig. 7 to explain lipid translocation are pure speculation. I think the manuscript would be strengthened by the removal of the models. At least, they should be moved to supplementary information (Figures and discussion) with the addition of disclaimers clarifying that the proposals are the product of speculation. In principle, I appreciate the effort to elaborate and put forth models that can serve as guides for future experiments. However, I am concerned that such a level of detailed description of steps, for which there is no evidence, could mislead readers.
Alternatively, the structure of the W45A constitutively active mutant would provide insights into the process.
Did the authors attempt 3D variability analysis for their structure (Punjani and Fleet; https://doi.org/10.1016/j.jsb.2021.107702)? This could provide hints as to whether some helices are more dynamic than others, as would be demanded by the authors' mechanistic model in Fig. 7.

3-The authors argue that Q145 and W176 carry positive charges in the putative pathway. To the best of my knowledge, Gln and Trp are neutral residues.

4-The authors should acknowledge the work from the Dutzler lab (Straub et al; https://doi.org/10.1101/2021.05.03.442400) that presents the structure of Xkr9, showing that the fold and organization is very similar. This work greatly strengthens the authors' key finding of the structure of the Xkr8/Basiginin complex.

Reviewer #3:
Remarks to the Author:
Comments on "The tertiary structure of the human Xkr8-Basigin complex that scrambles phospholipids

at plasma membranes” by Sakuragi et al.

As this is a review for a revision that I previously did, I will not summarise key results or make other comments except to address the changes made in the revision. Most comments have been answered satisfactorily. The manuscript has improved substantially. I just have a couple of optional comments that the authors might consider before publication.

1. The new extended figure 7 indicates that the electron density for the light and heavy chains of the Fab are of good quality, but of less quality for the BSG-IgII part. My suggestion to the authors (optional) is to remove the crystallographic data since it does not add much to the story. Indeed, the most important structural data comes from the cryo-EM maps.

2. It is nice to see that the mass spectrometry results support the presence of the lipid in the purified samples. However, the new figures showing the cryo-EM reconstruction map for the lipid are difficult to visualize. I would suggest improving the way this is depicted. The same goes for the map of individual residues.

3. During the course of revision, a manuscript by Straub, et al. on the structure of XKR9 was deposited in bioRxiv. I was wondering if it would be worth writing a comparison of the mechanisms proposed by both authors. Both of which depict similarities in how these two proteins work.

Author Rebuttal, first revision:

Rebuttal:

Reviewers' Comments:

Reviewer #1:
The authors have addressed my comments in the revised manuscript.

Thank you!

Reviewer #2:
The revised manuscript is improved, and the additional mutagenesis data is interesting. I still have some residual concerns about far-fetched mechanistic interpretation of the data:

1-The authors show that mutating some hydrophobic residues in the cleft that harbors the bound PC molecule impairs scrambling. These residues directly interact with the lipid bound to the scramblase. Wouldn’t a simple explanation be that this cleft is part of the transport pathway?

We think that the cleft is the entry site or waiting room for phospholipids to scramble. In this sense, the cleft is a part of the transport pathway. The phospholipid in the cleft is blocked by $\alpha8$ from the bottom. Unless a substantial conformation change occurs by the activation, we think it is unlikely that a phospholipid in the cleft travels to the inner leaflet via a path on the same site of the molecule. The W45A mutant works as a constitutive-active form suggests that the active form may have a similar structure as the non-active form. The structure of rat Xkr9 showed that the caspase cleavage does not cause a big conformational change (Straub et al. DOI: 10.7554/eLife.69800).

2-Overall, neither of proposed models in Fig. 7 have support of experimental data. While the extensive mutagenesis performed by the authors did show some interesting hits, a coherent picture did not emerge. The series of detailed mechanistic steps presented in Fig. 7 to explain lipid translocation are pure speculation. I think the manuscript would be strengthened by the removal of the models. At least, they should be moved to supplementary information (Figures and discussion) with the addition of disclaimers clarifying that the proposals are the product of speculation. In principle, I appreciate the effort to elaborate and put forth models that can serve as guides for future experiments. However, I am concerned that such a level of detailed description of steps, for which there is no evidence, could mislead readers.

Alternatively, the structure of the W45A constitutively active mutant would provide insights into the process. Did the authors attempt 3D variability analysis for their structure (Punjani and Fleet; https://doi.org/10.1016/j.jsb.2021.107702)? This could provide hints as to whether some helices are more dynamic than others, as would be demanded by the authors' mechanistic model in Fig. 7.

According to the advice of the referee, we moved Figure 7 showing our models to the Supplemental Materials (Extended Data Fig. 14). Referee 3 asked for a detailed description of our model. We described the experimental results that led us the model for each step. We have shortened the description by removing the last sentence and stressed that this is a working model. I hope that it is acceptable.

3-The authors argue that Q145 and W176 carry positive charges in the putative pathway. To the best of my knowledge, Gln and Trp are neutral residues.

I am sorry. I mentioned in my rebuttal that Gln and Trp carry a positive charge. I agree with the referee that Gln and Trp are neutral residues. It should not be said that they have positive charges. In the text, we described that "the phospholipid in the aperture may approach through $\alpha2$ and $\alpha4b$ to the scrambling path via an interaction with R158, Q163, and Q145."

4-The authors should acknowledge the work from the Dutzler lab (Straub et al;) that presents the structure of Xkr9, showing that the fold and organization is very similar. This work greatly strengthens the authors' key finding of the structure of the Xkr8/Basiginin complex.

We cited Straub et al. (PMID: 34263724). Their result shows that the caspase mediated cleavage does not cause a substantial conformational change in rat Xkr9, supporting our models.

Reviewer #3:
As this is a review for a revision that I previously did, I will not summarise key results or make other comments except to address the changes made in the revision. Most comments have been answered satisfactorily. The manuscript has improved substantially. I just have a couple of optional comments that the authors might consider before publication.

We thank the referee for the kind comments.

1. The new extended figure 7 indicates that the electron density for the light and heavy chains of the Fab are of good quality, but of less quality for the BSG-IgII part. My suggestion to the authors (optional) is to remove the crystallographic data since it does not add much to the story. Indeed, the most important structural data comes from the cryo-EM maps.

We agree with the referee that the crystal structure had little information in the transmembrane region of the complex. However, its structure was very useful or essential to trace the transmembrane region of Xkr8/Basgin complex. We, therefore, want to keep the crystallographic data as it is.

2. It is nice to see that the mass spectrometry results support the presence of the lipid in the purified samples. However, the new figures showing the cryo-EM reconstruction map for the lipid are difficult to visualize. I would suggest improving the way this is depicted. The same goes for the map of individual residues.

Thank you for the kind word on our analysis with mass spectrometry. According to the advice of this referee, we revised Figure 5c to make the lipid and individual residues more visible. I hope that the revised Figure 5c is acceptable.

3. During the course of revision, a manuscript by Straub, et al. on the structure of XKR9 was deposited in bioRxiv. I was wondering if it would be worth writing a comparison of the mechanisms proposed by both authors. Both of which depict similarities in how these two proteins work.

According to the advice, we cited the paper by Straub et al. (PMID: 34263724). Unfortunately, Straub et al. did not do the functional analysis with rat Xkr9. They discuss that Xkr9 may undergo dimerization or form a heterodimer with an unidentified molecule, but do not propose the pathway for phospholipids. It was difficult for us to compare our model with theirs at this stage.

Decision Letter, second revision:

Our ref: NSMB-A44743B

26th Jul 2021

Dear Shige,

Thank you for submitting your revised manuscript "The tertiary structure of the human Xkr8-Basigin complex that scrambles phospholipids at plasma membranes" (NSMB-A44743B). It has now been seen by reviewer #2 (comment below) who finds that the paper has improved in revision, and therefore we'll be happy in principle to publish it in Nature Structural & Molecular Biology, pending minor revisions to comply with our editorial and formatting guidelines.

We are now performing detailed checks on your paper and will send you a checklist detailing our editorial and formatting requirements in about a week. Please do not upload the final materials and make any revisions until you receive this additional information from us.

To facilitate our work at this stage, we would appreciate if you could send us the main text as a word file. Please make sure to copy the NSMB account (cc'ed above).

Thank you again for your interest in Nature Structural & Molecular Biology Please do not hesitate to contact me if you have any questions.

Kind regards,
Florian

Florian Ullrich, Ph.D.
Associate Editor
Nature Structural & Molecular Biology
ORCID 0000-0002-1153-2040

Reviewer #2 (Remarks to the Author):

The authors have addressed my concerns. I have no further requests and recommend the manuscript for publication. This work paves the way to understand how these important proteins function!

Final Decision Letter:

19th Aug 2021

Dear Shige,

We are now happy to accept your revised paper "The tertiary structure of the human Xkr8-Basigin complex that scrambles phospholipids at plasma membranes" for publication as a Article in Nature Structural & Molecular Biology.

Acceptance is conditional on the manuscript's not being published elsewhere and on there being no announcement of this work to the newspapers, magazines, radio or television until the publication date in Nature Structural & Molecular Biology.

Before the manuscript is sent to the printers, we shall make any detailed changes in the text that may be necessary either to make it conform with house style or to make it intelligible to a wider readership. If the changes are extensive, we will ask for your approval before the manuscript is laid out for production. Once your manuscript is typeset you will receive a link to your electronic proof via email within 20 working days, with a request to make any corrections within 48 hours. Please read proofs with great care to make sure that the sense has not been altered. If you have queries at any point during the production process then please contact the production team at rjsproduction@springernature.com. Once your paper has been scheduled for online publication, the Nature press office will be in touch to confirm the details.

Please note that due to tight production schedules, proofs should be returned as quickly as possible to avoid delaying publication. If you anticipate any limitations to your availability over the next 2-4 weeks (such as vacation or traveling to conferences, etc.), please e-mail rjsproduction@springernature.com as soon as possible. Please provide specific dates that you will be unavailable and provide detailed contact information for an alternate corresponding author if necessary.

To assist our authors in disseminating their research to the broader community, our SharedIt initiative provides all co-authors with the ability to generate a unique shareable link that will allow anyone (with or without a subscription) to read the published article. Recipients of the link with a subscription will also be able to download and print the PDF.

As soon as your article is published, you can generate your shareable link by entering the DOI of your article here: <a href="http://authors.springernature.com/share">http://authors.springernature.com/share<a>. Corresponding authors will also receive an automated email with the shareable link

Note the policy of the journal on data deposition: http://www.nature.com/authors/policies/availability.html.

Your paper will be published online soon after we receive proof corrections and will appear in print in the next available issue. You can find out your date of online publication by contacting the production team shortly after sending your proof corrections. Content is published online weekly on Mondays and Thursdays, and the embargo is set at 16:00 London time (GMT)/11:00 am US Eastern time (EST) on the day of publication. Now is the time to inform your Public Relations or Press Office about your paper, as they might be interested in promoting its publication. This will allow them time to prepare an accurate and satisfactory press release. Include your manuscript tracking number (NSMB-A44743C) and our journal name, which they will need when they contact our press office.

About one week before your paper is published online, we shall be distributing a press release to news organizations worldwide, which may very well include details of your work. We are happy for your institution or funding agency to prepare its own press release, but it must mention the embargo date and Nature Structural & Molecular Biology. If you or your Press Office have any enquiries in the meantime, please contact press@nature.com.

You can now use a single sign-on for all your accounts, view the status of all your manuscript submissions and reviews, access usage statistics for your published articles and download a record of your refereeing activity for the Nature journals.

If you have not already done so, we strongly recommend that you upload the step-by-step protocols used in this manuscript to the Protocol Exchange. Protocol Exchange is an open online resource that allows researchers to share their detailed experimental know-how. All uploaded protocols are made freely available, assigned DOIs for ease of citation and fully searchable through nature.com. Protocols can be linked to any publications in which they are used and will be linked to from your article. You can also establish a dedicated page to collect all your lab Protocols. By uploading your Protocols to Protocol Exchange, you are enabling researchers to more readily reproduce or adapt the methodology you use, as well as increasing the visibility of your protocols and papers. Upload your Protocols at www.nature.com/protocolexchange/. Further information can be found at www.nature.com/protocolexchange/about.

An online order form for reprints of your paper is available at https://www.nature.com/reprints/author-reprints.html. Please let your coauthors and your institutions' public affairs office know that they are also welcome to order reprints by this method.

Please note that <i>Nature Structural & Molecular Biology</i> is a Transformative Journal (TJ). Authors may publish their research with us through the traditional subscription access route or make their paper immediately open access through payment of an article-processing charge (APC). Authors will not be required to make a final decision about access to their article until it has been accepted. Find out more about Transformative Journals

<B>Authors may need to take specific actions to achieve compliance with funder and institutional open access mandates. For submissions from January 2021, if your research is supported by a funder that requires immediate open access (e.g. according to Plan S principles) then you should select the gold OA route, and we will direct you to the compliant route where possible. For authors selecting the subscription publication route our standard licensing terms will need to be accepted, including our self-archiving policies. Those standard licensing terms will supersede any other terms that the author or any third party may assert apply to any version of the manuscript.

In approximately 10 business days you will receive an email with a link to choose the appropriate publishing options for your paper and our Author Services team will be in touch regarding any additional information that may be required.

You will not receive your proofs until the publishing agreement has been received through our system.

If you have any questions about our publishing options, costs, Open Access requirements, or our legal forms, please contact ASJournals@springernature.com

Kind regards,
Florian

Florian Ullrich, Ph.D.
Associate Editor
Nature Structural & Molecular Biology
ORCID 0000-0002-1153-2040

Click here if you would like to recommend Nature Structural & Molecular Biology to your librarian:
http://www.nature.com/subscriptions/recommend.html#forms
